# CRISIS AFAR: an international collaborative study of the impact of the COVID-19 pandemic on mental health and service access in youth with autism and neurodevelopmental conditions

**DOI:** 10.1186/s13229-022-00536-z

**Published:** 2023-02-14

**Authors:** Bethany Vibert, Patricia Segura, Louise Gallagher, Stelios Georgiades, Panagiota Pervanidou, Audrey Thurm, Lindsay Alexander, Evdokia Anagnostou, Yuta Aoki, Catherine S. Birken, Somer L. Bishop, Jessica Boi, Carmela Bravaccio, Helena Brentani, Paola Canevini, Alessandra Carta, Alice Charach, Antonella Costantino, Katherine T. Cost, Elaine A Cravo, Jennifer Crosbie, Chiara Davico, Federica Donno, Junya Fujino, Alessandra Gabellone, Cristiane T Geyer, Tomoya Hirota, Stephen Kanne, Makiko Kawashima, Elizabeth Kelley, Hosanna Kim, Young Shin Kim, So Hyun Kim, Daphne J. Korczak, Meng-Chuan Lai, Lucia Margari, Lucia Marzulli, Gabriele Masi, Luigi Mazzone, Jane McGrath, Suneeta Monga, Paola Morosini, Shinichiro Nakajima, Antonio Narzisi, Rob Nicolson, Aki Nikolaidis, Yoshihiro Noda, Kerri Nowell, Miriam Polizzi, Joana Portolese, Maria Pia Riccio, Manabu Saito, Ida Schwartz, Anish K. Simhal, Martina Siracusano, Stefano Sotgiu, Jacob Stroud, Fernando Sumiya, Yoshiyuki Tachibana, Nicole Takahashi, Riina Takahashi, Hiroki Tamon, Raffaella Tancredi, Benedetto Vitiello, Alessandro Zuddas, Bennett Leventhal, Kathleen Merikangas, Michael P. Milham, Adriana Di Martino

**Affiliations:** 1 Autism Center, Child Mind Institute, 101 E 56Th Street, Third Floor, New York, NY USA; 2 Department of Psychiatry, School of Medicine, Trinity College Dublin, Dublin, Ireland; 3 Department of Psychiatry and Behavioural Neurosciences, McMaster University, Hamilton, ON Canada; 4 Unit of Developmental and Behavioral Pediatrics, First Department of Pediatrics, School of Medicine, National & Kapodistrian University of Athens, “Aghia Sophia” Children’s Hospital, Athens, Greece; 5 Neurodevelopmental and Behavioral Phenotyping Service, National Institute of Mental Health, Bethesda, MD USA; 6 Center for the Developing Brain, Child Mind Institute, New York, NY USA; 7 Autism Research Centre, Bloorview Research Institute, Holland Bloorview Kids Rehabilitation Hospital, Toronto, ON Canada; 8 Department of Paediatrics, University of Toronto, Toronto, ON Canada; 9 Medical Institute of Developmental Disabilities Research, Showa University, Tokyo, Japan; 10 Department of Pediatrics, School of Medicine, University of Toronto, Toronto, ON Canada; 11 Division of Paediatric Medicine, Hospital for Sick Children, Toronto, ON Canada; 12 Department of Psychiatry and Behavioral Sciences and Weill Institute for Neurosciences, University of California, San Francisco, CA USA; 13 Department of Biomedical Sciences, Section of Neuroscience & Clinical Pharmacology, University of Cagliari, Cagliari, Italy; 14 UOSD di Neuropsichiatria Infantile - Dipartimento di Scienze Mediche Traslazionali, Università Federico II di Napoli, Naples, Italy; 15 Department of Psychiatry, Hospital das Clinicas HCFMUSP, Faculty of Medicine, University of São Paulo (USP), São Paulo, Brazil; 16 Department of Health Sciences, Università Degli Studi Di Milano, Milan, Italy; 17 Epilepsy Center - Sleep Medicine Center, Childhood and Adolescence Neuropsychiatry Unit, ASST SS. Paolo E Carlo, San Paolo Hospital, Milan, Italy; 18 Department of Medical, Surgical and Pharmacy, Unit of Child Neuropsychiatry, University Hospital of Sassari, Sassari, Italy; 19 Department of Psychiatry, Hospital for Sick Children, Peter Gilgan Centre for Research and Learning, Toronto, ON Canada; 20 Department of Psychiatry, Temerty Faculty of Medicine, University of Toronto, Toronto, ON Canada; 21 Child and Adolescent Neuropsychiatric Unit, Foundation IRCCS Ca’ Granda Ospedale Maggiore Policlinico, Milan, Italy; 22 UFPR - Federal University of Paraná, Paraná, Brazil; 23 Department of Public Health and Pediatric Sciences, Section of Child and Adolescent Neuropsychiatry, University of Turin, Turin, Italy; 24 Department of Psychiatry and Behavioral Sciences, Graduate School of Medical and Dental Sciences, Tokyo Medical and Dental University, Tokyo, Japan; 25 Department of Precision and Regenerative Medicine and Ionian Area, (DiMePRe-J), University of Bari “Aldo Moro”, Bari, Italy; 26 Department of Psychiatry and Behavioral Sciences, University of California San Francisco, San Francisco, CA USA; 27 Department of Neuropsychiatry, Graduate School of Medicine, Hirosaki University, Hirosaki, Aomori, Japan; 28 Department of Psychiatry, Weill Cornell Medical College, Center for Autism and the Developing Brain, New York, NY USA; 29 Koishikawa Tokyo Hospital, Tokyo, Japan; 30 Department of Psychology, Queens University, Kingston, ON Canada; 31 The UCSF Center for ASD & NDDs, University of California San Francisco, San Francisco, CA USA; 32 School of Psychology and Psychiatry, Korea University, Seoul, South Korea; 33 Autism Research Centre, Department of Psychiatry, University of Cambridge, Cambridge, UK; 34 Centre for Addiction and Mental Health, Toronto, ON Canada; 35 Department of Psychiatry, National Taiwan University Hospital and College of Medicine, Taipei, Taiwan; 36 Department of Precision and Regenerative Medicine and Ionian Area, (DiMePRe-J), University of Bari “Aldo Moro”, Bari, Italy; 37 IRCCS Stella Maris Foundation, Calambrone-Pisa, Italy; 38 Child Neurology and Psychiatry Unit, Systems Medicine Department, University of Rome Tor Vergata, Rome, Italy; 39 39ADMiRE, Linn Dara Child and Adolescent Mental Health Services, Cherry Orchard Hospital, Ballyfermot, Dublin, Ireland; 40 Unita’ Operativa di Neuropsichiatria dell’ Infanzia e dell’ adolescenza, Lodi, Italy; 41 Keio University School of Medicine, Tokyo, Japan; 42 Department of Psychiatry, University of Western Ontario, London, ON Canada; 43 Thompson Center of Neurodevelopmental Disorders, University of Missouri, Columbia, MO USA; 44 Research Center for Child Mental Development, Graduate School of Medicine, Hirosaki University, Hirosaki, Japan; 45 Department of Clinical Psychological Science, Comprehensive Rehabilitation Science, Graduate School of Health Sciences, Hirosaki University, Hirosaki, Aomori, Japan; 46 Genetics Department/UFRGS, Medical Genetics Service/HCPA, Porto Alegre, Brazil; 47 Medical Physics, Memorial Sloan Kettering Cancer Center, New York, USA; 48 Division of Infant and Toddler Mental Health, Department of Psychosocial Medicine, National Center for Child Health and Development, Tokyo, Japan; 49 Child & Adolescent Neuropsychiatry Unit, “A.Cao” Paediatric Hospital, Cagliari, Italy; 50 University of Chicago, Chicago, IL USA; 51 Genetic Epidemiology Research Branch, Intramural Research Program, National Institute of Mental Health, Bethesda, USA; 52 Nathan S. Kline Institute for Psychiatric Research, Orangeburg, NY USA

**Keywords:** Mental health outcomes, Autism spectrum disorder, Neurodevelopmental conditions, Sleep, Behavioral problems, Prediction, Risk and resilience factors, COVID-19 pandemic, Public health

## Abstract

**Background:**

Heterogeneous mental health outcomes during the COVID-19 pandemic are documented in the general population. Such heterogeneity has not been systematically assessed in youth with autism spectrum disorder (ASD) and related neurodevelopmental disorders (NDD). To identify distinct patterns of the pandemic impact and their predictors in ASD/NDD youth, we focused on pandemic-related changes in symptoms and access to services.

**Methods:**

Using a naturalistic observational design, we assessed parent responses on the Coronavirus Health and Impact Survey Initiative (CRISIS) Adapted For Autism and Related neurodevelopmental conditions (AFAR). Cross-sectional AFAR data were aggregated across 14 European and North American sites yielding a clinically well-characterized sample of *N* = 1275 individuals with ASD/NDD (age = 11.0 ± 3.6 years; *n* females = 277). To identify subgroups with differential outcomes, we applied hierarchical clustering across eleven variables measuring changes in symptoms and access to services. Then, random forest classification assessed the importance of socio-demographics, pre-pandemic service rates, clinical severity of ASD-associated symptoms, and COVID-19 pandemic experiences/environments in predicting the outcome subgroups.

**Results:**

Clustering revealed four subgroups. One subgroup—*broad symptom worsening only* (20%)—included youth with worsening across a range of symptoms but with service disruptions similar to the average of the aggregate sample. The other three subgroups were, relatively, clinically stable but differed in service access: *primarily modified services* (23%), *primarily lost services* (6%), and *average services/symptom changes* (53%). Distinct combinations of a set of pre-pandemic services, pandemic environment (e.g., COVID-19 new cases, restrictions), experiences (e.g., COVID-19 Worries), and age predicted each outcome subgroup.

**Limitations:**

Notable limitations of the study are its cross-sectional nature and focus on the first six months of the pandemic.

**Conclusions:**

Concomitantly assessing variation in changes of symptoms and service access during the first phase of the pandemic revealed differential outcome profiles in ASD/NDD youth. Subgroups were characterized by distinct prediction patterns across a set of pre- and pandemic-related experiences/contexts. Results may inform recovery efforts and preparedness in future crises; they also underscore the critical value of international data-sharing and collaborations to address the needs of those most vulnerable in times of crisis.

**Supplementary Information:**

The online version contains supplementary material available at 10.1186/s13229-022-00536-z.

## Background

It is widely recognized that pediatric populations are vulnerable to sudden and pervasive disruptions in their daily life, such as those brought by the COVID-19 pandemic [1–4]. Those with neurodevelopmental disorders (NDD), broadly defined as childhood-onset chronic conditions characterized by atypical brain development [5–7], have been identified by parents, educators, clinicians, and policy makers alike, as requiring specific attention given their preexisting behavioral, emotional, and learning difficulties. The gamut of such difficulties is often observed in individuals with autism spectrum disorder (ASD). A neurodevelopmental disorder, ASD, is defined by impairments in social communication, and restricted and repetitive behaviors/interests that are often accompanied by comorbid neurological and psychiatric conditions, as well as varying degrees of speech-language and intellectual abilities [8–14]. Accordingly, a number of narrative articles and reviews have highlighted the burden on mental health that the disruptions in educational and treatment services, routine changes, and social isolation have posed for youth with ASD and related NDD [4, 15–22]. Here, we report on an international empirical effort aimed at assessing the heterogeneity of the impact of the COVID-19 pandemic in a large international sample of previously well-characterized youth with ASD and/or other NDD.

Our decision to focus on the heterogeneity of the pandemic outcomes was originally based on prior disaster research in the general population, showing that the degree of severity of prior mental illness, disaster exposure, as well as perceived risk, are predictors of negative outcomes [23–25]. These findings have been echoed, to some extent, in initial studies of the COVID-19 pandemic in ASD/NDD youth; these have documented care service disruptions [26–29] and negative mental health impact [4, 27, 29–40]. Most of them emphasized main group effects. Yet, findings suggest significant heterogeneity in outcomes. For example, 70% to 92% of those diagnosed with ASD/NDD have lost at least one special education or therapeutic service [26–28, 30], but the number of services lost during the pandemic has varied across individuals. Similarly, transitions to online service provision have occurred, but have only involved relatively smaller groups [26, 27]. In one study, approximately two-thirds of parents indicated that service disruptions negatively impacted their children’s functioning to varying degrees [27]. In parallel, studies documenting mental health difficulties varied in the symptom domain(s) examined, ranging from behavioral and/or emotional problems [30, 31, 36, 38, 39], ASD symptoms [28, 30, 39], living skills [32, 35], difficulties, and/or sleep disruptions [30, 32, 36, 40]. For example, the first study of the pandemic in children with ASD [32] reported more intense and frequent behavioral problems (in 36% and 42% of the sample, respectively). An additional study reported the onset of disruptive behaviors, anxiety, sleep problems, and irritability in varying proportions of children, ranging from 11 to 28% of the sample [34]. Overall, the scientific literature suggests that, for ASD/NDD youth, different profiles of changes in mental and service access from pre- to pandemic time occur. However, the concomitant pattern of variability in these two domains remains unclarified.

Understanding outcome heterogeneity in both service and clinical symptom changes is a necessary step to identify which individuals have greater needs. This may also facilitate the identification of protective and risk factors. Toward these goals, we adapted the Coronavirus Health and Impact Survey Initiative (CRISIS) [25] for Autism and Related Neurodevelopmental Conditions (AFAR). CRISIS was originally designed to capture the multifaceted nature of risk in the general population during the COVID-19 pandemic by quantitatively assessing life changes and perceived risk about COVID-19, as well as mental health before and during the pandemic. Previous work in the general population established the psychometrics of CRISIS as well as its feasibility in delineating distinct life stress profiles and their predictive role in mental health outcomes [25, 41]. The CRISIS adaptation for autism and related neurodevelopmental conditions aimed to quantify both changes in symptom domains affected by or known to impact daily life, along with changes in therapeutic services. Preserving the original structure of CRISIS also allowed a comprehensive assessment of a range of disaster- and clinically related potential predictors of pandemic-related outcomes—a topic that is emerging in the literature, likely becoming a critical focus in years to come [28, 32, 34].

To enhance the scope of AFAR assessments, we formed an international, collaborative network of investigators to collect AFAR data in clinically well-characterized youth with ASD/NDD diagnoses. Unlike most prior ASD/NDD pandemic-related work which samples relatively homogeneous geographical regions, our aggregating data from multiple, international sites offered a naturalistic observational framework to quantify the degree of disaster exposure (i.e., new COVID-19 case rates and local containment measures) as a potential predictor of outcomes. This complemented the AFAR assessment of pandemic-related experiences, pre-pandemic mental health and child characteristics, thus yielding a comprehensive investigation of putative predictors of the pandemic’s outcomes.

Additionally, the aggregation of data across the AFAR network allowed us to rapidly generate a sample of individuals with ASD/NDD richly characterized by clinicians, at a scale not readily achievable otherwise. Given the multidimensional nature of the outcomes and predictors examined, we leveraged multivariate data-driven approaches in a large sample (*n* = 1275) of cross-sectional AFAR data collected over the initial phase of the COVID-19 pandemic. Against this background, the present study aimed to identify potential subgroups of outcome and their predictors to inform recovery efforts and prepare for future crises.

## Methods

### Survey development and structure

A workgroup of ASD/NDD experts (ADM, LG, SG, PP, AT, BV) led the adaptation from the CRISIS Parent/Caregiver Baseline Form. Adapting the caregiver survey was prioritized in order to immediately capture the acute phase of the pandemic across the widest possible range of ASD/NDD youth using the same method. The adaptation aimed to include assessments of clinical domains relevant to ASD/NDD and services, while maintaining the existing structure of CRISIS [25, 42]. At the time, empirical evidence on the impact of disasters on ASD/NDD was limited to one study reporting worsening in adaptive functioning [43]. Therefore, along with adaptive skills, we prioritized the assessment of symptoms known to be affected by or to impact adjustment needs. These encompassed restricted and repetitive behaviors/interests (RRB) [44–46], externalizing, internalizing symptoms, and sleep problems that often co-occur in ASD/NDD [8, 12]. Parent/caregiver questions were developed to target observable behaviors rather than attempting to seek reports on internal states. In keeping with this goal and to contain the survey’s length, new questions on a range of co-occurring psychiatric symptoms replaced the Mood State domain. The Substance Use domain was also removed given that it is more accurately measured by self-report, which is beyond the scope of the present study [47, 48]. Like CRISIS, symptoms were rated on a Likert-scale based on (1) the three months prior to the start of COVID-19 pandemic in the respondent’s geographical area and (2) the two weeks prior to completion (*Prior* and *Current* time points, respectively).

To evaluate changes in service access, we derived items from a survey developed during the pandemic and piloted with people with syndromic intellectual disabilities and their caregivers [26]. Questions queried changes in therapeutic services typically received both within and outside school settings in the respondents geographical area, following the start of the pandemic. The remainder of the original CRISIS was unchanged, except for some rewordings or additional response options (e.g., sleep problems) as summarized in Table 1 and detailed in Additional file 1: Methods.

**Table 1:** CRISIS AFAR Parent Baseline Survey v0.5.1 (3–21 years): Domains and Items

	Shared with CRISIS					AFAR-specific			
Domain	Background	Coronavirus/COVID-19 Health/Exposure Status	Life Changes^d^	COVID-19 Worries^d^	Behavior/ Media^d^	Adaptive Living Skills^c,d^	RRB^c,d^	Co-Occurring Problem Behavior^b,c,d^	Services^c^
Item Descriptions	Age, sex at birth, gender	Any family impact	Food insecurity^a^	Worried self, other	Bed time weekdays, weekends	Entertains self	Repetitive motor mannerisms	Hyperactivity, difficulty staying on task	School
	Ancestry	Family member COVID-19 diagnosed	Financial difficulty	Physical worries	Falling and staying asleep^c^	Structures time	Sensory seeking	Getting angry/ losing temper	Outside school
	Health insurance, government assistance	Last 2-week exposure, symptom count	Housing instability concern	Reading and talking^a^	Hours of sleep weekdays, weekends	Self-care	Rituals or routines	Verbal aggression	
	Urbanicity, household	School closed, job loss	Positive changes	Mental worries	Exercise^a^, time outdoors	Mealtime Independence	Insist others maintain routines	Physical aggression	
	Essential workers		Time outside home		TV and media, video games		Highly restricted/ strong interest	Deliberately injuring self	
	Child’s physical health, neuropsychiatric diagnoses^a^		Difficulty distancing		Social media^b,c^		Adjustment to changes	Disobedient/ arguing	
	Current grade, level/employment,^a^		Event cancellation stress^a^		Tech to engage with family^b,c^			Crying easily	
	Educational setting^c^		Stay-at-home stress		Tech to engage with peers^b,c^			Worry social situations	
	Respondent relation to child, age, and education^a^		Hopefully end^a^					Worry separation from caregiver	
	Other caregiver’s education^a^							Fearful	
# of items	20	6	7	5	13	4	6	11	7
Time targeted	At time of completion	Last 2 Weeks			Three months prior to pandemic and last 2 weeks				Pre-pandemic and current

CRISIS AFAR retained CRISIS key content and structure (e.g., last 2 weeks for COVID-19 Impact, COVID-19 Worries and Life Changes domains, the 3 months prior to the pandemic start in the respondent’s geographical area and last 2 weeks for the other Likert-scale items).a: Item slightly reworded; b: Item not asked for children younger than 5 years; c: Domain/item added in CRISIS AFAR. See Main Text and Additional File 1: Methods for more details on the adaptation process, as well as Additional File 1: Methods and Tables S5-S6 for the AFAR factor structure; d: Likert-scale items. See http://www.crisissurvey.org/crisis-afar/ for complete survey

Like CRISIS, questions of the parent/caregiver survey were developed for individuals aged five to 21 years; a later review identified a subset of questions developmentally applicable for children as young as three years. The initial adaptation was developed in English and then translated into five other languages and updated with rewordings in consultation with the larger AFAR network. The final version 0.5.1 of AFAR Parent/Caregiver Baseline Form (3–21) included 96 independent items, with 34 questions asked twice for *Prior* and *Current* time points. Clinical contacts with caregivers of youth with ASD/NDD during the acute phase of the pandemic informed the adaptation process; at its completion, several sites reached out to caregivers of ASD/NDD youth to review the survey; they favored its distribution for data collection (see Additional file 1: Methods for more details). AFAR is freely available for use by other investigators [42].

Following the AFAR data collection, we used exploratory and confirmatory factor analyses to identify the underlying structure of each of the domains developed a priori and to yield summary scores for quantitative investigations (see Additional file 1: Methods for more details). As detailed in Additional file 1: Results and in Tables S5 and S6, factor analyses yielded a stable and replicable factor structure, including a single factor for Adaptive Living Skills, two factors RRB largely reflecting lower- and higher-order RRB [46, 49], and four for Co-occurring Problem Behavior (i.e., Anxiety/Affect, Oppositional Behavior, Sleep Problems, Activity/Attention), among the AFAR-specific domains. Further, consistent with the general population [25], results yielded a single COVID-19 Worries factor, and multiple factors for the Life Changes and Behavior/Media domains (two and five, respectively).

### Data collection and selection

**Fig. 1:**
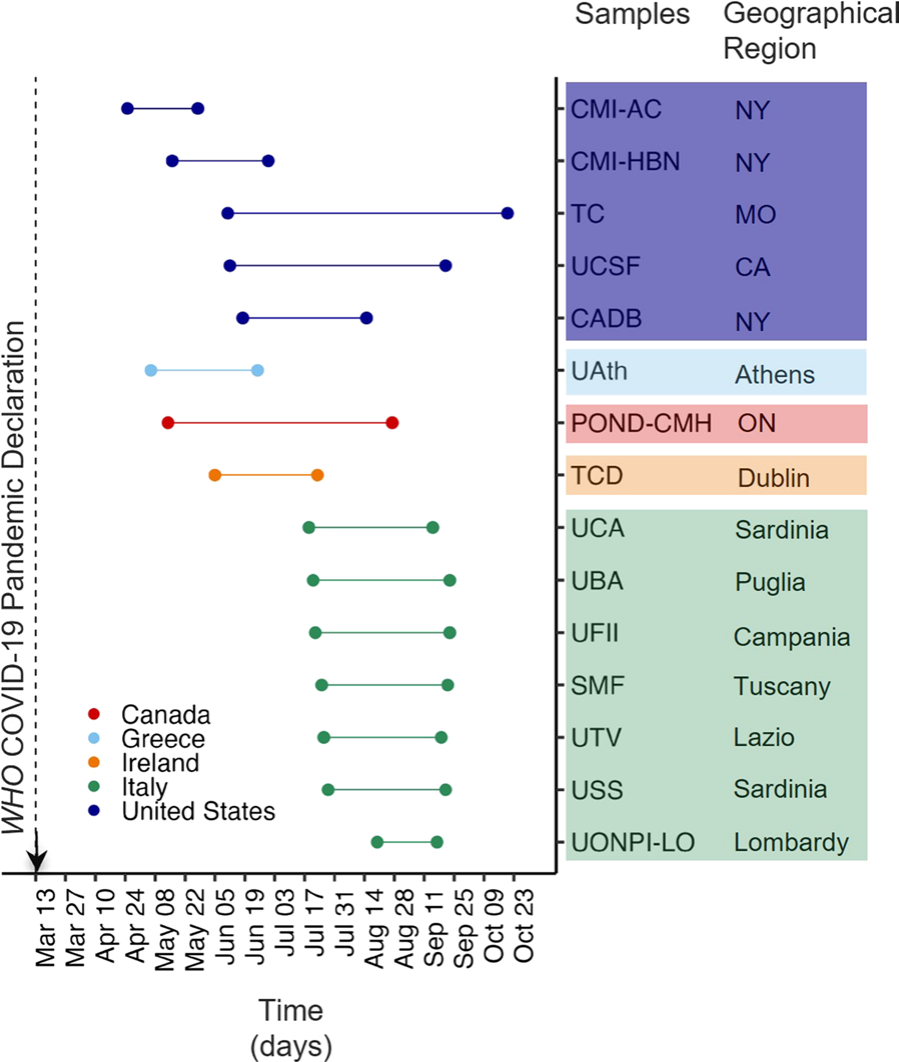
Data collection times across contributing samples. Data collection time periods for each contributing sample are color coded by country. Specific geographical regions for each sample are also indicated as state, or region. See Supplementary material in Additional file 1: Methods and Table S1 for details on data collection protocols. *NY*, New York; *MO* Missouri; *CA* California; *ON* Ontario; *WHO* World Health Organization

AFAR Parent/Caregiver Baseline surveys were collected in 15 samples at 14 research and/or clinical institutions in Europe and North America. Data were collected cross-sectionally over the first six months of the pandemic (April–October 2020; Fig. 1, Additional file 1: Methods and Table S1). Parents of children aged three to 21 years with previously established clinician-based DSM-IV/5 [6, 50] or ICD-10 [5] diagnosis of ASD and/or other NDD were invited to complete AFAR. As discussed below, only data from individuals aged five years and older are included in the present analyses.

Along with diagnostics, when feasible, previously collected information on intellectual functioning and symptoms was shared. IRB approval for collection and sharing of de-identified AFAR and related data was obtained at each institution. For the present analyses, only data from AFAR surveys with available AFAR responses for the variables used in clustering analyses and completed within the time interval of 90% of a given sample were included. This served to minimize outliers regarding to COVID-19 infection rates and related responses.

### Analyses

#### Overview

As shown in Table 2, to assess the heterogeneity of outcomes in symptom and service changes between prior and pandemic times, primary analyses leveraged data-driven hierarchical agglomerative clustering [51, 52] Clustering of the pandemic symptom changes was complemented by analyses of symptom and service changes across the whole aggregate sample (i.e., the sample derived from aggregating all eligible data) intended to further aid interpretation of results and comparisons with the literature, as well as to underscore the relevance of assessing heterogeneity. These whole-group-level analyses included repeated measures MANCOVA for symptom severity, measures of central tendency were used for access to services (Additional File 1; Methods). Following the identification of distinct outcome subgroups, we assessed and ranked a range of variables indexing pre-pandemic characteristics, as well as pandemic-related experiences and environments as their predictors. Analyses were corrected for multiple comparisons using false discovery rate (FDR) at *q* < 0.05, as applicable. The codes used in analyses are available in GitHub [53].

**Table 2:** Overview of analytical strategy and key results

Goal(s) and domains targeted		Main Analytic methods	Findings
Assessment of the pandemic impact on changes of the AFAR-based symptom ratings (i.e., Adaptive living skills, lower-order RRB, higher-order RRB, activity/inattention, oppositional, anxiety/affect, sleep problems) and services (lost or modified at and outside school) [Table 1, Additional file 1: Table S6]	Pandemic outcome subgroups identification and characterization	Hierarchical clustering	Four outcome subgroups with differing profiles of change relative to the aggregate’s average change: -Broad symptom worsening only (20%) -Primarily modified services (23%) -Primarily lost services (6%) -Average symptom/service changes (53%) [Figs. 4 and 5, Additional file 1: Table S7, Fig. S3]
	Whole (aggregate) sample main effects analyses	One-way repeated measures MANCOVA (within-subject factor time; covariate: contributing sample); Post hoc one-way repeated measures ANCOVA for each symptom factor	Significant effect of time was driven by worsening sleep problems ratings, other symptoms did not reach statistical significance [Fig. 5]
		Central tendency descriptive measures	On average, lost 1 service and continued 1 other at and outside school [Additional file 1: Table S7]
Prediction of outcome subgroup membership across 20 features including pre-pandemic variables (e.g., service at and outside school, child, and family’s characteristics), pandemic-related experiences (e.g., COVID worry) and environment (containment measures) [Fig. 2, Additional file 1: Table S2, Fig. S4]		Random Forest classification, ranking feature importance indexed by out-of-bag-error (OOBE)	81% classification accuracy. Pre-pandemic services in and outside school, Sringency index, Lifestyle Stress, COVID Worries, new COVID infections and age were top predictors (OOBE 16–1%). Other features had negligible importance (< 1%). Each outcome subgroup had distinct profiles of increases or decreases across the top predictors [Fig. 6, Table 3, Additional file 1: Table S7]

#### Hierarchical clustering

Outcome heterogeneity was assessed across 11 features indexing symptom and service changes pre-pandemic to pandemic time using agglomerative hierarchical clustering [51, 52]. Among these features, seven features reflected changes in clinically relevant symptoms for ASD/NDD. They indexed differences between *Current* and *Prior* scores on the symptom domains that were empirically identified with factor analyses (see Additional file 1: Methods and Tables S5–S6). The remaining four features included the total number of services that were either lost or continued within and outside school. All scores were converted to standard *z* scores prior to clustering; thus, clusters (i.e., subgroups) were characterized by their profile of deviation from the aggregate sample average. The optimal cluster solution was determined using *NbClust* [54], according to a majority rule among multiple goodness-of-fit measures.

#### Random forest

**Fig. 2:**
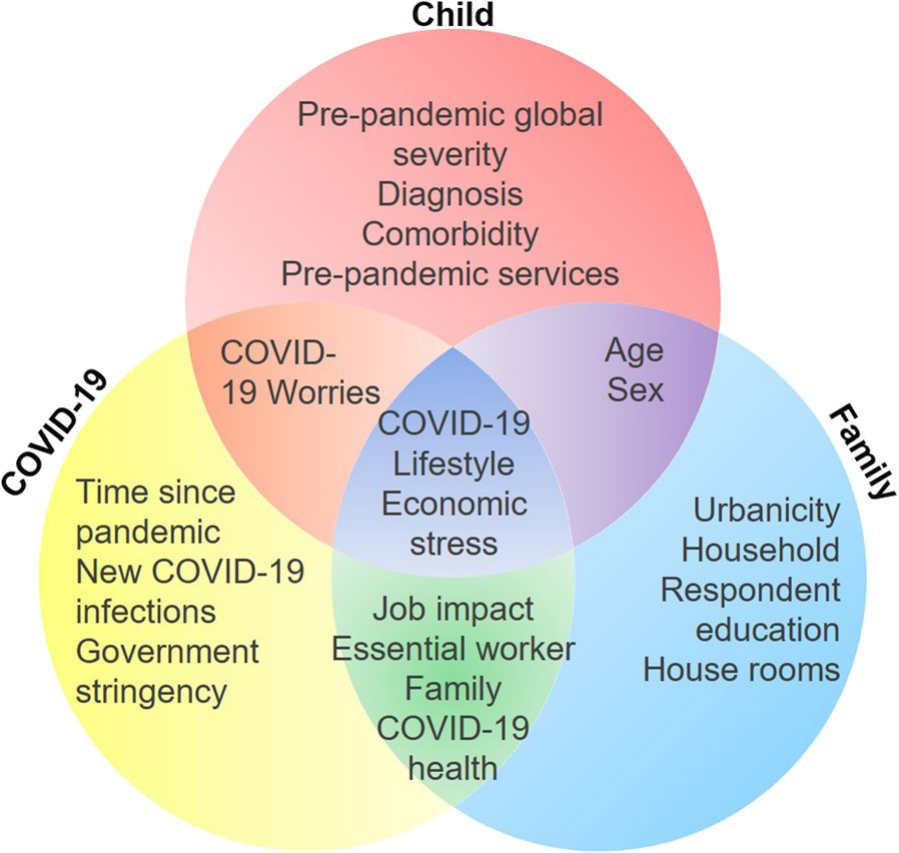
Features selected for predicting COVID-19 Impact Subgroups. The Venn diagram shows the 20 features examined as potential predictors of the four COVID-19 impact subgroups with random forest classification. Each feature is organized across three partially overlapping domains: child characteristics before the pandemic (red); COVID-19 pandemic experiences and environment (yellow); and family/household characteristics (blue)

To assess the relative contribution of multiple variables (i.e., features) as predictors of the outcome subgroups identified in hierarchical clustering, we used random forest classification. As depicted in Fig. 2, we assessed 20 features including family and child variables measuring pre- and pandemic-related experiences derived from AFAR, prior clinical characterization at each contributing sample (e.g., ASD vs. non-ASD NDD, number of psychiatric comorbidities), as well as COVID-19 pandemic environmental markers, empirically derived, for each individual, from open-data sources providing information by geographical area over time [55–57]. The variables derived from parent responses in AFAR included: socio-demographics, the child’s pandemic experiences, pre-pandemic services received, and a summary measure of clinical severity *Prior* (baseline) to the pandemic. This global severity summary score, computed across the seven AFAR symptom domains, was significantly correlated with previously collected standardized symptom measures (see Additional file 1: Fig. S4). The COVID-19 pandemic environmental markers included government responses, and new COVID-19 infection rates for each child in their geographical area at the time of the data collection. Briefly, to quantify new infection rates in a given child’s geographical area at the time of the AFAR data collection, we used the publicly available from Our World in Data’s (OWID) COVID-19 tracker [56] and COVID-19 European regional tracker [57]. To quantify government responses to the COVID-19 pandemic, for each child at the time of the AFAR data collection in their geographical area, we used the government stringency (GS) index computed by the Oxford Coronavirus Government Response Tracker [55]. The GS index combines metrics of infection containment and public information campaigns. It ranges from 0 to 100, higher scores reflect stricter government policies, and it is provided by day, in each territory. See Additional file 1: Methods for more details.

We quantified the relative predictive role of the 20 features examined following a previously established analytical framework [58–60]. Briefly, using a permutation importance method [60], we indexed importance as the average out-of-bag-error (OOBE), computed across 4000 bootstrapped samples (2/3 training, 1/3 testing). The larger the OOBE value, the greater the importance for a given feature. These features were ranked in decreasing order of importance according to the obtained OOBE values (see Additional file 1: Methods for details).

## Results

### Aggregate sample characteristics

**Fig. 3:**
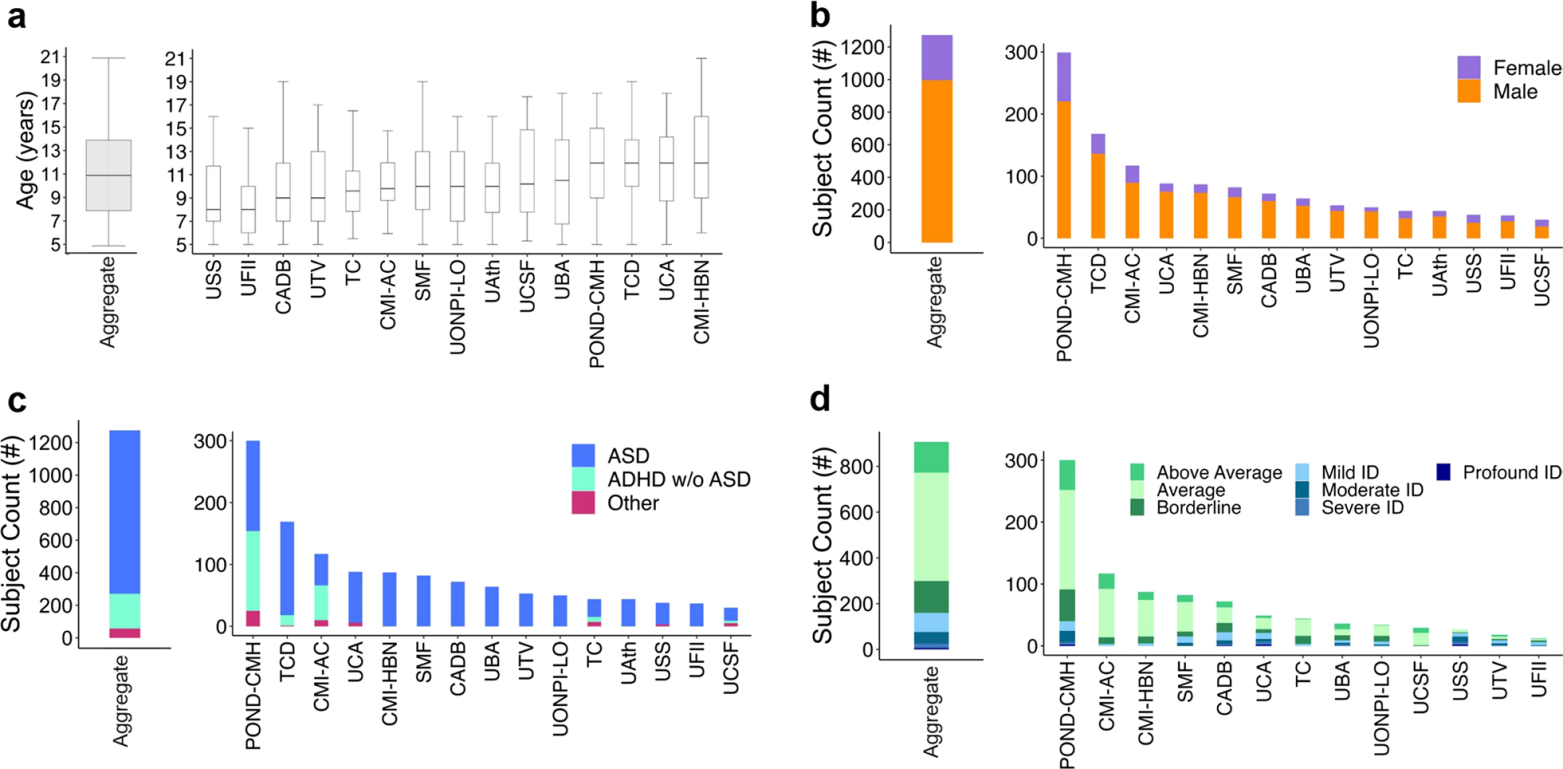
Characteristics of aggregate and each contributing sample. Age distribution (box plots), proportion of males and females, primary diagnoses, and intellectual functioning (stacked bar plots) are depicted for each of the n = 15 contributing samples, as well as for the aggregate sample (i.e., the dataset resulting from combining all contributing samples). *CMI-AC* Child Mind Institute-Autism Center; *CMI-HBN* CMI Healthy Brain Network; *TC* Thompson Center; *UCSF* University of California San Francisco; *CADB* Center for Autism and Developing Brain, Weill Cornell Medical College/New York Presbyterian Hospital; *UAth* University of Athens, National & Kapodistrian University of Athens, School of Medicine, First Department of Pediatrics, Unit of Developmental and Behavioral Pediatrics. “Aghia Sophia” Children’s Hospital; *POND-CMH* Province of Ontario Neurodevelopmental Network, COVID Mental Health collaboration; *TCD* Trinity College Dublin; *UCA* University of Cagliari, Child & Adolescent Neuropsychiatry Unit, A.Cao Paediatric Hospital; *UBA* University Bari, Child Neuropsychiatry Unit, Policlinic of Bari; *UFII* University of Naples Federico II, Child and Adolescent Neuropsychiatry Unit; *SMF* Stella Maris Foundation, University of Pisa; *UTV* University Tor Vergata; *USS* University of Sassari, Child Neuropsychiatry Unit, Azienda Ospedaliero-Universitaria; *UONPI-LO* Unita' Operativa di Neuropsichiatria dell’ Infanzia e dell' adolescenza, Lodi; *ASD* Autism Spectrum Disorder, *ADHD* Attention-Deficit/Hyperactivity Disorder; and *ID* Intellectual Disability. See Table S1 and Methods in Additional file 1 for details on data collection protocols

Data from 1275 youth, 5- to 21-year-old, aggregated across the 15 contributing samples met inclusion criteria for subsequent analyses. As shown in Figs. 1 and 3, contributing samples were from Europe (49%) and North America (51%). They included seven samples collected across research and clinical institutions in Italy, one each from Greece and Ireland, four samples collected in the USA (two in New York, and one each in California, and Missouri), and one sample collected across five centers in Ontario, Canada, as part of a COVID-19 multi-network collaboration in pediatrics, including the Province of Ontario Neurodevelopmental Disorders COVID Mental Health collaboration (POND-CMH) Network [61–63]. Key demographics (age and sex a birth), diagnosis, and intellectual functioning are summarized in Fig. 3 for the aggregate and each contributing sample. Most individuals (79%, *n* = 1004) met diagnostic criteria for ASD; 17% (*n* = 214) had ADHD without ASD, and 4% (*n* = 57) had other NDDs, without ASD. Reflecting the known male bias of ASD/NDD [64, 65], the sample predominantly included males (88%). Among 938 (80%) youth with available intelligence estimates, 62% (*n* = 624) were in the Average/Above Average range, 14% (*n* = 141) in the Borderline range, and 24% (*n* = 173) had mild to profound intellectual disability. Further, over half (64%, *n* = 811) of the caregivers had at least a college degree; the remaining had either a high school degree (30%), or elementary education (6%). Fifty-six percent of the aggregate dataset was of European/British ancestry (see Additional file 1: Table S4). Information on prior (i.e., baseline) AFAR symptom factor severity and number of services received within and outside school are in Fig. 5; complementary characteristics are in Additional file 1: Results, Figures S1, S2 and Tables S2–S4.

### Pandemic outcome subgroups

**Fig. 4:**
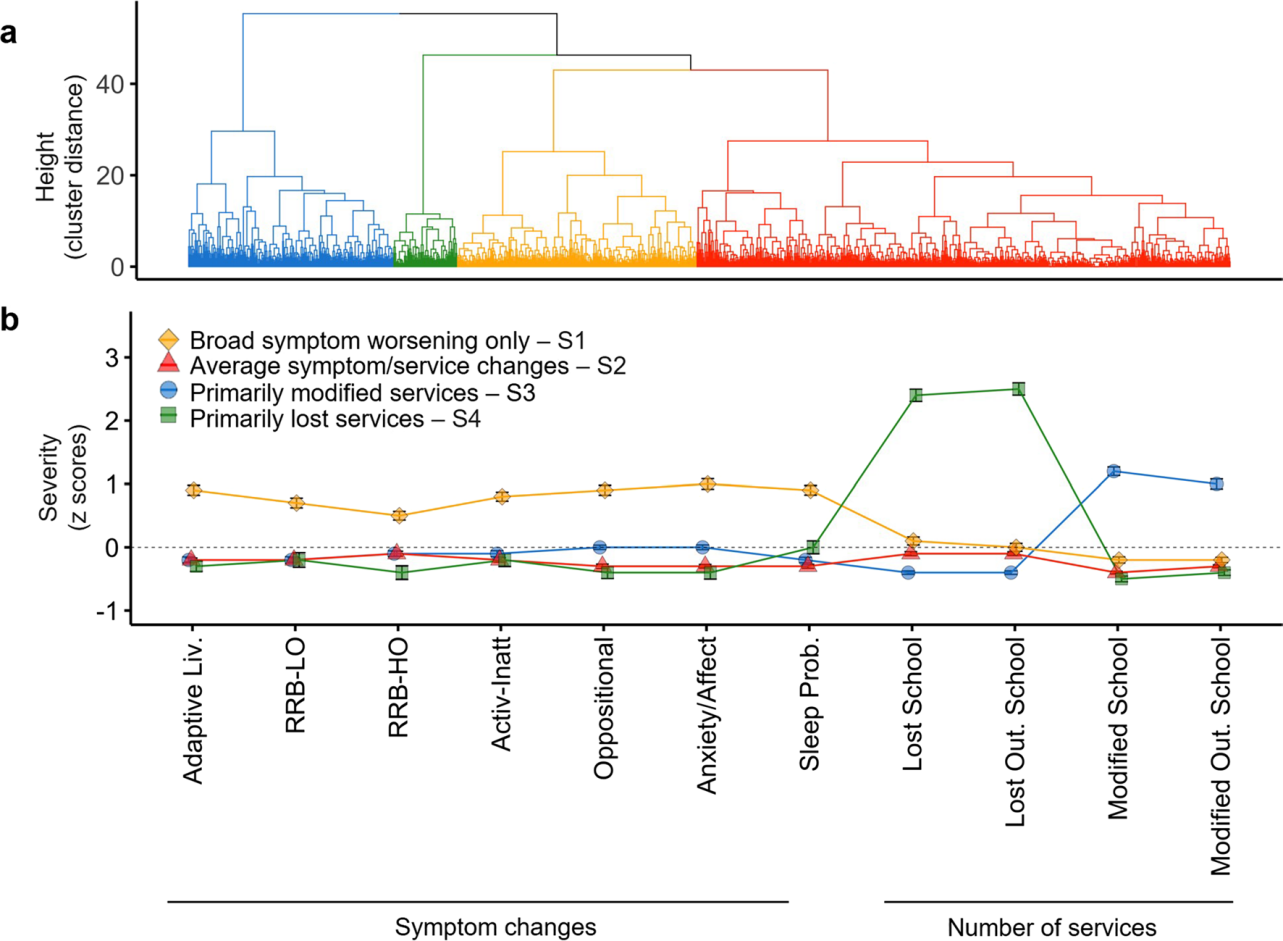
Clustering results and COVID-19 Impact Subgroup Patterns. **a** The dendrogram shows the optimal 4-cluster solution of COVID-19 Impact including: *broad symptom worsening only* (*n* = 251; 20%, yellow), *primarily modified services* (*n* = 293; 23%, blue), *primarily lost services* (*n* = 78; 6%, green), and *average symptom/service changes* (*n* = 653; 53%, red). **b** Groups means and standard error bars of the *z* scored symptom factor changes (difference between Current and Prior scores; or Δ) and number of services lost or modified in and outside (Out.) of school are shown for each cluster (i.e., outcome subgroup). The dotted gray horizontal line at a z score 0 represents the average of the aggregate sample (*N* = 1275) across each variable examined. Abbreviations: Adaptive Liv., Adaptive Living skills; RRB-LO, Restricted and Repetitive Behaviors—Lower Order; RRB-HO, Restricted and Repetitive Behaviors—Higher Order; Activ-Inatt, Activity Inattention; Sleep Prob., Sleep Problems; Out., Outside; and Mod., Modified

**Fig. 5:**
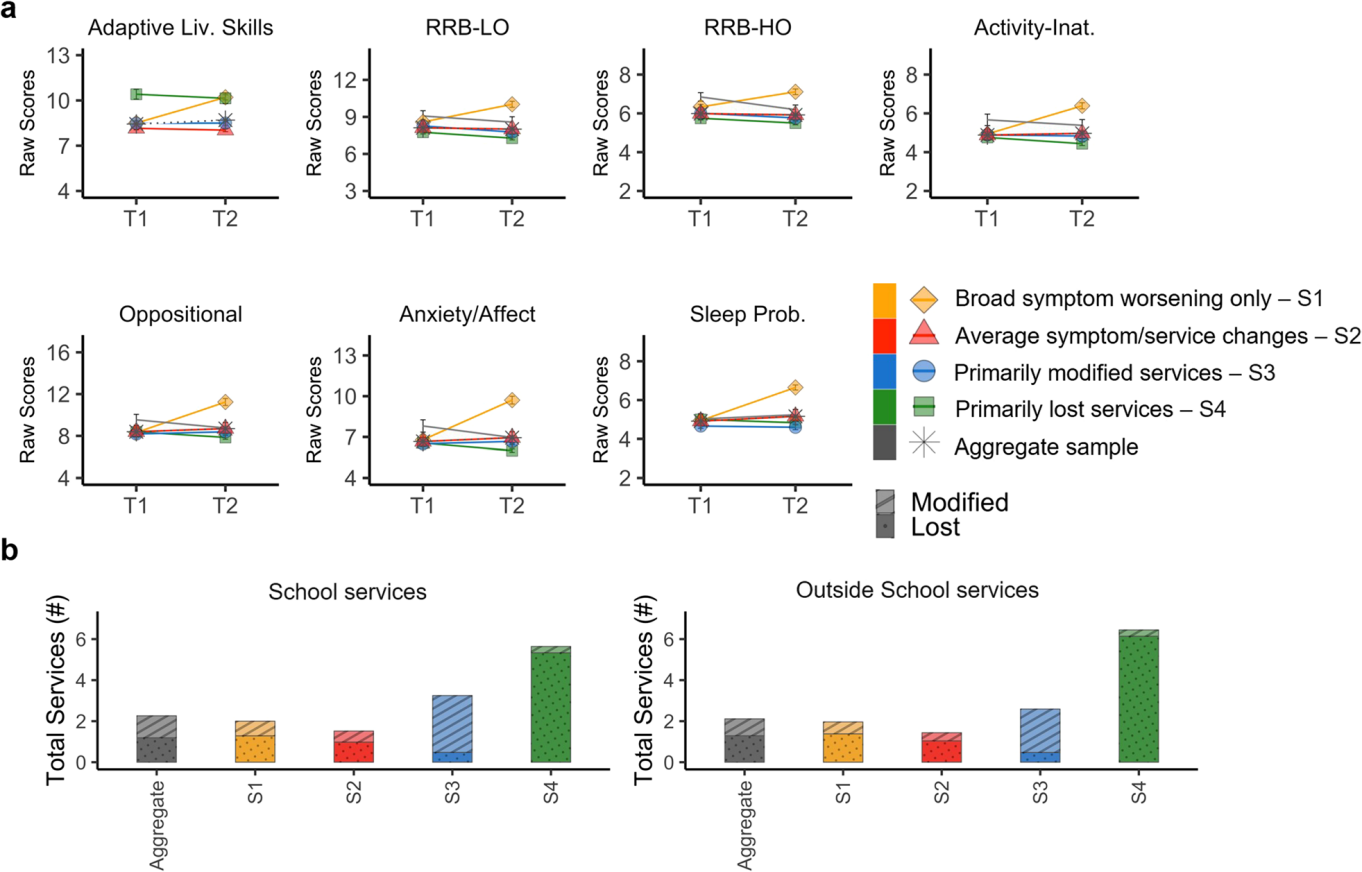
Symptom and service access change pattern. **a** For each of the four outcome subgroups (color coded in the legend) and the aggregate sample (in gray), plots depict groups means and standard error bars of prior (T1) and current (T2) symptom raw scores across the seven factors examined. High scores indicate greater severity/impairment. The scores from the adaptive living skills domain were multiplied by minus 1 in order to follow the same direction as the other factors. **b** The bar height represents the mean total number of services received prior to the pandemic for each subgroup (color coded in the legend), as well as for the aggregate dataset (gray) at school (left plot), or outside school (right plot). The dotted pattern within each stacked bar illustrates the group mean number of services lost, the striped pattern depicts the group mean number of services modified

Based on the *NbClust* approach, the four-cluster solution resulting from hierarchical clustering was optimal (see Figs. 4 and 5 and Additional file 1: Table S7). In other words, clustering analysis yielded four subgroups of ASD/NDD youth capturing distinct outcome profiles of symptom and service changes (Fig. 4). Based on their profile of deviations from the aggregate’s average of symptoms and/or service changes, the four outcome subgroups were defined as: *broad symptom worsening only* (20%), *primarily modified services* (23%), *primarily lost services (*6%), and *average symptom/service changes* (53%)*.* The subgroup defined as *broad symptom worsening only* was characterized by worsening across all symptom domains as indexed by z scores ranging between 1 and 3 (i.e., 1 to 3 standard deviations above the aggregate’s mean) but by marginal service changes relative to the aggregate’s average (i.e., within 0.5 standard deviation from the aggregate’s average). The three remaining subgroups, totaling *n* = 1024 (80% of the aggregate), showed symptom changes within the aggregate sample’s average (*z* scores < 0.5) but differed in service access. As indicated by their name, one subgroup had the most services modified, another had the most services lost, and the third subgroup had the number of services lost and continued like those of the aggregate. These differential profiles of changes in symptom and services were confirmed by one-way ANOVAs and Tukey pairwise group mean comparisons (FDR *q* < 0.05; Additional file 1: Table S7). Since, as expected by our naturalistic study design, differences in subgroup distributions existed between contributing samples (Additional file 1: Fig. S3), we conducted follow-up analyses including “contributing sample” as covariate. Statistical group differences were unchanged. Similarly, secondary cluster analyses on data subsets distinct by survey completion rates yielded similar results (Additional file 1: Results).

Finally, Additional file 1: Table S8 provides descriptive statistics and subgroup comparisons regarding key demographics (age and sex at birth) and intellectual functioning. As detailed in Supplementary Results in Additional file 1, the four subgroups did not significantly differ in sex distribution; there was a main effect of subgroup for age with the subgroup *average service and symptom change* being significantly older (11.6 ± 3.5 years) than the three other subgroups which included children in mid-childhood (mean and SD range across subgroups: 9.7–10.8 ± 3.1–3.8 years). Most youth fell in the average/above average intelligence category across subgroups except for the *primarily lost services* subgroup, which was characterized by significantly greater intellectual impairment.

#### Whole-sample average impact

In the aggregate sample, one-way repeated measures MANCOVA yielded a significant main effect of time across the seven AFAR symptom factor scores (*F*_(7,2548)_ = 77.40, FDR corrected *p* < 0.001). Follow-up comparisons for each symptom factor revealed statistical significance worsening of sleep problems (prior = 4.9 ± 2.1, current = 5.2 ± 2.3; *F*_(1,2548)_ = 8.158, FDR corrected *p* = 0.03; see Fig. 5). Over the initial phase of the pandemic, on average the aggregate sample lost 1.2 ± 1.7; 1.3 ± 2 services, within and outside school. One service, on average, continued either within or outside school (1.1 ± 1.4; 0.8 ± 1.3, respectively), mostly continuing via telehealth/email (88% in school and 69% outside of school). More details on service changes are in Additional file 1: Table S7 and Supplementary results.

### Predictors of outcome subgroup

**Fig. 6:**
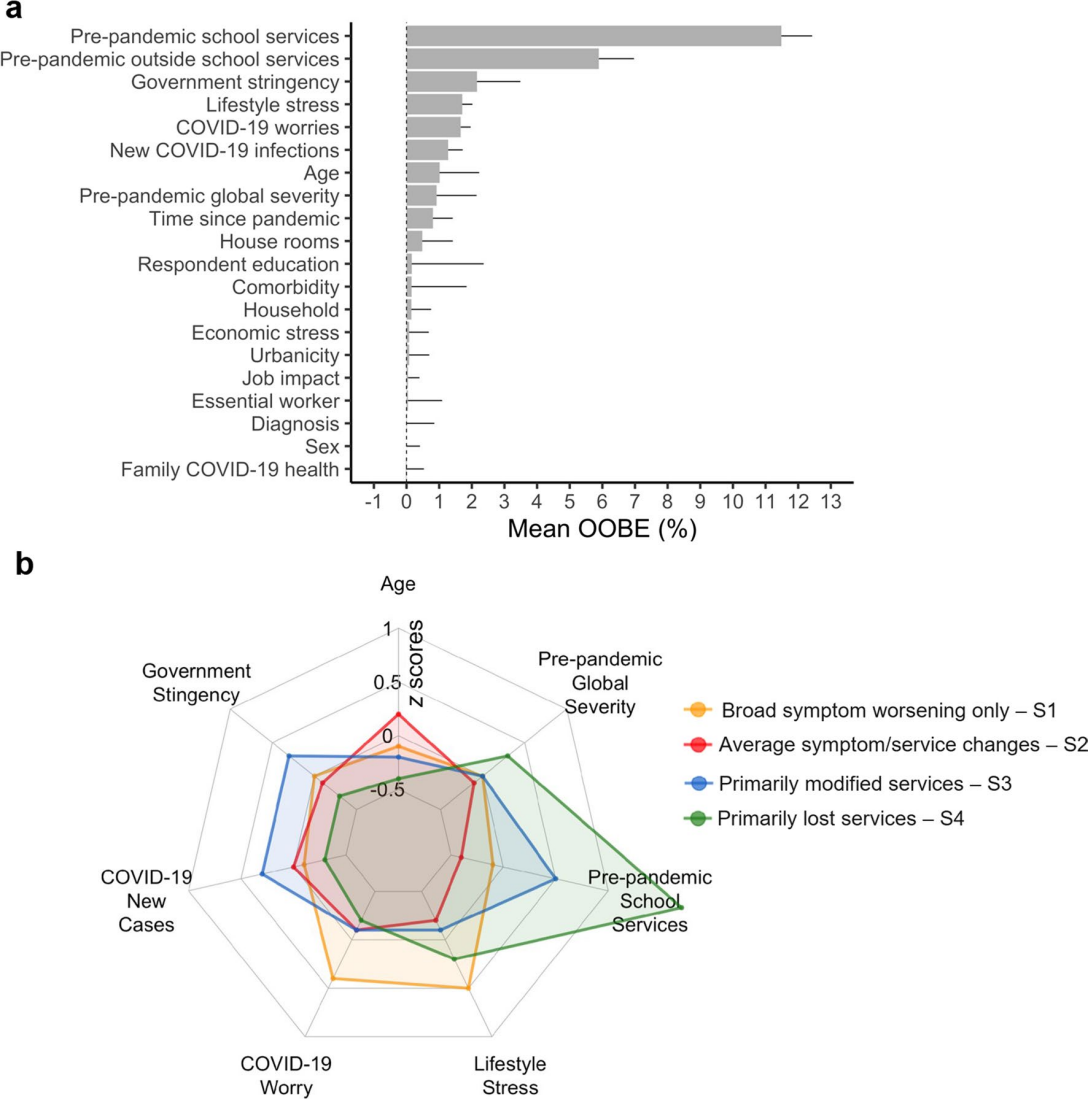
Random forest feature importance ranking and top-ranked features by subgroup. **a** Feature (predictor) ranking by importance indexed by mean out-of-bag errors (OOBEs) is shown in descendent order. **b** The radial plot shows the *z*-scored group means across the eight top-ranked predictors color coded by outcome subgroup (Yellow** = ***broad symptom worsening only*; Red = *average symptom/service changes*; Blue = *primarily modified services*; Green = *primarily lost services*)

The RF classification model predicted subgroup membership with 81% accuracy (precision/sensitivity = 82%, recall/specificity = 75%). The top-ranked predictor (OOBE: 12%) was the number of services received at school before the pandemic (Fig. 6a). Six predictors followed, with OOBE ranging from 6 to 1%. In rank order, they included the number of services received in and outside of school, GS index, Lifestyle Stress, COVID-19 Worries, new COVID-19 infections, and child’s age. The remaining features had OOBE < 1% and, thus, were considered negligible predictors.

Across the top seven predictors, each outcome subgroup was characterized by unique combinations of increases or decreases relative to the other subgroups. For example, the *primarily modified services* subgroup had a greater number of services before the pandemic relative to the *broad symptom worsening only* and to the *average symptom/service changes* subgroups. This latter subgroup, however, had lower ratings of COVID-19 Worries and Lifestyle Stress and included older youth in comparison with the other three subgroups. The *primarily lost services* subgroup had a similarly higher number of pre-pandemic services received but had the lowest GS index and COVID-19 new infection rates, as well as lower COVID-19 Worries and Lifestyle Stress relative to the other subgroups. Notably, the *broad symptom worsening only* subgroup had the highest COVID-19 Worries and Lifestyle Stress ratings, even with lower infection rates and GS index relative to the *primarily modified services subgroup*. These subgroups’ differences across predictors were confirmed by one-way ANOVAs and Tukey pairwise group mean comparisons (FDR *q* < 0.05; see Table 3). Since differences in subgroup distributions existed between contributing samples (Additional file 1: Fig. S3), we conducted follow-up analyses covarying for “contributing sample” in comparisons for the total baseline services received, Lifestyle Stress, COVID-19 Worries, and child age, but not for GS index and COVID-19 rates, as they reflect known differences across contributing samples per study design; statistical subgroup differences remained unchanged.

**Table 3:** Group means of top eight predictors in the aggregate sample and by subgroups

Characteristic	Aggregate	S1	S2	S3	S4	ANOVA subgroup comparisons		
Random Forest top-ranked predictors, M (SD)	*N* = 1244	(*n* = 249, 20%)	(*n* = 637, 51%)	(*n* = 283, 23%)	(*n* = 75, 6%)	*F*_(3,1240)_*		Post hoc
Pre-pandemic School Services, total services [range 0–7]	2.3 (2)	1.9 (1.8)	1.5 (1.5)	3.4 (1.8)	5.7 (1.4)	196.21	0.322	S4 > S3 > S1 > S2
Pre-pandemic Outside of School Services, total services [range 0–8]	2 (2)	1.8 (1.8)	1.5 (1.6)	2.4 (1.9)	6.4 (1.6)	195.06	0.321	S4 > S3 > S1 > S2
Stringency Index, raw score [range 0–100]	62.8 (12)	62.8 (12.3)	61.5 (11.5)	66.8 (12.5)	59 (9.2)	16.19	0.038	S3 > (S1 = S2 = S4)
Lifestyle Stress, raw score [range 2–10]	5.7 (2.3)	6.7 (2.3)	5.3 (2.2)	5.5 (2.2)	6.2 (2.4)	25.95	0.059	S1 = S4 > (S3 = S2)
COVID-19 Worries, raw score [range 4–20]	8.5 (3.6)	9.9 (4.2)	8.1 (3.3)	8.3 (3.2)	7.9 (4)	18.03	0.042	S1 > (S3 = S2 = S4)
New COVID-19 infections, new cases/day [range 0–8460]	446.7 (1009.9)	364.1 (880.4)	396.6 (989.2)	723 (1215.5)	104.4 (199.6)	11.28	0.027	S3 > (S2 = S1 = S4)
Child age, years [range 5–21]	11 (3.6)	10.8 (3.6)	11.6 (3.5)	10.2 (3.4)	9.7 (3.1)	15.84	0.037	S2 > (S1 > S4 = S3)

Random Forest top-ranked predictors are shown importance in descendent order. Abbreviations: S1, Broad symptom worsening only subgroup; S2, Average symptom/service changes subgroup; S3, Primarily modified services subgroup; S4, Primarily lost services subgroup; *; all tests reached statistical significance at *P* values < 0.001 after adjusting for FDR-correction); ${\eta }_{p}^{2}$, eta squared effect size

Notably, the predictor ranked eighth—i.e., pre-pandemic global severity—negligibly contributed to the classification model. Nevertheless, for illustrative and interpretation purposes, it is plotted in Fig. 6b and its descriptive statistics for each of the subgroups are summarized in Table 3. Similar to intellectual functioning, the four outcome subgroups did not differ with respect to pre-pandemic global severity except for the *primarily lost services* subgroup which was significantly more severe than the other three subgroups. Given that the *primarily lost services* subgroup was characterized by significantly greater intellectual impairment than the other three, we repeated supplementary RF analyses including intellectual categories in the subset of youth with intellectual functioning category available (*n* = 926). As detailed in Supplementary Results in Additional file 1, results were virtually unchanged relative to those from primary analyses; the feature indexing intellectual functioning had negligible predictive value for subgroup membership.

## Discussion

Prior disaster research in the general population, including studies of the COVID-19 pandemic [25, 66, 67], has consistently highlighted heterogeneity of mental health outcomes. The present study extends this insight into a multinational large sample of ASD/NDD youth by concomitantly assessing variability of changes in symptoms and therapeutic service access over the early stage of the pandemic. Across contributing samples, data-driven analyses identified four ASD/NDD subgroups: *broad symptom worsening only* (20%), *primarily modified services* (23%), *primarily lost services (*6%), and *average symptom/service changes* (53%)*.* Their profiles revealed that symptom and service changes have distinct patterns of covariation among youth with ASD/NDD. The subgroup with notable clinical worsening neither had the greatest number of services lost nor the greatest number of continued services, suggesting that other contributors to clinical worsening also exist. Conversely, youth with relatively stable symptoms were parsed into three subgroups that differed in service access changes going from pre-pandemic to pandemic times. Recognizing these subgroups elucidated unique effects of a set of predictors and highlighted different pathways to either stable or worsening clinical presentations. Indeed, the varying pandemic impact on symptoms in ASD/NDD youth was predicted by unique combinations of universal and ASD/NDD-related pre- and pandemic contexts in which service changes occur, rather than any one characteristic of the child, their family, or their environment alone.

Our results underscore that solely focusing on group-level effects leads to an incomplete picture of the COVID-19 pandemic impact on ASD/NDD youth. At the whole-sample level, multivariate analyses revealed that only sleep problems significantly worsened going from pre-pandemic to pandemic times. While results for this group-level approach confirm earlier pandemic reports of increased sleep problems in ASD/NDD [30, 36, 68], they failed to recognize a more vulnerable subgroup (i.e.*, broad symptom worsening* *only* outcome subgroup). Indeed, cluster analysis revealed that 20% of the children worsened even more than their ASD/NDD peers. Significant worsening affected a broad range of symptoms, including sleep problems, and beyond—i.e., externalizing and internalizing symptoms, RRBs, and daily living skills. For the remaining participants, symptom changes from pre-pandemic to pandemic times were within the aggregate average picture—i.e., statistically significant increases in sleep problems with other symptom severity being relatively stable. Consistent with prior literature [26–28], most youth in our ASD/NDD sample experienced a variety of service disruptions across settings, resulting in loss or modification of services (e.g., telehealth). However, youth in our aggregate sample experienced notable variability in service access. The present study found that the association between service and symptom changes is complex. In fact, clustering individuals across both services and symptom changes allowed us to further parse the relatively clinically stable youth into three homogeneous subgroups differing by service access changes. This enabled a fine-grained identification of predictors of differing pandemic outcomes—i.e., distinct profiles of symptom and service access changes across the four subgroups.

In the present study, the most relevant predictors of outcome subgroup membership included factors shared with the general population [23, 69], as well as others. Specifically, elevated perception of COVID-related risk (indexed by the COVID-19 Worries factor) and pandemic-related lifestyle stressors (i.e., restrictions on leaving home, cancellations of important events) predicted broad and more severe symptom changes in youth with NDD/ASD (i.e., the *broad symptom worsening* *only* outcome subgroup). Beyond these universal stressors, the present work identified age and a set of pandemic-related and NDD/ASD-specific experiences and contexts that must be considered collectively to provide a meaningful picture of pandemic impact in NDD/ASD youth (i.e., number of services received prior to the pandemic, COVID-19 containment measures, and COVID-19 new rates). These findings indicate that, in middle childhood, a greater number of baseline services may foster resilience and be protective to broad symptom worsening during a disaster, at least over its initial phase. Additionally, for those in middle childhood and living in areas with greater restrictions at times of high COVID-19 rates, continuing services, even if in modified format, may lead to a relatively more stable clinical profile.

Of note, pre-pandemic global clinical severity and other features related to the youth clinical presentation, including intellectual skills, negligibly contributed to prediction. Most children across subgroups had equivalent baseline symptom severity and average intelligence, except for the *primarily lost services* subgroup which was characterized by greater pre-pandemic impairment. These findings are in contrast with earlier ASD studies [28, 32] suggesting that preexisting challenges or symptom severity are associated with greater impairment during the pandemic. In considering possible explanations, we note that unlike the present efforts, prior NDD/ASD work focused on samples from relatively narrow geographical areas with largely similar COVID-19 rates and institutional containment responses. Additionally, inter-study differences in sample severity may exist; future studies focusing on a wider range of severity ratings are needed to build upon the present findings. Finally, neither specific NDD diagnosis nor psychiatric comorbidity’s burden robustly contributed to prediction of outcome subgroups. Thus, although our sample included predominantly autistic youth, considering the breadth of clinical impairment typically observed in ASD, our results suggest that the insights gained from the present effort can inform other NDD more broadly.

### Limitations

Although this is the most comprehensive and systematic study of the covariation of symptom and service changes in youth with NDD/ASD during the pandemic, results should be interpreted in light of several limitations. First, considering time constraints on questionnaire completion, albeit comprehensive, AFAR could not assess all possible domains of impact and/or prediction. For example, symptoms least expected to change over a short period of time, were given lower priority, most notably, social communication impairments [70]. Given the protracted nature of the pandemic, future studies should include assessment of longer-term changes in social communication skills. Similarly, although family demographics, parent education, and parent being an essential worker were assessed and included in our predictive model, parent’s mental health, recently reported to relate to children’s outcomes during the pandemic [71–74], was not assessed. Second, because of the urgency of the pandemic, we did not systematically involve other stakeholders in the survey adaptation process. However, the input of caregivers of children with ASD was accounted for in a number of informal ways during and at completion of the survey. Third, although the aggregate sample included youths with clinician-based diagnoses, previously collected measures of severity varied by contributing sample, and assessments of the role of *prior* severity were based on parent responses in the AFAR survey. Nevertheless, we found that the AFAR baseline global severity scores correlated with available standardized measures. Fourth, although relatively large, our aggregate convenience sample could not address all demographics. For example, females were underrepresented, and preschoolers were not included. Although consistent with a systematic review of pandemic studies in youth [4] sex at birth was not a robust predictor of impact, given recent reports of a male to female ratio of 3:1 in ASD [64], future studies should oversample females. Fifth, the present study focused on impact over the first six months of the pandemic using a cross-sectional design. Longitudinal coordinated study designs and infrastructures (e.g., common assessment measures) are needed to capture long-term outcomes and define stability of the subgroups over time. Finally, this first study relied exclusively on parent reports in order to capture the acute phase of the pandemic across a widest possible range of ASD/NDD youth and abilities using the same approach. Future studies should include self-report and/or direct observation to complement the parent’s perspective.

## Conclusions

As in the general population, the COVID-19 pandemic impact varies across ASD/NDD youth. Risk and resilience are rooted in the pre- and pandemic contexts in which service disruptions occur. Provision of mental healthcare in preparation for and during disasters are critical for ASD/NDD youth, further motivating efforts assessing effectiveness for telehealth and/or hybrid treatment programs. As heightened perception of risk was among the predictors for broad symptom worsening, during disasters, special attention should be paid to how much youth are concerned about a current crisis, and guided access to clear and appropriately dosed information is needed. Finally, this study demonstrates the value of international data sharing and collaborations. It also underscores the need for increased global coordination to include common assessment protocols and data structures to facilitate data sharing and analysis aimed to more readily assess and address the needs of those most vulnerable.

## Supplementary Information

**Additional file 1**. Contains the supplementary methods and results text, supplementary tables S1-S8 and supplementary figures S1-S4.

## Data Availability

Data were subjected to third-party restrictions. The codes used for EFA/CFA, RF, and HC are available at github.com/ChildMindInstitute/CRISIS-AFAR-analyses.

## Abbreviations

Activ-Inatt.: Activity Inattention
Adaptive Liv.: Adaptive Living skills
ADHD: Attention-deficit/hyperactivity disorder
AFAR: Adapted For Autism and Related neurodevelopmental conditions
ANOVA: Analysis of variance
ASD: Autism spectrum disorder
CADB: Center for Autism and Developing Brain, Weill Cornell Medical College/New York Presbyterian Hospital
CFA: Confirmatory factor analysis
CMI-AC: Child Mind Institute-Autism Center
CMI-HBN: CMI Healthy Brain Network
COVID-19: Coronavirus disease 2019
CRISIS: Coronavirus Health and Impact Survey Initiative
DSM: Diagnostic statistical manual of mental disorders
EFA: Exploratory factor analysis
FDR: False discovery rate
FIQ: Full intelligence quotient
HC: Hierarchical clustering
ICD-10: International Classification of Diseases
ID: Intellectual disability
MANCOVA: Multivariate analysis of covariance
Mod.: Modified
NDD: Neurodevelopmental disorders
OOBE: Out-of-bag error
Out.: Outside school
POND-CMH: Province of Ontario neurodevelopmental network, COVID mental health collaboration
RF: Random forest
RRB: Restricted and repetitive behaviors
Sleep Prob.: Sleep problems
SMF: Stella Maris Foundation, University of Pisa
TC: Thompson Center
TCD: Trinity College Dublin
UAth: University of Athens, National & Kapodistrian University of Athens, School of Medicine, First Department of Pediatrics, Unit of Developmental and Behavioral Pediatrics. “Aghia Sophia” Children’s Hospital
UBA: University Bari, Child Neuropsychiatry Unit, Policlinic of Bari
UCA: University of Cagliari, Child & Adolescent Neuropsychiatry Unit, A.Cao Paediatric Hospital
UCSF: University of California San Francisco
UFII: University of Naples Federico II, Child and Adolescent Neuropsychiatry Unit
UONPI-LO: Unita' Operativa di Neuropsichiatria dell’ Infanzia e dell' adolescenza, Lodi
USS: University of Sassari, Child Neuropsychiatry Unit, Azienda Ospedaliero-Universitaria
UTV: University Tor Vergata
