# Supplementary material for: CRISIS AFAR: an international collaborative study of the impact of the COVID-19 pandemic on mental health and service access in youth with autism and neurodevelopmental conditions
Source: Mol Autism. 2023 Feb 14;14:7. doi: 10.1186/s13229-022-00536-z (PMC9928142; doi:10.1186/s13229-022-00536-z)
Supplement: Supplementary file 1 — Additional file 1. Contains the supplementary methods and results text, supplementary tables S1-S8 and supplementary figures S1-S4. [file 13229_2022_536_MOESM1_ESM.docx]

**Supplementary Material**

**CRISIS AFAR: An International Collaborative Study of the Impact of the COVID-19 Pandemic on Mental Health and Service Access in Youth with Autism and Neurodevelopmental Conditions.**

Bethany Vibert**^*^**, Patricia Segura**^*^**, Louise Gallagher, Stelios Georgiades, Panagiota Pervanidou,  Audrey Thurm, Lindsay Alexander, Evdokia Anagnostou, Yuta Aoki, Catherine S. Birken, Somer L. Bishop, Jessica Boi, Carmela Bravaccio, Helena Brentani, Paola Canevini, Alessandra Carta, Alice Charach, Antonella Costantino, Katherine T. Cost, Elaine Andrade Cravo, Jennifer Crosbie, Chiara Davico, Federica Donno, Junya Fujino, Alessandra Gabellone, Cristiane Tezzari Geyer, Tomoya Hirota, Stephen Kanne, Makiko Kawashima, Elizabeth Kelley, Hosanna Kim, Young Shin Kim,  So Hyun Kim, Daphne J. Korczak, Meng-Chuan Lai, Lucia Margari, Gabriele Masi, Lucia Marzulli, Luigi Mazzone, Jane McGrath, Suneeta Monga, Paola Morosini, Shinichiro Nakajima, Antonio Narzisi,  Rob Nicolson, Aki Nikolaidis, Yoshihiro Noda, Kerri Nowell, Miriam Polizzi, Joana Portolese, Maria Pia Riccio, Manabu Saito, Ida Schwartz, Anish K. Simhal, Martina Siracusano, Stefano Sotgiu, Jacob Stroud, Fernando Sumiya, Yoshiyuki Tachibana, Nicole Takahashi, Riina Takahashi, Hiroki Tamon, Raffaella Tancredi, Benedetto Vitiello, Alessandro Zuddas^†^, Bennett Leventhal , Kathleen Merikangas, Michael P Milham, Adriana Di Martino.

****Contributed equally as co-first authors***

**† *Author deceased***

**This PDF file includes:**

1. Supplementary Methods

2. Supplementary Results

3. Supplementary Tables (S1 – S8)

4. Supplementary Figures (S1 – S4)

**Abbreviation List:**

ABAS-III = Adaptive Behavior Assessment Scale, Third Edition

ADHD = attention-deficit/hyperactivity disorder

ADI-R = Autism Diagnostic Interview-Revised

ADOS = Autism Diagnostic Observation Schedule

ADOS-EL = ADOS Expressive Language

AFAR = Adaptation For Autism and Related neurodevelopmental conditions

ANCOVA = analysis of covariance

ASD = autism spectrum disorder

CBCL = Child Behavior Checklist

CFA = confirmatory factor analysis

CFI = Bentler’s comparative fit index

COVID-19 = Coronavirus disease 2019

CRISIS = Coronavirus Health and Impact Survey Initiative

DQ = Developmental Quotient

DSM = Diagnostic Statistical Manual of Mental Disorders

EFA = exploratory factor analysis

FIQ = Full Scale Intelligence Quotient

GS = government stringency index

HBN = Healthy Brain Network

HC = hierarchical clustering

ICD-10 = International Classification of Diseases, 10^th^ edition

ID=Intellectual Disability

IQ = Intelligence Quotient

K-SADS-PL = Kiddie Schedule for Affective Disorders and Schizophrenia-Present and Lifetime

LD/NDD = language/learning or other neurodevelopmental disorders

MANCOVA = multivariate analysis of covariance

NDD = neurodevelopmental disorder

NVIQ = Nonverbal Intelligence Quotient

OOBE = out of bag error

POND-CMH = Province of Ontario Neurodevelopmental Disorders Network, COVID Mental Health collaboration

RF = random forest

RMSEA = root-mean-square error of approximation

RRB = restricted and repetitive behaviors

TCD = Trinity College Dublin

TLI = Tucker-Lewis index

VABS-3 = Vineland Adaptive Behavior Scales, Third Edition

VIQ = Verbal Intelligence Quotient

1. **Supplementary Methods**

**1.1. CRISIS Adaptation process to yield AFAR**

***1.1.1. Overview***

The Coronavirus Health and Impact Survey Initiative (CRISIS)^1^ adaptation for autism and related neurodevelopmental conditions (AFAR) was led by a working group of psychologists and psychiatrists with clinical and research expertise in autism spectrum disorder (ASD) and other neurodevelopmental disorders (NDDs) (A.D.M., L.G., S.G., P.P., A.T., B.V.). Their goal was to preserve the main structure of CRISIS^1^ developed for the general population while adding domains most relevant to ASD/NDD. To this end, first each member of the working adaptation group independently reviewed and coded each item of the CRISIS Parent/Caregiver Baseline 5-21 survey to be retained or removed, as well as noted rewording suggestions (see below 1.1.2). Second, the working group reached an agreement on the assessment domains to be newly included. Then, the working group, in consultation with another AFAR network member (S.H.K), assessed which items were theoretically applicable to children younger than five years. Later, minor rewording for some items were made based on feedback and agreement with the larger AFAR network. The final version 0.5.1 of AFAR Baseline Parent/Caregiver (3-21 years) consists of 96 total independent items, including 34 questions asked twice for *Prior* (i.e., three months prior to the start of the pandemic in the respondent’s geographical area) and *Current* time points (i.e., last two weeks). The 96 total items include 26 multiple choices, 60 Likert scale, four yes/no, four ‘fill-in-the-blank,’ and two open ended questions. AFAR is licensed on Creative Commons (CC) BY4.0 and available at http://www.crisissurvey.org/crisis-afar/. Overall, 47 questions were retained (34 unchanged and 13 slightly reworded), 17 new questions were added, 26 were removed from the CRISIS survey as summarized in Table 1 and detailed below.

***1.1.2. Retained/removed content***

Items with more than 90% agreement for retention or removal across the working group raters were modified accordingly. For the other items, the working group met to reach a consensus for retention or removal. As shown in Table 1, the original CRISIS domains retained included: Background, COVID-19 Health/Exposure Status, Life Changes, COVID-19 Worries, Daily Behaviors/Media). Among them, 47 questions/response options were retained (18 from Background, six from the COVID-19 Health/Exposure Status, seven from Life Changes, five from COVID-19 Worries, nine from Daily Behaviors/Media; and two open ended questions). Of them, 13 questions and/or response options were slightly reworded (five in Background, one in the COVID-19 Health/Exposure Status, five in Life Changes, one each in the COVID-19 Worries and Daily Behaviors/Media). Rewordings largely aimed to account for potential differences in verbal ability across individuals with ASD/NDD. For example, in one question within the COVID-19 Health/Exposure Status domain the original question asked, “How much is your child asking questions, reading, or talking about Coronavirus/COVID-19?” this question was rephrased to add watching content to read as follows “How much is your child asking questions, reading, watching content or talking about Coronavirus/COVID-19?”

With the addition of ASD/NDD relevant domains (see below 1.1.3), to contain the survey’s length, two domains and a few additional items were removed. Specifically, the Mood State domain was replaced by a newly added set of questions on cooccurring psychopathology focusing on observable behaviors rather than attempting to seek reports on internal states. The Substance Use domain was also removed given that it is more accurately measured by self-reports,^2,3^ which are beyond the scope of the present study. Additional nine items were removed (eight for life changes and one from Background) if they were only applicable to subset of individuals with verbal skills (e.g., “How many people, from outside your household has your child had an in-person conversation with?”), did not target observable behaviors (e.g., “How would you rate your child’s overall Mental/Emotional health before the Coronavirus/COVID-19 crisis in your area?”).

***1.1.3. Newly included domains/questions***

The working group agreed to include three new domains as relevant for ASD/NDD based on one prior disaster report^4^ available at the time of the CRISIS-AFAR development (April 2020) and the larger clinical literature.^5–9^ The new domains were Adaptive Living Skills, Restricted and Repetitive Interests and Behaviors (RRB), and Co-Occurring Psychopathology. Two sub teams of the working group drafted specific questions for the targeted domains which were then reviewed, agreed, and further edited in following videoconferences with all members of the group. This process led to including: 1) an Adaptive Living Skills domain of four items asking the degree of one child’s independence in playing, structuring their activities, organizing mealtimes, and participating in hygiene and daily living routines; 2) an RRB domain of six items capturing the frequency of lower- and higher-order RRB^10,11^ described in the DSM-5 diagnostic criteria^12^ (i.e. two items of sensorimotor RRB and four items assessing aspects of insistence on sameness). Lastly, to evaluate co-occurring psychopathology, caregivers were asked to indicate if specific externalizing and internalizing symptoms, often reported in children and adolescents with ASD/NDD,^5,7,13^ were present. If so, parents/caregivers were asked to indicate the symptom level of severity. Additionally, given the high prevalence of sleep problems in ASD,^14^ two questions on insomnia were added to the CRISIS sleep items included in the Daily Behavior and Media domain. Consistent with the CRISIS structure, these symptom domains were assessed at two time points: 1) over the three months prior to the COVID-19 pandemic beginning in the respondent's geographical area, and 2) over the past two weeks at time of data collection (hereafter referred to as *Prior* and *Current* time points, respectively). To evaluate changes in therapeutic services, the working group adapted and added to selected items from the Caring through COVID survey developed for individuals with syndromic intellectual disabilities.^15^ Specifically, caregivers were asked whether therapeutic services typically received at and outside school settings (e.g., speech language, physical and/or occupational therapy, social skills interventions) were lost or continued. If they were continued, caregivers were asked to specify if they had been received virtually (telehealth/email) or not and how helpful they were perceived by the caregivers. Caregivers were additionally asked about a range of medical services (e.g., gastroenterology needed and accessed), as well as if psychotropic medication prescription/monitoring were needed. Although caregivers/stakeholder focus groups were not systematically involved in this process, their input was informally included in several ways along the process. First, AFAR developers included information from clinical contacts with caregivers during the acute phases of the pandemic. Second, the service questions section adopted from the “Caring Through COVID” were previously piloted in children with NDD and distributed by an advocacy group.^13^ Third, at completion of the survey, several sites shared it with stakeholder/s caregivers who supported the survey for data collection. These included the national autism charity AsIAM in Ireland, and parents involved with the Province of Ontario Neurodevelopmental Disorders (POND-CMH) Network^16–18^.

***1.1.4. Age range applicability***

As shown in Fig. 1, all questions in the Co-occurring Problems domain, and two items of the Daily Behaviors and Media domain (i.e., interacting using technology to engage with peers and family) were deemed not applicable for children aged below five years. The group agreed that the remaining AFAR questions were developmentally appropriate for assessing children aged three years and above.

**1.2. Exploratory and confirmatory factor analyses (EFA, CFA)**

For factor analysis, we used Lavaan Package^19^ in R version 4.0.0.^20^ The R code used for analyses can be found at github.com/ChildMindInstitute/CRISIS-AFAR-analyses. Exploratory Factor Analyses (EFA) were conducted across all items in the Likert-scale based AFAR assessment domains. Only items with factor loadings > 0.3 in the EFA were included in subsequent confirmatory factor analyses (CFA). Other items were removed as needed, to reach a final factor structure composed of more than one item meeting at least two of four goodness-of-fit criteria, as well as theoretical plausibility. Goodness-of-fit criteria commonly used in the literature included χ2 significance (non-significant values suggest good of fit^21^), root-mean-square error of approximation (RMSEA; cutoffs of .01, .05, and .08 indicate excellent, good, and acceptable fit, respectively^22^); the Tucker-Lewis index^23^ (TLI; ≥.95 indicates good fitting^24^), and the Bentler’s comparative fit index^25^ (CFI; ≥.96 indicates goodness of fit). To explore replicability of findings, EFA and CFA were conducted in split-half datasets derived from the aggregate sample (i.e., derived by aggregating the 1275 datasets across contributing samples). Each of the datasets were group-matched by contributing sample, sex, child age, Full Intelligence Quotient (FIQ), and primary diagnosis (i.e., ASD, attention-deficit/hyperactivity disorder [ADHD] without ASD, Other NDD). Further, to assess stability of findings, for the domains designed to assess *Prior* and *Current* behaviors, EFA and CFA were conducted using the *Prior* scores, their stability was assessed via a CFA on items using the *Current* scores across the whole aggregate sample.

**1.3. AFAR data collection and selection**

***1.3.1. Sites/contributing samples***

As shown in Fig.1 and Table S1, data were collected in 14 clinical and/or research institutions across Europe and North America yielding 15 independent datasets, referred to as contributing samples. These included one sample collected across five centers in Ontario, Canada as part of a COVID-19 multi-network collaboration in pediatrics that included the Province of Ontario Neurodevelopmental Disorders (POND-CMH) Network,^16–18^ five samples collected in four research and clinical institutions across the United States of America (New York, California, and Missouri), and seven sample collected across Europe (Greece, Italy, and Ireland).

***1.3.2. Data collection protocols***

For each sample, AFAR data collection protocols are summarized in Table S1. Briefly, caregivers of children aged between 3-21 years who had previously received a clinician-based DSM-IV/5^26,27^ or ICD-10^28^ diagnosis of ASD and/or other NDD were invited via email and/or phone contacts to complete the AFAR survey. Initial contacts for this study included families who enrolled in ongoing research studies (three contributing samples), clinical services (eight samples), or both (four samples). Across all sites caregivers were invited to complete the survey regardless of their child’s biological sex, intellectual quotient (IQ), parent-reported race, ethnicity, or socioeconomic status. For all but one sample, surveys were administered exclusively online, and at least weekly reminders were used. No reward at completion was given for all except three contributing samples that provided $10 and $25 gift cards at completion (at Child Mind Institute and University of California, San Francisco, respectively). Data collection time and duration varied by sample over the period between April 24 and October 20, 2020 (Fig. 1, Table S1). Reflecting geographical variation across the collection sites, AFAR was completed under different COVID-19 related restrictions ranging from shelter-in-place ordered uniformly in each geographical area, to less stringent and geographically heterogenous restrictions. This allowed us to naturalistically explore the impact of distinct restrictions on the NDD/ASD impact subgroups identified with hierarchical clustering (see below on the Random Forest 1.8 section, and Fig. 2 for the feature selected for prediction). Data were organized in a common template.

**1.4. Prior diagnostic and clinical protocols**

To provide a more accurate clinical characterization of the aggregate dataset than otherwise feasible in large scale online survey efforts, the AFAR network aimed to collect data of children previously well characterized via systematic clinical assessment protocols conducted by clinicians prior to the COVID-19 pandemic. Although the diagnostic clinical protocols varied by contributing sample, within each site, clinician-based DSM-IV/5 or ICD-10 estimate diagnoses were previously reached based on parent interviews, direct child observations, and cognitive testing, review of parent questionnaires and other available records followed by group case conferences to reach best-estimate diagnoses following a review of observations and discussion of clinical impressions. Specifically, as summarized in Table S1, the gold-standard Autism Diagnostic Interview-Revised (ADI-R)^29^ and/or Autism Diagnostic Observation Schedule (ADOS, G and/or second editions)^30,31^ were systematically used in 12 of the 15 contributing samples and in subsets of children for the remaining two samples (Healthy Brain Network [HBN], Trinity College - Dublin [TCD]). In six samples, the Kiddie Schedule for Affective Disorders and Schizophrenia-Present and Lifetime (K-SADS-PL)^32^ or unstructured psychiatric interviews were used to aid diagnosis and for further clinical characterization. Adaptive functioning was systematically assessed via standardized interviews (e.g., Vineland-3 Comprehensive Parent Interview, Third Edition [VABS-3]^33^ or Adaptive Behavior Assessment Scale, Third Edition [ABAS-III]^34^) in all but two samples (TCD, University of Athens [UAth]). Standardized cognitive testing was administered to all children, as feasible, with the specific tests selected depending on the child’s age and developmental level. The clinical characteristics of each contributing sample and of the aggregate dataset are summarized in Fig. 3 and Table S2.

**1.5. Harmonization of shared phenotypic information**

***1.5.1. Overview***

Along with diagnostic labels (available for 100% of the aggregate dataset), whenever feasible, information on intellectual functioning and other quantitative metrics of symptom severity were also shared. These included data for intelligence (80% of the aggregate sample), a measure of expressive language derived from the ADOS-2^35^ (57%), psychopathology indexed by the parent-based Child Behavior Checklist (CBCL)^36^ summary T scores (59%), and adaptive functioning (53%). Below, we describe the curation and harmonization approach used for comparability across samples.

***1.5.2. Diagnostics***

Per study design, all contributing samples provided clinician-based diagnostic labels. They were consistent with the DSM-5 diagnostic criteria for all but two sites: UONPI-LO that used DSM-IV, and TCD that used ICD-10 for a subset of children. Diagnostic labels were mapped into the DSM-5 nomenclature (e.g., Asperger was remapped into ASD). When one child had multiple diagnostic labels, the most frequent diagnosis in the aggregate sample was considered the child’s primary diagnosis and the other diagnostic labels were accounted for as comorbidities (e.g., ASD with or without attention-deficit/hyperactivity disorder [ADHD] and/or anxiety). When a child did not have the most frequent diagnostic label, the second most frequent label in the aggregate sample was considered the child’s primary diagnosis - if present (e.g., ADHD without ASD - ADHD_w/oASD_) and other labels, if assigned to that child, were considered comorbidities (e.g., ADHD_w/oASD_ with comorbid anxiety and/or learning disorder). This process was repeated iteratively for the following most frequent diagnostic labels (e.g., intellectual disability without ASD and without ADHD). See Fig. 3 and Table S2.

In the aggregate dataset, ASD (with or without comorbid diagnoses) was the most frequent diagnostic label (n=1004, 79%). Of the remaining 271 children without ASD, the most frequent diagnostic criteria were ADHD (n=214, 80%), for intellectual disability (ID; n=15, 6%), anxiety disorders (n=10, 4%), obsessive compulsive disorder (n=9, 3%), disruptive-impulse control and genetic conditions (n=7, 3% for each diagnostic label), language/learning or other neurodevelopmental disorders (LD/NDD; n=5, 2%), depressive disorders (n=2, 1%), and n=1 each for tic and avoidant personality disorders. Among the children with ASD, 434 (43%) were reported to meet criteria for at least one co-occurring diagnosis. Of those with co-occurring diagnoses, 270 (62%) met criteria for ADHD, 116 (26%) for ID, 81 (19%) for an anxiety disorder, 77 (18%) for LD/NDD. Among those with ADHD_w/oASD_, most were without comorbidity (n=171, 80%). Similarly, among those with other diagnoses without neither ASD nor ADHD (n=57), the majority had no comorbid diagnoses (n=44, 77%). See Table S2 for diagnostic and other clinical information on the aggregate dataset and each contributing sample.

***1.5.3. Intelligence estimates***

Information about intellectual functioning was available for 80% (n=1014) of the aggregate dataset; it included either quantitative metrics or qualitative clinician-based categories of intelligence (n=962 and 47 children for n=15 and n=6 samples, respectively). For comparability across samples, since full intelligence quotient (FIQ) was the most represented metric in the aggregate dataset (60%, n=759 children), we focused on mapping quantitative variables of full-scale intelligence in a comparable distribution of Mean=100 and Standard Deviation=15. When only VIQ and NVIQ scores were provided (1% of the aggregate, n=15 children, three samples), their average was used to estimate FIQ. When quantitative metrics of development (e.g., obtained with Mullen Scales of Early Learning^37^) were provided (5% of the dataset, n=63 children, eight samples), they were converted to Developmental Quotients (DQ), as in prior literature in NDD/ASD.^7-38^ For those samples with less than 15% FIQ data missing, within each sample, we imputed missing data using the average FIQ computed across children with non-missing data group-matched by age and diagnosis (n=54 children, 4%, six samples).

Taken together, this process resulted in a total of n=891 (70% of the aggregate) children with a quantitative FIQ (or equivalent) available and n=47 (4% of the aggregate) with a qualitative clinician-based rating of intellectual functioning. To harmonize qualitative and quantitative intelligence estimates of full-scale intelligence, we mapped all quantitative scores onto qualitative categories as described above. Then, to harmonize quantitative and qualitative estimates of full-scale intelligence, FIQ scores were mapped onto qualitative categories ranging from *above average* to *profound intellectual functioning*. Specifically, children with FIQ scores within or above one standard deviation of the standard mean were labeled with Average (FIQ=85-114); or Above Average intelligence (FIQ >115). Those with FIQ estimates below 85 were categorized as Borderline intelligence (84-71), Mild (51-70), Moderate (FIQ:35-50), Severe (FIQ: 21-34), and Profound intellectual disability (FIQ<20).^26^ As a result of the harmonization step, the total number of children with FIQ estimates was n=938 (74% of the aggregate) across all 15 contributing samples (Fig. 3). Of the remaining n=337 (26%) without any FIQ equivalent available, n=117 children had NVIQ scores (9%, 7 samples) and n=220 had no available quantitative FIQ, NVIQ, or VIQ scores available. NVIQ scores were not converted to qualitative full-scale estimates, but they are described in Table S2.

***1.5.4. Expressive language***

Measures of language were not uniformly administered across sites, nor shared across all samples. Thus, to obtain a comparable characterization of verbal skills, we leveraged previously administered ADOS to compute a measure of expressive language derived by one ADOS item that has been validated in prior work^35^ (i.e., ADOS-EL). This was computed and shared for 57% of the aggregate dataset (n=730 children, 13 samples). As a note, ADOS calibrated severity scores^39^ were shared only for 38% of the aggregate (n=479 children; seven samples). As such, we did not use them in targeted analyses but, considering their common use in ASD studies, we report their descriptive statistics in Table S2.

***1.5.5. Adaptive functioning***

Quantitative indices of adaptive functioning were shared for nine contributing samples yielding data for 53% of the aggregate dataset (n= 674). Different editions of the VABS^33,40^ were used across six samples, the remaining three samples used the ABAS-3. Both instruments assess functioning across multiple living domains and provide a summary score, Adaptive Behavior Composite and General Adaptive Composite, respectively, in a standard scale with M=100 and SD=1). Thus, for comparability across samples, composite scores were used to characterize global adaptive functioning.

**1.6. Pandemic Impact on Overall Sample**

Although the main goal of the present study was to assess whether different COVID-19 pandemic impact subgroups exist in ASD/NDD, for interpretation of results and consistency with the pandemic literature more broadly, we examined symptom and service access changes across the aggregate sample (Table S7 and Fig. 5). Accordingly, to explore symptom changes between *Prior* and *Current* scores for the seven symptom domains analyzed in clustering analysis, we conducted a one-way repeated measures MANCOVA using time as within-subject factor (*Prior* versus *Current)*, including contributing sample as covariate across the same seven symptom domains analyzed with clustering. Following findings of a statistically significant main effect of time, post-hoc univariate one-way repeated measure ANCOVAs were performed to compare the two Prior vs. Current timepoints within each symptom domain. All tests were corrected with False Discovery Rate (FDR) at q<0.05. To characterize service changes across the aggregate sample, we used measures of central tendency (e.g., mean for continuous variables, proportions for categorical variables) for the number of services whose access was lost and/or continued at school and outside school, separately.

**1.7. Hierarchical Clustering (HC)**

To examine whether homogeneous subgroups of ASD/NDD children with distinct profiles of change in symptoms and/or service access exist, we performed agglomerative hierarchical clustering (*hclust* function from the *cluster* package in R version 3.6.1).^41^ We used Euclidean distance (*dist* R function) and Ward's minimum variance method (*ward.D2* option; Murtagh and Legendre’s criterion).^42^ Clustering was conducted across 11 features of impact based on AFAR. Seven of the features were comprised of change scores indexed by difference scores between *Current* (“last 2 weeks”) and *Prior* (“three months prior to COVID-19”) time points in the seven domains identified by factor analyses (i.e., Adaptive Living Skills, High- and Low-order RRB, Anxiety, Oppositional Behavior, Sleep Problems, Activity/Attention). The remaining four features included the total number of therapeutic services that were lost or continued in and outside school following the pandemic. All scores were converted to standard z scores prior to clustering. The *NbClust* package,^43^ a consensus-based algorithm that ranks solutions based on the degree of agreement across 30 different established clustering-quality indices (e.g., Davies–Bouldin or Gap statistics) was used to determine the optimal number of clusters between 2 and 15 solutions according to a majority rule. The implementation and R code used for the analysis can be found at github.com/ChildMindInstitute/CRISIS-AFAR-analyses.

**1.8. Random forest (RF) feature selection and model**

To assess the relative importance of a set of COVID-19 pandemic-related feature, socio-demographic and child clinical variables in predicting the COVID-19 impact subgroups, we used RF implemented in scikit-learn^44^ (see GitHub webpage).^45^

***1.8.1. RF analytical framework***

Consistent with prior work,^46^ we assessed each feature importance across 4000 bootstrap samples that were drawn with replacement from the children in the aggregate dataset with the variables included in the RF available (N=1244; see Fig. 2). Each bootstrap sample was split in a training (2/3) and a testing (1/3) set. For each sample, a classification model for four subgroups was first developed by growing 300 trees in the training set and then applied to the test set. Feature importance was calculated using the permutation importance method^47^ similarly to prior work. ^46,48^ Briefly, within each sample, we first recorded a baseline accuracy score for the trained model, permuted the values of each feature, then passed the test sample back through the RF and recomputed accuracy. The importance of a given feature was indexed by the difference between the baseline and the new accuracy value obtained across permutations; this is known as the ‘out of bag error’ (OOBE). An average OOBE value was calculated for each of the 20 analyzed features. The larger the OOBE value, the more important the feature is. All features were ranked accordingly in decreasing order of importance.

***1.8.2. RF feature selection***

As shown in Fig. 2, the RF model assessed 20 features indexing family and child socio-demographics, their pandemic experience, pre-pandemic child clinical characteristics including services previously received, baseline symptom severity diagnostic status (ASD vs other non-ASD NDD) and number of comorbid diagnoses, as well as different COVID-19 pandemic context indices such as government response, new infection rates, and time since the start of the pandemic. Most of these features were largely derived from AFAR responses and prior characterization; COVID-19 rates at the time of completion in the respondent’s geographical area and the Government stringency index (see below) were derived from open data sources^49–51^ (see below).

The child/family socio-demographics, pandemic impact on jobs and health, child’s COVID-19 worries, and perceived economic and life stress were based on AFAR factors identified in EFA/CFA and other responses on the AFAR background section. For the pre-pandemic clinical presentations, we computed a baseline (i.e., three months prior to the pandemic) global severity score across the seven clinical factors of AFAR (indexed as an average z score). We also used primary diagnosis of ASD versus non-ASD and number of comorbid diagnoses. For this primary RF model, we did not include standardized clinical measures collected prior to the pandemic described above (i.e., CBCL, VABS/ABAS, ADOS-EL) as they were available for less than 70% of the aggregate individually and when combined only for n=453 individuals (36% of the aggregate). However, as shown in Figure S4, these clinical measures were statistically significantly correlated with the Baseline Global Severity from AFAR. Nevertheless, we repeated RF models including these standardized variables in the smaller sample with all variables examined and the pattern of results remained the same (data not shown).

To quantify government responses to the COVID-19 pandemic, for each child at the time of the AFAR data collection in their geographical area, we used the government stringency (GS) index computed by the Oxford’s Coronavirus Government Response Tracker^49^ (latest download on 25 August 2021). The GS index combines metrics of infection containment and public information campaigns, it ranges from 0 to 100, higher scores reflect stricter government policies, and it is provided by day in each territory. For each child, we selected the GS index computed at the same date of the AFAR completion in the respondent’s living area. GS were available at the subnational level for USA and Canada (e.g., New York, California, Missouri, and Ontario) and at the nation level for Greece, Italy, and Ireland.

To quantify new infection rates in a given child’s geographical area at the time of AFAR data collection, we used the publicly available Our World in Data's (OWID) COVID-19 tracker.^50^ Data on new COVID-19 infected cases were available on a daily basis for the United States and Canada at a sub-national level. Since this data source only had country-level information across Europe, for the European samples we opted to use the COVID-19 European regional tracker^51^ which provides daily new infection rates at the regional level. Based on each European contributing sample’s catchment areas, we retrieved location-specific information. For example, for children in TCD we used the Eastern-Midland region of Dublin. Regarding the sample collected by UAth, since over the time interval during which AFAR data were collected in the Attica region, major data gaps existed, we used country-level infection rate data available in the OWID that were available for the same period.

1. **Supplementary Results**

**2.1. AFAR data collected**

As shown in Figure S1, of the total of 4458 surveys known to be sent to families, 1595 (36%) datasets were returned; success rate varied by contributing sample (13% to 84%; median=40% SD=22%). Six contributing samples had a return rate >50%; they included sites located in the USA, Canada, and Italy, and totaled 724 individuals. Nine contributing samples had return rates <50%; they included sites located in the USA, Italy, Greece, and Ireland; they totaled 551 individuals. As indicated below, secondary hierarchical clustering conducted on contributing sample sets defined by differences in return rates (> or < 50%) showed similar patterns as those observed in primary analyses.

**2.2 Factor Structure**

As shown in Table S5, following EFA three items were excluded from further analyses due to factor loading <0.3. Similarly, two items were not included in CFA as they resulted as single item factors in EFA; one was “*worry about own mental health*” in the COVID-19 Worry domain, the other was “*positive changes,*” in the Life Change domain. Finally, one item “*deliberately injuring self*” loaded in the attention/activity factor was removed for interpretability. As summarized in Table S6, following these steps CFA conducted on the *Prior* scores of split-half samples yielded a single Adaptive Living Skills factor, two RRB-related factors (High- and Lower-order),^10,11^ and four Co-occurring Problem Behavior factors (i.e., Anxiety/Affect, Oppositional Behavior, Sleep Problems, Activity/Attention. As in the general population,^1^ COVID-19 Worries yielded a single factor, while Daily Behaviors/Media and Life Changes yielded multiple factors (five and two, respectively). Results remained consistent in secondary CFA across the entire sample on *Current* ratings (see Table S6).

**2.3. Proportion of children with service changes**.

Across the aggregate sample, in terms of proportion of children affected by service changes, among those previously receiving services (n=1008 and n=939, at school and outside school, respectively), 61% and 56% lost at least one service; with 37% and 42% lost all prior services within and outside school, respectively, while 63% and 58% had at least one service modified within and outside school; with 39% and 36% of the sample having all their prior services modified.

**2.4. Post-Hoc analyses on subgroup**

***2.4.1. Impact subgroups comparisons relative to AFAR symptom changes****.*

One-way MANOVA analyses revealed a significant main effect of subgroups across the seven symptom domains examined (F_(3,1271)_=37.0, p<0.001, Pillai’s Trace=0.51, eta squared=0.17). Post Hoc one-way ANOVAs, followed by Tukey Pairwise group mean comparisons assessed differences across symptom change domains by subgroups (FDR correction q<0.05; Table S7). Specifically, the *broad symptom worsening only* subgroup showed significantly higher positive difference scores (i.e., worsening) in all domains relative to the other subgroups. The remaining three subgroups did not statistically differ from each other in regard to symptom domain except for the Anxiety and Oppositional factor scores which worsened in the *primarily modified services*, relative to the *average symptom/service changes* and *primarily lost services* subgroups (Table S7). Given potential differences between contributing samples, to examine if the pattern of the impact subgroup differences was confounded by contributing sample, we conducted a one-way MANCOVA including contributing sample as covariates (dummy variable 1 to 15). The pattern of subgroup differences remained unchanged (F_(3,1257)_=36.8, p <0.001, Pillai’s Trace=.51, eta squared=0.17) and no statistically significant main effect of contributing sample (p=0.2) on symptom change scores was detected.

***2.4.2. Impact subgroups comparisons relative to AFAR service changes***

Following a statistically significant one-way MANOVA the average number of services lost or continued at each setting- e.g., at school and outside school; (F_(3,1271)_= 149.5, p<0.001, Pillai’s Trace= .96, eta squared=0.32), Post Hoc one-way ANOVAs, followed by Tukey Pairwise group mean comparisons assessed service changes (FDR correction p<0.05; Table S7). Regarding services lost, the *primarily lost services* subgroup had the greatest number of services lost (M+SD across both settings: 5.7+1.6) which was significantly higher relative to all other Subgroups. The *primarily modified* *services* subgroup had the lowest number of services lost (M+SD across all settings: 0.5+0.9). This was statistically different relative to both the *broad symptom worsening only* and *average symptom/service changes* subgroups (M+SD: 1.0+1.4 and 1.4+1.8, respectively), which, in turn, did not statistically differ from each other. For modified services, the *primarily modified services* subgroup had the highest and statistically different number of modified services (M+SD across all settings: 3+1) relative to the other three subgroups, which, in turn, did not statistically differ from each other (M+SD range: 0.3-0.7 + 0.6-0.8). Given potential differences between contributing samples, to examine if the pattern of service differences was confounded by the contributing sample, a one-way MANCOVA including sample as a covariate (dummy variable 1 to 15) was repeated. The pattern of difference remained significant (F_(3,1257_)=157.3, p <0.001, Pillai’s Trace=1, eta squared=0.3). The effect of the contributing sample was statistically significant (p< 0.001); as such we followed up with ANCOVA comparisons; the pattern of results remained largely consistent to what reported above (data not shown).

**2.5. Hierarchical Clustering (HC) follow-up analyses**

To assess that the primary results were not affected by differences seen in return rates across contributing samples (Fig. S1), we repeated these clustering analyses separately on subsets of data derived across contributing samples with return rates above or below 50%. Specifically, n=724 (57% of the aggregate sample) data were included in the six contributing samples with return rates > 50% while n=551 (43% of the aggregate) were derived from the nine contributing samples with return rates <50%. For each subsample, the pattern of results remained virtually unchanged from that obtained in primary HC analyses (data not shown).

Supplementary Table 8 summarizes, for each subgroup, key demographics (age and sex at birth) and intellectual functioning indexed by the intelligence qualitative categories defined in 1.5.3. The groups did not differ in respect to the distribution of sex at birth; they did differ for age and intellectual functioning. Regarding age, following the statistically significant results of the ANOVA, Post Hoc pairwise subgroup comparisons indicated that the subgroup a*verage symptom/service change* was significantly older (11.6 + 3.5 years) than the other three subgroups. Youth in the three subgroups were overall in mid-childhood (10.8 + 3.6, 10.2 + 3.4, 9.7*+*3.1, for the *broad symptom worsening*, *primarily modified services*, *primarily lost services* subgroups, respectively) with the subgroup *primarily lost services* being significantly younger relative to the *broad symptom* worsening but not to the *primarily modified service* subgroups*.* Most children across the four outcome subgroups fell in the average/above average intelligence categories except for those in the *primarily lost services s*ubgroup. This subgroup was characterized by significantly greater rates of moderate and severe intelligence disability categories compared to the other subgroups.

**Secondary Random Forest analyses**

Our primary random forest analyses did not include intellectual ability as data was available for a subset (n=926) of the sample examined in RF (n=1244). Thus, given that in-between mean subgroup comparisons revealed that the outcome subgroups significantly differed in respect o intelligence category, we repeated RF analyses adding intellectual functioning to the 20 features assessed in primary analyses using the n=926 sample with the available intellectual category data. Results were virtually unchanged from those in primary analyses. Specifically, the RF classification model including 21 features, predicted subgroup membership with 82% accuracy (precision/sensitivity=84%, recall/specificity=76%). The predictor ranking remained virtually unchanged. Intellectual functioning category was ranked 21^st^ in feature importance and had OOBE<1%. Thus, it has a negligible role in the prediction of subgroup membership.

1. **Supplementary Tables**

**Table S1. Data collection and associated pre-pandemic phenotyping protocols for the 15 contributing samples.**

|  | **Contributing Sample Label** | **Contact Source** | **AFAR survey version/ administration mode** | **Catchment area** | **Data collection interval (d/m/2020)^a^** | **COVID 19 response at the time of collection** | **Target age range (years)** | **Parent Interviews** | **ADOS** | **Child Cognitive Testing** |
| --- | --- | --- | --- | --- | --- | --- | --- | --- | --- | --- |
| **USA, New York State** | CADB | C, R | E 0.5.1; ReDCap | Tri-state New York Metropolitan area | 06/18-08/15 | 03/28-06/07: Shelter-in-place order for 100% of non-essential workforce;  06/08 - 07/19: Gradual reopening from phase 1- phase 4 (e.g., malls, zoos, and gardens allowed to reopen, indoor dining at limited capacities) | 3-18 | ADI-R, VABS-II/3 | y | Bayley; DAS-II, MSEL; WASI; Ravens |
|  | CMI-AC | R | E 0.4.0, 0.5.1^b^; ReDCap |  | 04/24-06/02^a^ |  | 3-12 | ADI-R (<5 years); ASI and KSADS-PL (>5 years), VABS-II/3 | y | DAS-II, MSEL |
|  | CMI-HBN | R | E 0.5.1; ReDCap |  | 05/21-07/01 |  | 5-21 | KSADS-PL; ADI-R^c^, VABS-II | subset only | KBIT-2; WISC-V; WAIS; WASI |
| **USA, California** | UCSF | C | E 0.4.0; ReDCap | San Francisco Bay area | 06/12-10/19^a^ | 03/16-05/14: Shelter-in-place order for 100% of non-essential workforce;  05/15: Gradual reopening | 3-18 | ADI-R, VABS-II/3 | y | DAS-II; WAIS-IV; WISC-IV; WAIS-IV; MSEL; Ravens-2; WPPSI-IV |

|  | **Contributing Sample Label** | **Contact Source** | **AFAR survey version/ administration mode** | **Catchment area** | **Data collection interval (d/m/2020)**^a^ | **COVID 19 response at the time of collection** | **Target age range (years)** | **Parent Interviews** | **ADOS** | **Child Cognitive Testing** |
| --- | --- | --- | --- | --- | --- | --- | --- | --- | --- | --- |
| **USA, Missouri** | TC | C | E 0.4.0; ReDCap | Missouri, Kansas Illinois | 06/11-10/20^a^ | No state-directed response specific to COVID-19; decisions about lockdowns and other restrictions/mandates varied considerably by city and county | 3-17 | ABAS | y | DAS-II; KBIT-2; WISC-IV/V; WAIS; WPPSI-IV |
| **Canada, Ontario** | POND-CMH | R | E 0.4.0; RedCap | South Ontario (Toronto, Hamilton, London, Kingston areas) | 5/14-8/27^a^ | 3/17-8/15 Lock down order for 100% non-essential workers | 3-21 | ADI-R, ABAS, K-SADS, PICS | y | WASI or other Wechsler tests, Standford Binet, Mullen |
| **Greece** | UAth | C | G 0.4.0, Google Forms; phone interviews | Athens metropolitan area (~75%) and west/central Greece + Islands (25%). | 05/06-06/25 | 03/11-5/15: Lockdown order for 100% of non-essential workforce followed by a gradual re-opening of schools and special services | 3-18 | ADI-R | y | Ravens; WISC-III |
| **Ireland** | TCD | C, R, National Advocacy^d^ | E 0.4.0^e^; Qualtrics | Dublin Metropolitan area, East Ireland | 06/09-08/17 | 03/20-05/20: Lockdown for 100% of non-essential workforce  06/08 -08/31: Gradual reopening | 3-20 | ADI-R^c^, VABS-II^c^, semi-structured psychiatric interviews | subset only | WISC |

|  | **Contributing Sample Label** | **Contact Source** | **AFAR survey version/ administration mode** | **Catchment area** | **Data collection interval (d/m/2020)**^a^ | **COVID 19 response at the time of collection** | **Target age range (years)** | **Parent Interviews** | **ADOS** | **Child Cognitive Testing** |
| --- | --- | --- | --- | --- | --- | --- | --- | --- | --- | --- |
| **Italy** | UBA | C | I 0.5.1; ReDCap | Puglia, South of Italy | 07/20-09/15 | 03/09-05/04: Lockdown order for 100% non-essential, followed by nearly full reopening (mask obligatory) ~ September | 3-18 | ADI-R, VABS-II/3 | y | WISC-IV; WPPSI-III; WAIS; Leiter-R/III; Merrill-Palmer; PEP-III |
|  | UCA | C |  | South Sardinia, Cagliari Oristano provinces | 07/10-09/10 |  | 3-18 | ADI-R, VABS-II/3; KSADS-PL | y | Griffiths-III; WAIS-IV; WISC-III/IV; WPPSI-III; Leiter-R |
|  | UONPI-LO | C |  | Lodi and surroundings, Lombardy | 07/02-09/17 |  | 3-18 | ADI-R, VABS-I | y | Leiter-R; WPPSI-III; WISC-IV |
|  | UFII | C |  | Napoli metropolitan area, Campania | 07/22-09/23 |  | 3-18 | ADI-R, VABS-II/3, KSADS-PL | y | Griffiths; Leiter; WISC-IV |
|  | UTV | C, R |  | Rome, Center-South of Italy | 06/20-09/20 |  | 3-18 | ABAS-II; VABS- II | y | WPPSI-III; WISC-IV; Leiter; PEP-III |
|  | USS | C, R |  | North Sardinia, Sassari and Olbia provinces | 07/28-09/21 |  | 3-17 | ADI-R, VABS-II/3 or ABAS | y | WPPSI-III; WISC-IV; GMDS-R; Leiter-R, Ravens |
|  | SMF | C |  | Tuscany and Center of Italy | 07/25-08/22 |  | 3-18 | ADI-R, VABS-II/3, KSADS-PL | y | WPPSI-III; WISC-IV; GMDS-R; Griffiths-III |

^a^Indicates actual data collection interval; for 4 samples (TC, UCSF, CMI-AC, POND-CMH) only data from children's survey completed within 2 weeks from the data collection interval of 90% of that sample were included in analyses. See Figure 1 for the data collection intervals of the selected datasets by sample. ^b^The latest 0.5.1 version was used as soon as it was available, a subset of children was administered an earlier version. ^c^ Only administered for a subset of the sample. ^d^AsIAm (https://asiam.ie/). ^e^Culturally adapted to the Irish population. Abbreviations: CMI-AC: Child Mind Institute-Autism Center, New York City; CMI-HBN:CMI-Healthy Brain Network, New York City; TC:Thompson Center, Columbia; UCSF:University of California San Francisco, San Francisco; CADB:Center for Autism and Developing Brain, Weill Cornell Medical College/New York Presbyterian Hospital, White Plains; UAth:University of Athens, National & Kapodistrian University of Athens, School of Medicine, First Department of Pediatrics, Unit of Developmental and Behavioral Pediatrics. “Aghia Sophia” Children’s Hospital, Athens, Greece; POND-CMH:Province of Ontario Neurodevelopmental Network, COVID Mental Health collaboration, Ontario; TCD:Trinity College Dublin, Dublin; UCA: University of Cagliari, Child & Adolescent Neuropsychiatry Unit, A.Cao Paediatric Hospital, G.Brotzu, Cagliari, Italy; UBA:University Bari, Child Neuropsychiatry Unit, Policlinic of Bari, Italy; UFII: University of Naples Federico II, Child and Adolescent Neuropsychiatry Unit, Naples, Italy; SMF:IRCCS Stella Maris Foundation, Pisa (Calambrone), Italy; UTV: UniversityTor Vergata, Rome, Italy; USS: University of Sassari, Child Neuropsychiatry Unit, Azienda Ospedaliero-Universitaria, Sassari; UONPI-LO:Unita' Operativa di Neuropsichiatria dell’ Infanzia e dell' adolescenza, Lodi, Italy; C: Clinic Contact, R: Active or Prior Research Contacts; E: English, G: Greek, I: Italian; Y: yes; ADI-R: Autism Diagnostic Interview- Revised (Rutter, Le Couteur, & Lord, 2003); KSADS: (Kaufman et al., 1997); VABS-II: Vineland-II (Sparrow et al., 2008); VABS-3: Vineland-3 (Sparrow, Cicchetti, & Saulnier, 2016), Adaptive Behavior Assessment Scale, Third Edition (ABAS-III; Harrison & Oakland, 2015); DAS-II: Differential Ability Scales, Second Edition (Elliot, 2007); MSEL:Mullen Scales of Early Learning (Mullen, 1995); WASI-2:Wechsler Abbreviated Scale of Intelligence, Second Edition (Wechsler, 2011); BAYLEY III; Ravens:Ravens Standard Progressive Matrices and Colored Progressive Matrices (Raven, Raven, & Court, J. H., 1998b); WISC-III:Wechsler Intelligence Scale for Children, Third Edition (Wechsler, 1991); WISC-IV:Wechsler Intelligence Scale for Children, fourth edition, Italian version, (Wechsler, 2004); WPPSI-III: Wechsler Preschool and Primary Scale of Intelligence-third edition; Leiter: Leiter International Performance Scale Revised-Visualization and Reasoning battery (Roid et al., 1997); Merrill-Palmer:Merrill-Palmer–Revised Scales of Development (Roid, 2004); PEP-III: Psychoeducational Profile-third edition (Schopler et al., 2005); GMDS-R:Griffiths Mental Development Scales (Griffiths, 1996); GMDS-ER:Griffiths Mental Development Scale (Luiz D et al. 2004).

**Table S2. Clinical Characteristics of the aggregate dataset and 15 contributing samples.**

|  | **Aggregate** | **CMI-AC** | **CMI-HBN** | **CADB** | **TC** | **UCSF** | **POND-CMH** | **TCD** | **UAth** | **UBA** | **UCA** | **UONPI-LO** | **UFII** | **SMF** | **UTV** | **USS** |
| --- | --- | --- | --- | --- | --- | --- | --- | --- | --- | --- | --- | --- | --- | --- | --- | --- |
|  | N=1,275 | n=117 | n=87 | n=72 | n=44 | n=30 | n=300 | n =169 | n=44 | n=64 | n=88 | n=50 | n=37 | n=82 | n=53 | n=38 |
| **AFAR-based baseline characteristics** |  |  |  |  |  |  |  |  |  |  |  |  |  |  |  |  |
| *Symptom Domains (raw scores), M (SD)* |  |  |  |  |  |  |  |  |  |  |  |  |  |  |  |  |
| Adaptive Living Skills | 8.4 (2.5) | 7.7 (1.8) | 7.2 (1.9) | 8.7 (2.6) | 8.1 (2) | 7.4 (1.9) | 8.1 (2.5) | 8.4 (2.4) | 7.3 (2.1) | 8.8 (2.3) | 9.5 (2.9) | 8.4 (2.4) | 9.6 (3.1) | 8.5 (2.4) | 9.5 (2.4) | 11.7 (2.4) |
| RRB-LO | 8.1 (3.5) | 7.4 (3.2) | 7.6 (3.4) | 9.1 (3.2) | 9.5 (3) | 7.9 (3.3) | 7.7 (3.5) | 9.9 (3.6) | 7.3 (3.4) | 7.7 (3.3) | 8 (3.4) | 6.9 (2.7) | 7.7 (3.4) | 7.2 (3.1) | 8.3 (3.3) | 8.8 (3.5) |
| RRB-HO | 6.0 (2.2) | 5.7 (2.2) | 5.9 (2.2) | 6.3 (2.1) | 6.6 (1.9) | 6.1 (2.1) | 5.7 (2.2) | 6.9 (2.1) | 6 (2.2) | 5.9 (2.2) | 5.8 (2.1) | 5.5 (2.1) | 5.5 (2) | 5.7 (2) | 5.8 (1.9) | 6.2 (2.2) |
| Activity/Inattention | 4.9 (2.4) | 5.2 (2.4) | 5.1 (2.4) | 5.4 (2.4) | 5.3 (2.5) | 3.9 (1.9) | 5 (2.2) | 4.4 (2.2) | 3.9 (1.8) | 5.1 (2.4) | 6.1 (2.7) | 4.1 (1.8) | 4.1 (2.4) | 4.4 (2.2) | 4.5 (2.3) | 5.7 (2.7) |
| Oppositional | 8.4 (4.8) | 8.8 (4.8) | 8.6 (4.3) | 7.8 (4.6) | 9.7 (5) | 8 (5.2) | 8.4 (4.7) | 7.6 (4.9) | 7.3 (3.1) | 8.8 (4.6) | 11.3 (5.7) | 6.5 (3.6) | 7.1 (4) | 8.1 (4.7) | 6.6 (3.9) | 11 (4.8) |
| Anxiety/Affect | 6.7 (3.5) | 6.2 (3.3) | 5.8 (2.6) | 6.1 (2.9) | 7.8 (3.8) | 5.8 (3.2) | 6.2 (3.1) | 6.6 (3.5) | 6.8 (3.2) | 7.1 (3.3) | 8.6 (4.5) | 6.5 (2.7) | 6.1 (3.5) | 7.2 (3.8) | 6.3 (3.5) | 8.8 (4.2) |
| Sleep Problems | 4.9 (2.1) | 4.7 (1.9) | 4.4 (1.8) | 4.5 (2.1) | 5.1 (2.3) | 4.9 (1.9) | 5.4 (2) | 5.7 (2.2) | 4.1 (1.7) | 4.2 (2) | 5.1 (2.1) | 3.7 (1.7) | 3.9 (2.1) | 4.4 (1.7) | 4.7 (1.9) | 5.6 (2.3) |
|  |  |  |  |  |  |  |  |  |  |  |  |  |  |  |  |  |
|  |  |  |  |  |  |  |  |  |  |  |  |  |  |  |  |  |
|  |  |  |  |  |  |  |  |  |  |  |  |  |  |  |  |  |
|  | **Aggregate** | **CMI-AC** | **CMI-HBN** | **CADB** | **TC** | **UCSF** | **POND-CMH** | **TCD** | **UAth** | **UBA** | **UCA** | **UONPI-LO** | **UFII** | **SMF** | **UTV** | **USS** |
| *Total number of services received prior to COVID-19 (raw scores), M (SD)* |  |  |  |  |  |  |  |  |  |  |  |  |  |  |  |  |
| School services | 2.3 (2.0) | 2.6 (1.8) | 3.2 (1.9) | 4.1 (1.5) | 2.4 (1.7) | 2.8 (1.6) | 1.7 (1.7) | 1.9 (1.6) | 2.6 (2.7) | 2.2 (2.1) | 2.2 (2.4) | 1.9 (1.5) | 2.5 (1.8) | 1.4 (1.7) | 2.5 (1.9) | 3.2 (2.5) |
| Outside School services | 2.1 (2.1) | 1.3 (1.6) | 1.9 (2) | 2.3 (2) | 1.3 (1.3) | 1.3 (1.1) | 1.6 (1.8) | 1.6 (1.8) | 2.9 (2.8) | 3 (2.3) | 3.2 (2.7) | 2 (1.6) | 2.6 (2) | 2.5 (2.1) | 3.5 (2) | 4.4 (2.3) |
| **Prior baseline characteristics** |  |  |  |  |  |  |  |  |  |  |  |  |  |  |  |  |
| *Neuropsychiatric Diagnoses, # (%)* |  |  |  |  |  |  |  |  |  |  |  |  |  |  |  |  |
| ASD | 1,004 (79) | 51 (44) | 87 (100) | 72 (100) | 29 (66) | 21 (70) | 146 (49) | 151 (89) | 44 (100) | 64 (100) | 82 (93) | 50 (100) | 37 (100) | 82 (100) | 53 (100) | 35 (92) |
| ADHD | 484 (38) | 81 (69) | 75 (86) | 4 (6) | 24 (55) | 9 (30) | 129 (43) | 37 (22) | 13 (30) | 20 (31) | 43 (49) | 0 | 3 (8) | 31 (38) | 4 (8) | 11 (29) |
| Intellectual Disability | 132 (10) | 0 | 1 (1) | 3 (4) | 1 (2) | 0 | 11 (4) | 15 (9) | 5 (11) | 7 (11) | 49 (56) | 0 | 9 (24) | 1 (1) | 6 (11) | 24 (63) |
| Borderline Intellectual Functioning | 2 (0) | 0 | 0 | 0 | 1 (2) | 0 | 0 | 0 | 0 | 0 | 0 | 0 | 0 | 0 | 1 (2) | 0 |
| Global Developmental Delay | 4 (0) | 0 | 0 | 0 | 0 | 0 | 0 | 0 | 0 | 0 | 4 (5) | 0 | 0 | 0 | 0 | 0 |
| Anxiety Disorder | 111 (9) | 30 (26) | 37 (43) | 3 (4) | 6 (14) | 6 (20) | 4 (1) | 6 (4) | 0 | 3 (5) | 5 (6) | 0 | 0 | 10 (12) | 1 (2) | 0 |
| Language, Learning, or Other NDD | 94 (7) | 6 (5) | 22 (25) | 3 (4) | 11 (25) | 4 (13) | 3 (1) | 2 (1) | 2 (5) | 13 (20) | 15 (17) | 0 | 0 | 11 (13) | 2 (4) | 0 |
|  |  |  |  |  |  |  |  |  |  |  |  |  |  |  |  |  |
|  | **Aggregate** | **CMI-AC** | **CMI-HBN** | **CADB** | **TC** | **UCSF** | **POND-CMH** | **TCD** | **UAth** | **UBA** | **UCA** | **UONPI-LO** | **UFII** | **SMF** | **UTV** | **USS** |
| Disruptive, Impulse-Control, or Conduct Disorders | 73 (6) | 14 (12) | 12 (14) | 1 (1) | 7 (16) | 0 | 0 | 5 (3) | 1 (2) | 0 | 16 (18) | 0 | 0 | 13 (16) | 1 (2) | 3 (8) |
| Obsessive Compulsive Disorders | 30 (2) | 1 (1) | 5 (6) | 0 | 0 | 0 | 15 (5) | 1 (1) | 1 (2) | 0 | 2 (2) | 0 | 0 | 5 (6) | 0 | 0 |
| Mood Disorders | 23 (2) | 5 (4) | 5 (6) | 0 | 4 (9) | 1 (3) | 0 | 0 | 0 | 1 (2) | 7 (8) | 0 | 0 | 0 | 0 | 0 |
| Motor or Tic Disorders | 19 (1) | 3 (3) | 5 (6) | 0 | 1 (2) | 0 | 0 | 2 (1) | 0 | 3 (5) | 4 (5) | 0 | 0 | 1 (1) | 0 | 0 |
| Trauma, Psychosis, Substance Use & Personality Disorders | 16 (1) | 1 (1) | 6 (7) | 0 | 2 (5) | 0 | 0 | 1 (1) | 0 | 0 | 6 (7) | 0 | 0 | 0 | 0 | 0 |
| Known Genetic/Medical Conditions | 26 (2) | 0 | 0 | 0 | 0 | 0 | 9 (2) | 3 (2) | 7 (16) | 5 (8) | 2 (2) | 0 | 0 | 0 | 0 | 0 |
| *ASD w or w/o Comorbidities, N (%)* |  |  |  |  |  |  |  |  |  |  |  |  |  |  |  |  |
| ASD w/o Comorbidities | 570 (57) | 16 (31) | 4 (5) | 59 (82) | 6 (21) | 15 (71) | 134 (92) | 122 (81) | 20 (45) | 30 (47) | 10 (12) | 50 (100) | 25 (68) | 27 (33) | 40 (75) | 12 (34) |
| ASD + 1 Comorbidity | 232 (23) | 23 (45) | 22 (25) | 12 (17) | 10 (34) | 4 (19) | 12 (8) | 15 (10) | 19 (43) | 16 (25) | 27 (33) | 0 (0) | 12 (32) | 36 (44) | 12 (23) | 12 (34) |
| ASD > 1 Comorbidities | 202 (20) | 12 (24) | 61 (70) | 1 (1) | 13 (45) | 2 (10) | 0 (0) | 14 (9) | 5 (11) | 18 (28) | 45 (55) | 0 (0) | 0 (0) | 19 (23) | 1 (2) | 11 (31) |
| *Full-scale IQ ^b^* |  |  |  |  |  |  |  |  |  |  |  |  |  |  |  |  |
| Mean (SD) | 92.9 (23.5) | 104.8 (17.3) | 98.9 (17.5) | 85.8 (28.6) | 88.5 (13.9) | 108.8 (15.3) | 92 (23.5) | - | 105 (20.4) | 92.9 (26.6) | 83.7 (22.2) | 83.4 (17.9) | 77.2 (20.2) | 94.3 (21.7) | 77.1 (29.6) | 58.7 (33.3) |
| Range | 2-158 | 67-158 | 52-139 | 15-143 | 52-132 | 84-140 | 2-142 | - | 70-121 | 44-139 | 40-124 | 43-123 | 50-115 | 48-137 | 25-128 | 16-110 |
| *N* | 891^b^ | 117 | 87 | 72 | 44 | 29 | 300 | - | 5 | 33 | 41 | 34 | 12 | 82 | 18 | 15 |
|  | **Aggregate** | **CMI-AC** | **CMI-HBN** | **CADB** | **TC** | **UCSF** | **POND-CMH** | **TCD** | **UAth** | **UBA** | **UCA** | **UONPI-LO** | **UFII** | **SMF** | **UTV** | **USS** |
| *Verbal IQ* |  |  |  |  |  |  |  |  |  |  |  |  |  |  |  |  |
| Mean (SD) | 95.6 (24.9) | 107.4 (17.4) | 103.4 (17.4) | 82.8 (33.2) | 91.6 (13.5) | 110.2 (20.1) | 91.7 (26.3) | - | 97.8 (17.5) | 101.5 (24.8) | 76 (20) | - | 82.4 (16.8) | 93.7 (24.5) | - | 82.6 (25.6) |
| Range | 3-170 | 70-170 | 62-139 | 9-136 | 59-121 | 77-155 | 3-160 | - | 70-117 | 54-138 | 26-108 | - | 62-110 | 50-148 | - | 45-124 |
| *N* | 717 | 116 | 87 | 69 | 39 | 25 | 234 | - | 5 | 29 | 11 | - | 9 | 81 | - | 11 |
| *Non-verbal IQ* |  |  |  |  |  |  |  |  |  |  |  |  |  |  |  |  |
| Mean (SD) | 94.2 (24.8) | 104.4 (18.6) | 101.7 (16.7) | 89.9 (27.4) | 92 (14.7) | 106.9 (14.4) | 93.2 (26.2) | - | - | 89.2 (27.9) | 71.9 (24.3) | - | 76 (25.4) | 101.7 (21.9) | 79.6 (27.4) | 80.6 (26.3) |
| Range | 2-162 | 58-162 | 52-140 | 16-158 | 60-139 | 88-140 | 2-147 | - | - | 19-148 | 27-115 | - | 36-119 | 50-148 | 36-153 | 38-120 |
| *N* | 833 | 117 | 87 | 72 | 44 | 25 | 237 | - | - | 57 | 42 | - | 19 | 82 | 35 | 11 |
| *ADOS-2* |  |  |  |  |  |  |  |  |  |  |  |  |  |  |  |  |
| Total CSS | 6.4 (2.4) | 5.2 (2.8) | 7.2 (2.8) | 7.7 (1.9) | 6.2 (2.7) | - | 7 (2.3) | - | - | - | - | - | - | 5.6 (1.3) | 6.8 (1.6) | - |
| N | 479 | 116 | 12 | 70 | 43 | - | 108 | - | - | - | - | - | - | 81 | 48 | - |
| RRB CSS | 6.2 (3.1) | 5.1 (3.3) | 6.4 (2.6) | 8 (1.7) | - | - | - | - | - | - | - | - | - | - | - | - |
| N | 198 | 116 | 12 | 70 | - | - | - | - | - | - | - | - | - | - | - | - |
| Social Affect CSS | 6.3 (2.6) | 5.6 (2.6) | 7.4 (2.7) | 7.4 (2.1) | - | - | - | - | - | - | - | - | - | - | - | - |
| N | 198 | 116 | 12 | 70 | - | - | - | - | - | - | - | - | - | - | - | - |
| Expressive Language score | 6.2 (2.2) | 7.8 (0.5) | 7.8 (0.4) | 6 (2.2) | 7.7 (0.6) | 7.3 (1.2) | 6.1 (2.2) | - | - | 5.5 (2.5) | 5.6 (2.4) | 4.8 (1.9) | 3.9 (2.4) | 6.6 (1.9) | 4.7 (2.5) | 5.4 (2.1) |
| N | 730 | 116 | 12 | 72 | 44 | 23 | 119 | - | - | 64 | 52 | 48 | 37 | 81 | 44 | 18 |

|  | **Aggregate** | **CMI-AC** | **CMI-HBN** | **CADB** | **TC** | **UCSF** | **POND-CMH** | **TCD** | **UAth** | **UBA** | **UCA** | **UONPI-LO** | **UFII** | **SMF** | **UTV** | **USS** |
| --- | --- | --- | --- | --- | --- | --- | --- | --- | --- | --- | --- | --- | --- | --- | --- | --- |
| *Adaptive Functioning, M(SD)* |  |  |  |  |  |  |  |  |  |  |  |  |  |  |  |  |
| Overall Adaptive Composite scores (VABS+ABAS) ^c^ | 71.3 (18.9) | 78.9 (11.8) | - | 73.2 (14.5) | 74.4 (17.1) | 77.9 (10.5) | 75.9 (16.8) | - | - | 48.8 (18.5) | - | - | 66.5 (30.3) | 69.3 (15.8) | 59 (16) | - |
| N | 674 | 100 | - | 64 | 41 | 26 | 238 | - | - | 64 | - | - | 33 | 61 | 47 | - |
| *Child Behavior Checklist T scores, M (SD)* |  |  |  |  |  |  |  |  |  |  |  |  |  |  |  |  |
| Total problems | 64.1 (11.2) | 62.9 (9.1) | 61.8 (8.4) | 60.1 (10.9) | 68.5 (13) | 66.2 (9.4) | 66.2 (11.3) | - | - | 67 (7.1) | - | - | 59.1 (8.9) | 62.7 (10.6) | 60.6 (9.7) | 72.9 (7.3) |
| N | 748 | 105 | 80 | 66 | 40 | 19 | 247 | - | - | 13 | - | - | 37 | 82 | 42 | 14 |
| Externalizing problems | 59 (10.9) | 58.7 (9.9) | 56.6 (10.9) | 56.9 (11.9) | 64.3 (10.3) | 61.4 (9.6) | 60.6 (10.6) | - | - | 61 (6.4) | - | - | 55.4 (10.5) | 57.4 (11.3) | 55.6 (9.4) | 70.4 (7.2) |
| N | 716 | 105 | 80 | 66 | 40 | 19 | 215 | - | - | 13 | - | - | 37 | 82 | 42 | 14 |
| Internalizing problems | 62.3 (11.6) | 60 (11.4) | 59.8 (9.4) | 57.9 (11.2) | 67.5 (12.5) | 65.5 (10.4) | 64 (12.7) | - | - | 65.2 (7.6) | - | - | 58.5 (9.9) | 64.6 (10.4) | 60.4 (8.8) | 69.1 (9.9) |
| N | 749 | 106 | 80 | 66 | 40 | 19 | 247 | - | - | 13 | - | - | 37 | 82 | 42 | 14 |
| *Current Medication use, N (%)* |  |  |  |  |  |  |  |  |  |  |  |  |  |  |  |  |
| Yes | 489 (38) | 44 (38) | 32 (37) | 24 (33) | 27 (61) | 8 (27) | 166 (55) | 65 (38) | 2 (5) | 14 (22) | 50 (57) | 4 (8) | 5 (14) | 34 (41) | 4 (8) | 10 (26) |
| *N* | 1274 | 117 | 86 | 72 | 44 | 30 | 300 | 169 | 44 | 64 | 88 | 50 | 37 | 82 | 53 | 38 |
|  |  |  |  |  |  |  |  |  |  |  |  |  |  |  |  |  |
|  |  |  |  |  |  |  |  |  |  |  |  |  |  |  |  |  |
|  |  |  |  |  |  |  |  |  |  |  |  |  |  |  |  |  |
|  |  |  |  |  |  |  |  |  |  |  |  |  |  |  |  |  |
|  | **Aggregate** | **CMI-AC** | **CMI-HBN** | **CADB** | **TC** | **UCSF** | **POND-CMH** | **TCD** | **UAth** | **UBA** | **UCA** | **UONPI-LO** | **UFII** | **SMF** | **UTV** | **USS** |
| *Time of collection difference (years), M (SD)* |  |  |  |  |  |  |  |  |  |  |  |  |  |  |  |  |
| IQ test administration | 2.2 (3.3) | 1.4 (1.2) | 1.5 (1.1) | 3.3 (1.9) | 1.1 (0.9) | 2.5 (1.6) | 3.3 (2.1) | - | 4.4 (1.2) | 1.4 (2.5) | 2.6 (2.7) | 2.5 (1.7) | 1.8 (1.8) | 1.7 (1.7) | 1.5 (1.4) | 2 (1.6) |
| ADOS-2 administration | 2.1 (2.2) | 1.6 (2.8) | 0.7 (0.5) | 2.5 (1.7) | 1.1 (0.9) | 3.3 (2.1) | 2.8 (2.1) | - | - | 1 (1.9) | 2.3 (3.1) | 2.5 (1.7) | 2.4 (2.2) | 1.8 (1.8) | 1.7 (1.1) | 4 (2) |
| VABS/ABAS administration | 2.2 (1.8) | 1.5 (1.1) | - | 2.5 (1.8) | 1.1 (0.5) | 2.3 (1.5) | 3.1 (2.2) | - | - | 1.5 (1.2) | - | - | 1.8 (1.6) | 2 (1.9) | 1.2 (1) | - |
| CBCL administration | 2 (2) | 1.4 (1.2) | 1.4 (1.2) | 2.4 (1.6) | 1.1 (0.7) | 2.7 (2) | 3.2 (2.3) | - | - | 1.5 (3) | - | - | 0.1 (0.7) | 1.8 (1.8) | 1.7 (1.8) | 1.1 (1.1) |

****** The Mean and Standard Deviation for the above clinical measures collected prior to the pandemic is shown for each contributing sample when the data was available for 5 or more participants. ^a^This includes any diagnoses and double counts those individuals who have more than one diagnoses. ^b^ Out of these n=891 total participants from the aggregate dataset with available FIQ quantitative scores, there are n= 759 with FIQ quantitative scores reported by each contributing samples (when available), n=15 were averaged from VIQ and NVIQ scores , n=63 were DQ scores and n=54 were imputed based on age and diagnosis in those samples with less than 15% data missing (see Supplementary Methods for more details on the imputation method used). ^c^ CMI-HBN, UCA and UONPI-LO administered the VABS to some children of their sample (n=5, n=19, n=50, respectively), however the overall composite scores were not available. Abbreviations: Autism Spectrum Disorder (ASD), Attention-Deficit/Hyperactivity Disorder (ADHD), IC/CD: Impulse-Control and Conduct Disorders; NDD: Neurodevelopmental disorders. Other abbreviations: see Table S1 for site name abbreviated labels.

**Table S3. Participant Characteristics AFAR Background Information for the aggregate dataset (N=1275) and 15 contributing samples.**

|  | **Aggregate** | **CMI-AC** | **CMI-HBN** | **CADB** | **TC** | **UCSF** | **POND-CMH** | **TCD** | **UAth** | **UBA** | **UCA** | **UONPI-LO** | **UFII** | **SMF** | **UTV** | **USS** |
| --- | --- | --- | --- | --- | --- | --- | --- | --- | --- | --- | --- | --- | --- | --- | --- | --- |
|  | N=1,275 | n=117 | n=87 | n=72 | n=44 | n=30 | n=300 | n =169 | n=44 | n=64 | n=88 | n=50 | n=37 | n=82 | n=53 | n=38 |
| **Child Demographics** |  |  |  |  |  |  |  |  |  |  |  |  |  |  |  |  |
| **Age, Years** |  |  |  |  |  |  |  |  |  |  |  |  |  |  |  |  |
| Mean (SD) | 11 (3.6) | 10.2 (2.2) | 12.3 (3.8) | 9.2 (3.3) | 10.1 (3.3) | 10.8 (3.8) | 12.1 (3.5) | 12 (3.3) | 10 (3.1) | 10.4 (3.9) | 11.6 (3.7) | 9.9 (3.1) | 8.7 (3.4) | 10.6 (3.5) | 10.2 (3.7) | 9.2 (3.5) |
| min-max | 5-21 | 6-15 | 6-21 | 5-19 | 6-17 | 5-18 | 5-18 | 5-19 | 5-16 | 5-18 | 5-18 | 5-16 | 5-17 | 5-19 | 5-17 | 5-16 |
| N | 1275 | 117 | 87 | 72 | 44 | 30 | 300 | 169 | 44 | 64 | 88 | 50 | 37 | 82 | 53 | 38 |
| **Sex^a^ #, (%)** |  |  |  |  |  |  |  |  |  |  |  |  |  |  |  |  |
| Males | 996 (78) | 89 (76) | 73 (84) | 60 (83) | 32 (73) | 19 (63) | 220 (73) | 136 (80) | 35 (80) | 52 (81) | 75 (85) | 43 (86) | 27 (73) | 66 (80) | 44 (83) | 25 (66) |
| Females | 277 (22) | 28 (24) | 14 (16) | 12 (17) | 12 (27) | 11 (37) | 79 (26) | 32 (19) | 9 (20) | 12 (19) | 13 (15) | 7 (14) | 10 (27) | 16 (20) | 9 (17) | 13 (34) |
| N | 1275 | 117 | 87 | 72 | 44 | 30 | 300 | 169 | 44 | 64 | 88 | 50 | 37 | 82 | 53 | 38 |
| **Gender Identity** |  |  |  |  |  |  |  |  |  |  |  |  |  |  |  |  |
| Boy/Man | 932 (77) | 89 (76) | 70 (81) | 60 (83) | - | 19 (63) | 219 (73) | 137 (81) | 35 (80) | 47 (80) | 69 (83) | 40 (82) | 25 (71) | 62 (78) | 41 (84) | 19 (59) |
| Girl/Woman | 251 (21) | 26 (22) | 14 (16) | 12 (17) | - | 11 (37) | 78 (26) | 30 (18) | 9 (20) | 10 (17) | 12 (14) | 6 (12) | 10 (29) | 16 (20) | 7 (14) | 10 (31) |
| Transgender | 3 (0) | 1 (1) | 0 | 0 | - | 0 | 1 (0) | 1 (1) | 0 | 0 | 0 | 0 | 0 | 0 | 0 | 0 |
| Non-binary | 4 (0) | 1 (1) | 2 (2) | 0 | - | 0 | 1 (0) | 0 | 0 | 0 | 0 | 0 | 0 | 0 | 0 | 0 |
| Missing | 14 (1) | 0 | 0 | 0 | - | 0 | 1 (0) | 1 (1) | 0 | 2 (3) | 2 (2) | 3 (6) | 0 | 1 (1) | 1 (2) | 3 (9) |
| N | 1204 | 117 | 86 | 72 | - | 30 | 300 | 169 | 44 | 59 | 83 | 49 | 35 | 79 | 49 | 32 |
|  |  |  |  |  |  |  |  |  |  |  |  |  |  |  |  |  |
|  |  |  |  |  |  |  |  |  |  |  |  |  |  |  |  |  |
|  | **Aggregate** | **CMI-AC** | **CMI-HBN** | **CADB** | **TC** | **UCSF** | **POND-CMH** | **TCD** | **UAth** | **UBA** | **UCA** | **UONPI-LO** | **UFII** | **SMF** | **UTV** | **USS** |
| **Hispanic^c^ Ethnicity** | 97 (15) | 35 (30) | 30 (34) | 19 (26) | 2 (5) | 1 (3) | - | - | - | 2 (4) | 2 (3) | 2 (5) | 0 | 1 (2) | 1 (3) | 2 (7) |
| N | 646 | 117 | 87 | 72 | 44 | 30 | - | - | - | 46 | 61 | 41 | 32 | 53 | 34 | 29 |
| **Educational Setting Prior to COVID-19^d^, #, (%)** |  |  |  |  |  |  |  |  |  |  |  |  |  |  |  |  |
| General education school w support | 636 (50) | 64 (55) | 31 (36) | 21 (29) | 25 (57) | 20 (67) | 130 (44) | 73 (43) | 22 (50) | 38 (59) | 46 (52) | 34 (68) | 18 (49) | 58 (71) | 33 (62) | 23 (61) |
| General Education classroom w/o support | 183 (14) | 9 (8) | 4 (5) | 1 (1) | 7 (16) | 2 (7) | 87 (29) | 21 (12) | 13 (30) | 8 (12) | 10 (11) | 3 (6) | 4 (11) | 10 (12) | 3 (6) | 1 (3) |
| Special Education classroom | 379 (30) | 43 (37) | 50 (57) | 48 (67) | 10 (23) | 6 (20) | 68 (23) | 43 (25) | 9 (20) | 13 (20) | 30 (34) | 12 (24) | 13 (35) | 11 (13) | 14 (26) | 9 (24) |
| Specialized program | 72 (6) | 1 (1) | 2 (2) | 2 (3) | 2 (5) | 2 (7) | 10 (3) | 32 (19) | 0 | 5 (8) | 2 (2) | 1 (2) | 2 (5) | 3 (4) | 3 (6) | 5 (13) |
| N | 1270 | 117 | 87 | 72 | 44 | 30 | 295 | 169 | 44 | 64 | 88 | 50 | 37 | 82 | 53 | 38 |
| **Caregiver/Household Demographics** |  |  |  |  |  |  |  |  |  |  |  |  |  |  |  |  |
| **Respondant Age, Years^e^** |  |  |  |  |  |  |  |  |  |  |  |  |  |  |  |  |
| Mean (SD) | 44.3 (6.5) | 43.5 (6.6) | 46.3 (7.3) | 42.5 (6.5) | - | - | - | 45.1 (6.2) | 43.3 (5.4) | 42.5 (6.4) | 45.8 (6.2) | 43.4 (6.1) | 40.6 (6.4) | 45.2 (6.1) | 44.6 (6.7) | 44.1 (5.8) |
| min-max | 24-70 | 26-61 | 32-70 | 30-68 | - | - | - | 31-59 | 30-56 | 26-55 | 32-60 | 31-61 | 24-59 | 30-62 | 29-56 | 31-58 |
| N | 1275 | 117 | 87 | 72 | - | - | - | 169 | 44 | 64 | 88 | 50 | 37 | 82 | 53 | 38 |
|  |  |  |  |  |  |  |  |  |  |  |  |  |  |  |  |  |
|  |  |  |  |  |  |  |  |  |  |  |  |  |  |  |  |  |
|  | **Aggregate** | **CMI-AC** | **CMI-HBN** | **CADB** | **TC** | **UCSF** | **POND-CMH** | **TCD** | **UAth** | **UBA** | **UCA** | **UONPI-LO** | **UFII** | **SMF** | **UTV** | **USS** |
| **Respondent Relationship to Child^f^, #, (%)** |  |  |  |  |  |  |  |  |  |  |  |  |  |  |  |  |
| Mother | 1,062 (86) | 100 (85) | 77 (89) | 65 (90) | - | 26 (87) | 278 (93) | 157 (93) | 37 (84) | 52 (81) | 69 (78) | 42 (84) | 32 (86) | 56 (68) | 41 (77) | 30 (79) |
| Father | 155 (13) | 17 (15) | 10 (11) | 7 (10) | - | 4 (13) | 18 (6) | 11 (7) | 7 (16) | 10 (16) | 17 (19) | 7 (14) | 5 (14) | 24 (29) | 11 (21) | 7 (18) |
| Other | 14 (1) | 0 | 0 | 0 | - | 0 | 4 (1) | 1 (1) | 0 | 2 (3) | 2 (2) | 1 (2) | 0 | 2 (2) | 1 (2) | 1 (3) |
| *N* | 1231 | 117 | 87 | 72 | - | 30 | 300 | 169 | 44 | 64 | 88 | 50 | 37 | 82 | 53 | 38 |
| **Urbanicity, #, (%)** |  |  |  |  |  |  |  |  |  |  |  |  |  |  |  |  |
| Large city | 392 (31) | 86 (74) | 46 (54) | 21 (29) | 3 (7) | 13 (43) | 121 (40) | 19 (11) | 24 (65) | 10 (16) | 12 (14) | 2 (4) | 3 (8) | 7 (9) | 21 (40) | 4 (11) |
| Town, Village, or Rural Area | 356 (28) | 3 (3) | 10 (12) | 21 (29) | 22 (50) | 4 (13) | 36 (12) | 98 (58) | 3 (8) | 24 (38) | 31 (36) | 31 (62) | 21 (57) | 29 (35) | 9 (17) | 14 (37) |
| Suburbs of a large city | 255 (20) | 21 (18) | 28 (33) | 26 (36) | 6 (14) | 9 (30) | 81 (27) | 52 (31) | 6 (16) | 9 (14) | 4 (5) | 1 (2) | 1 (3) | 4 (5) | 5 (9) | 2 (5) |
| Small city | 261 (21) | 7 (6) | 1 (1) | 4 (6) | 13 (30) | 4 (13) | 61 (20) | 0 | 4 (11) | 21 (33) | 40 (46) | 16 (32) | 12 (32) | 42 (51) | 18 (34) | 18 (47) |
| *N* | 1264 | 117 | 85 | 72 | 44 | 30 | 299 | 169 | 37 | 64 | 87 | 50 | 37 | 82 | 53 | 38 |
| **Essential Worker in the household, #, (%)** |  |  |  |  |  |  |  |  |  |  |  |  |  |  |  |  |
| Yes | 397 (31) | 30 (26) | 25 (29) | 20 (28) | 27 (61) | 17 (57) | 96 (32) | 72 (43) | 20 (45) | 7 (11) | 20 (23) | 10 (21) | 8 (22) | 20 (25) | 17 (32) | 8 (21) |
| *N* | 1271 | 117 | 87 | 72 | 44 | 30 | 300 | 169 | 44 | 63 | 88 | 48 | 37 | 81 | 53 | 38 |
|  |  |  |  |  |  |  |  |  |  |  |  |  |  |  |  |  |
|  | **Aggregate** | **CMI-AC** | **CMI-HBN** | **CADB** | **TC** | **UCSF** | **POND-CMH** | **TCD** | **UAth** | **UBA** | **UCA** | **UONPI-LO** | **UFII** | **SMF** | **UTV** | **USS** |
| **Household Composition, #, (%)** |  |  |  |  |  |  |  |  |  |  |  |  |  |  |  |  |
| Two Parents | 884 (69) | 80 (68) | 57 (66) | 51 (71) | 26 (59) | 25 (83) | 196 (65) | 111 (66) | 30 (68) | 52 (81) | 67 (76) | 44 (88) | 25 (68) | 56 (68) | 42 (79) | 22 (58) |
| One Parent | 187 (15) | 19 (16) | 7 (8) | 5 (7) | 6 (14) | 3 (10) | 57 (19) | 30 (18) | 11 (25) | 5 (8) | 10 (11) | 4 (8) | 4 (11) | 12 (15) | 7 (13) | 7 (18) |
| Multigeneration/Other Family members | 125 (10) | 11 (9) | 17 (20) | 7 (10) | 6 (14) | 2 (7) | 36 (12) | 12 (7) | 1 (2) | 4 (6) | 7 (8) | 1 (2) | 1 (3) | 12 (15) | 3 (6) | 5 (13) |
| Other | 79 (6) | 7 (6) | 6 (7) | 9 (12) | 6 (14) | 0 | 11 (4) | 16 (9) | 2 (5) | 3 (5) | 4 (5) | 1 (2) | 7 (19) | 2 (2) | 1 (2) | 4 (11) |
| *N* | 1275 | 117 | 87 | 72 | 44 | 30 | 300 | 169 | 44 | 64 | 88 | 50 | 37 | 82 | 53 | 38 |
| **Siblings, #, (%)** |  |  |  |  |  |  |  |  |  |  |  |  |  |  |  |  |
| Yes | 843 (79) | 74 (75) | 48 (75) | 41 (73) | 26 (81) | 25 (89) | 210 (83) | 116 (82) | 33 (80) | 47 (82) | 55 (71) | 36 (75) | 21 (72) | 49 (72) | 40 (82) | 22 (76) |
| *N* | 1071 | 99 | 64 | 56 | 32 | 28 | 253 | 141 | 41 | 57 | 77 | 48 | 29 | 68 | 49 | 29 |
| **Government assistance, #, (%)** |  |  |  |  |  |  |  |  |  |  |  |  |  |  |  |  |
| Yes | 312 (25) | 4 (3) | 4 (5) | 1 (1) | 5 (11) | 0 | 36 (12) | 23 (14) | 21 (49) | 28 (44) | 51 (58) | 28 (58) | 16 (43) | 29 (36) | 36 (68) | 30 (81) |
| *N* | 1268 | 116 | 87 | 72 | 44 | 30 | 300 | 169 | 43 | 63 | 88 | 48 | 37 | 81 | 53 | 37 |
|  |  |  |  |  |  |  |  |  |  |  |  |  |  |  |  |  |
|  |  |  |  |  |  |  |  |  |  |  |  |  |  |  |  |  |
|  |  |  |  |  |  |  |  |  |  |  |  |  |  |  |  |  |
|  |  |  |  |  |  |  |  |  |  |  |  |  |  |  |  |  |
|  |  |  |  |  |  |  |  |  |  |  |  |  |  |  |  |  |
|  |  |  |  |  |  |  |  |  |  |  |  |  |  |  |  |  |
|  | **Aggregate** | **CMI-AC** | **CMI-HBN** | **CADB** | **TC** | **UCSF** | **POND-CMH** | **TCD** | **UAth** | **UBA** | **UCA** | **UONPI-LO** | **UFII** | **SMF** | **UTV** | **USS** |
| **COVID-19 Health/Job Impact** |  |  |  |  |  |  |  |  |  |  |  |  |  |  |  |  |
| Child Two weeks COVID-19 Exposure, #, (%) |  |  |  |  |  |  |  |  |  |  |  |  |  |  |  |  |
| None | 1,223 (96) | 98 (84) | 73 (84) | 67 (93) | 39 (89) | 30 (100) | 297 (99) | 169 (100) | 44 (100) | 64 (100) | 87 (99) | 46 (92) | 37 (100) | 81 (99) | 53 (100) | 38 (100) |
| Exposure to person with diagnosis | 33 (3) | 9 (8) | 8 (9) | 5 (7) | 5 (11) | 0 | 3 (1) | 0 | 0 | 0 | 0 | 2 (4) | 0 | 1 (1) | 0 | 0 |
| Exposure to person with symptoms | 19 (1) | 10 (9) | 6 (7) | 0 | 0 | 0 | 0 | 0 | 0 | 0 | 1 (1) | 2 (4) | 0 | 0 | 0 | 0 |
| *N* | 1275 | 117 | 87 | 72 | 44 | 30 | 300 | 169 | 44 | 64 | 88 | 50 | 37 | 82 | 53 | 38 |
| **Family members COVID19 diagnosed, #, (%)** |  |  |  |  |  |  |  |  |  |  |  |  |  |  |  |  |
| No diagnosis | 1,213 (95) | 95 (81) | 74 (85) | 63 (88) | 43 (98) | 30 (100) | 297 (99) | 168 (99) | 44 (100) | 62 (97) | 87 (99) | 44 (88) | 37 (100) | 80 (98) | 52 (98) | 37 (97) |
| Yes, non-household member | 40 (3) | 15 (13) | 7 (8) | 4 (6) | 0 | 0 | 1 (0) | 1 (1) | 0 | 2 (3) | 1 (1) | 5 (10) | 0 | 2 (2) | 1 (2) | 1 (3) |
| Yes, household member | 22 (2) | 7 (6) | 6 (7) | 5 (7) | 1 (2) | 0 | 2 (1) | 0 | 0 | 0 | 0 | 1 (2) | 0 | 0 | 0 | 0 |
| *N* | 1275 | 117 | 87 | 72 | 44 | 30 | 300 | 169 | 44 | 64 | 88 | 50 | 37 | 82 | 53 | 38 |
|  |  |  |  |  |  |  |  |  |  |  |  |  |  |  |  |  |
|  |  |  |  |  |  |  |  |  |  |  |  |  |  |  |  |  |
|  |  |  |  |  |  |  |  |  |  |  |  |  |  |  |  |  |
|  |  |  |  |  |  |  |  |  |  |  |  |  |  |  |  |  |
|  | **Aggregate** | **CMI-AC** | **CMI-HBN** | **CADB** | **TC** | **UCSF** | **POND-CMH** | **TCD** | **UAth** | **UBA** | **UCA** | **UONPI-LO** | **UFII** | **SMF** | **UTV** | **USS** |
| **Family members Health/Job Impact^g^, #, (%)** |  |  |  |  |  |  |  |  |  |  |  |  |  |  |  |  |
| Lost job/Reduced income | 237 (19) | 23 (20) | 14 (16) | 12 (17) | 5 (11) | 6 (20) | 85 (28) | 17 (10) | 5 (11) | 10 (16) | 20 (23) | 7 (14) | 4 (11) | 9 (11) | 11 (21) | 9 (24) |
| Self-quarantine | 95 (7) | 10 (9) | 14 (16) | 13 (18) | 8 (18) | 0 | 33 (11) | 2 (1) | 1 (2) | 2 (3) | 4 (5) | 3 (6) | 0 | 2 (2) | 2 (4) | 1 (3) |
| Physical illness/Hospitalized | 45 (4) | 14 (12) | 10 (11) | 3 (4) | 0 | 0 | 10 (3) | 1 (1) | 0 | 1 (2) | 1 (1) | 3 (6) | 1 (3) | 1 (1) | 0 | 0 |
| Passed away | 6 (0) | 2 (2) | 1 (1) | 0 | 0 | 0 | 2 (1) | 0 | 0 | 0 | 0 | 1 (2) | 0 | 0 | 0 | 0 |
| None of the above | 892 (70) | 68 (58) | 48 (55) | 44 (61) | 31 (70) | 24 (80) | 170 (57) | 149 (88) | 38 (86) | 51 (80) | 63 (72) | 36 (72) | 32 (86) | 70 (85) | 40 (75) | 28 (74) |
| N | 1275 | 117 | 87 | 72 | 44 | 30 | 300 | 169 | 44 | 64 | 88 | 50 | 37 | 82 | 53 | 38 |

^a^Question on sex included three response options: male, female and other. n=1 respondent in TCD indicated "Other", n=1 from POND-CMH chose not to answer. ^b^ Gender identity information was not collected at TC (Thompson Center). Among the n=3 respondents selecting transgender: n=1 indicated Trans boy/man and n=2 Trans girl/woman. ^c^The question on Hispanic ethnicity was not included in the POND-CMH, TCD and UAth sample's surveys. ^d^ Special Education classroom refers to public or private schools; Specialized program refers to center- or home-based applied behavioral analysis programs or residential settings and alike. ^e^ Respondent age question was not included in the POND-CMH, TC and UCSF samples' survey. ^f^Respondent relationship to the child was not included in the TC site. ^g^ Self-quarantine summarizes answers indicating self-quarantine with and without symptoms. Other abbreviations: see Table S1 for site name abbreviated labels.

**Table S4. Ancestry of the aggregate dataset (N=1275) and 15 contributing samples.**

|  | **Aggregate** | **CMI-AC** | **CMI-HBN** | **CADB** | **TC ^a^** | **UCSF ^a^** | **POND-CMH ^a^** | **TCD ^a^** | **UAth** | **UBA** | **UCA** | **UONPI-LO** | **UFII** | **SMF** | **UTV** | **USS** |
| --- | --- | --- | --- | --- | --- | --- | --- | --- | --- | --- | --- | --- | --- | --- | --- | --- |
| N (%) | N=1,275 | n=117 | n=87 | n=72 | n=44 | n=30 | n=300 | n =169 | n=44 | n=64 | n=88 | n=50 | n=37 | n=82 | n=53 | n=38 |
| **European/British^b^** | 716 (56) | 27 (23) | 24 (28) | 10 (14) | 0 | 0 | 151 (50) | 156 (92) | 41 (93) | 55 (86) | 56 (64) | 31 (62) | 31 (84) | 64 (78) | 47 (89) | 23 (61) |
| **Asian** | 45 (4) | 6 (5) | 2 (2) | 6 (8) | 0 | 4 (13) | 20 (7) | 2 (2) | 0 | 0 | 0 | 3 (6) | 0 | 0 | 1 (2) | 0 |
| **African** | 16 (1) | 4 (3) | 4 (5) | 4 (6) | 0 | 0 | 1 (0) | 2 (1) | 0 | 0 | 0 | 1 (2) | 0 | 0 | 0 | 0 |
| **Central / South American / Caribbean** | 14 (1) | 6 (5) | 3 (3) | 2 (3) | 0 | 0 | 3 (1) | 0 | 0 | 0 | 0 | 0 | 0 | 0 | 0 | 0 |
| **Middle Eastern** | 8 (1) | 4 (3) | 2 (2) | 1 (1) | 0 | 0 | 0 | 0 | 1 (2) | 0 | 0 | 0 | 0 | 0 | 0 | 0 |
| **Indigenous** | 11 (1) | 1 (1) | 0 | 1 (1) | 1 (2) | 0 | 5 (2) | 0 | 1 (2) | 0 | 1 (1) | 1 (2) | 0 | 0 | 0 | 0 |
| **Other^c^** | 99 (8) | 4 (3) | 3 (3) | 1 (1) | 0 | 0 | 71 (24) | 0 | 1 (2) | 2 (3) | 7 (8) | 1 (2) | 2 (5) | 2 (2) | 1 (2) | 4 (11) |
| **Mixed** | 204 (16) | 61 (52) | 46 (53) | 40 (56) | 0 | 0 | 38 (13) | 0 | 0 | 1 (2) | 3 (3) | 3 (6) | 0 | 7 (9) | 3 (6) | 2 (5) |
| **Missing/Unknown** | 162 (13) | 4 (3) | 3 (3) | 7 (10) | 43 (98) | 26 (87) | 11 (4) | 9 (5) | 0 | 6 (9) | 21 (24) | 10 (20) | 4 (11) | 9 (11) | 1 (2) | 9 (24) |
| ^a^For the TC, UCSF, TCD and POND-CMH samples the response/options referring to ancestry were reworded slightly differently from AFAR. Whenever possible they were remapped into the AFAR structure. Specifically, for the TC and UCSF samples "American Indian," "Asian" and "African American" were mapped into the AFAR categories "Indigenous", "Asian" and "African"; the response "White" could not be obviously remapped into AFAR ancestry categories and was counted under "Missing/Unknown." For TCD "Irish" / "Irish Traveller", "African" and "Other Asian" were mapped into the "European/British," "African" and "Asian" ancestry categories of AFAR, respectively; the responses "Any other white background", "Any other black background" and "Chinese" could not be obviously mapped into AFAR ancestry categories and thus were listed under "Missing/Unknown." For the POND-CMH sample the responses "European Origins," "Caribbean origins" and "Latin, Central, South American origins," "African origins", "Asian origins," "North American Aboriginal origins" and "Oceania origins", "Other north American origins" and "Other" were mapped into "European/British," "Central/South American/Caribbean", "African," "Asian," "Indigenous," and "Other," respectively. ^b^ Includes European, British/Irish, Australian, New Zealand, North American (non-native). ^c^The respondent selected "Other" and was not further specified. Abbreviations: see Table S1 for site name abbreviated labels. | | | | | | | | | | | | | | | | |
|  |  |  |  |  |  |  |  |  |  |  |  |  |  |  |  |  |
|  |  |  |  |  |  |  |  |  |  |  |  |  |  |  |  |  |
|  |  |  |  |  |  |  |  |  |  |  |  |  |  |  |  |  |
|  |  |  |  |  |  |  |  |  |  |  |  |  |  |  |  |  |
|  |  |  |  |  |  |  |  |  |  |  |  |  |  |  |  |  |

**Table S5. EFA Factor Loadings by domain examined (Split-half sample 1, n=636)**

| **Domain/Item** | **Factor/Factor Loadings** | | | | |
| --- | --- | --- | --- | --- | --- |
|  | **F1** | **F2** | **F3** | **F4** | **F5** |
| **Adaptive Living skills** |  |  |  |  |  |
| Play/entertain self | **0.64** |  |  |  |  |
| Structure own activities | **0.77** |  |  |  |  |
| Self-care | **0.80** |  |  |  |  |
| Meal/food behaviors | **0.70** |  |  |  |  |
| **Restricted and Repetitive Behaviors/Interests** |  |  |  |  |  |
| Sensory seeking | **0.72** | -0.01 |  |  |  |
| Repetitive motor mannerisms | **0.80** | -0.04 |  |  |  |
| Rituals/routines | **0.50** | 0.36 |  |  |  |
| Makes family keep routines/rituals | -0.01 | **0.76** |  |  |  |
| Restricted interests | 0.20 | **0.29** |  |  |  |
| *Adjusts to change* | *0.11* | *0.02* |  |  |  |
| **COVID-19 Worries** |  |  |  |  |  |
| Worried about own infection | **0.92** | -0.07 |  |  |  |
| Worried about others’ infection | **0.77** | 0.07 |  |  |  |
| Worried about own physical health | **0.51** | 0.29 |  |  |  |
| Reading/talking about/watching news about COVID-19 | **0.44** | 0.10 |  |  |  |
| *Worried about own mental health* | *0.01* | ***0.99*** |  |  |  |
| **Life Changes** |  |  |  |  |  |
| Financial difficulty | **0.37** | 0.17 | 0.06 |  |  |
| Living difficulty | **0.82** | 0.03 | -0.09 |  |  |
| Food security worry | **0.90** | -0.03 | 0.06 |  |  |
| Restriction stress | 0.05 | **0.60** | 0.25 |  |  |
| Cancellation difficulty | 0.01 | **0.90** | -0.06 |  |  |
| *Hopefully End* | *0.05* | *-0.19* | *0.09* |  |  |
| *Positive change* | *0.00* | *0.01* | ***0.70*** |  |  |
| *Time outside* | *-0.06* | *0.02* | *-0.05* |  |  |
| **Co-Occuring Problem Behaviors** |  |  |  |  |  |
| Angry/Losing temper | **0.83** | 0.01 | 0.08 |  |  |
| Verbal aggression | **0.92** | -0.02 | -0.11 |  |  |
| Physical aggression | **0.62** | 0.02 | 0.16 |  |  |
| Disobedient/arguing | **0.63** | 0.09 | 0.10 |  |  |
| Crying easily | 0.18 | **0.37** | 0.14 |  |  |
| Social worries | 0.04 | **0.61** | -0.05 |  |  |
| Problems separating | -0.07 | **0.63** | 0.07 |  |  |
| Excessive fear | 0.02 | **0.77** | 0.03 |  |  |
| Hyperactivity | 0.02 | -0.02 | **0.75** |  |  |
| Off task behaviors | 0.05 | 0.04 | **0.59** |  |  |
| *Deliberately injuring self* | *0.00* | *0.19* | ***0.27*** |  |  |
| **Daily Behaviors and Media** |  |  |  |  |  |
| Weekday bedtime | **0.78** | -0.03 | -0.05 |  |  |
| Weekend bedtime | **0.91** | 0.00 | 0.04 | -0.03 | -0.02 |
| Weekday hours of sleep | -0.15 | **0.76** | -0.06 | -0.01 | -0.07 |
| Weekend hours of sleep | 0.06 | **0.95** | 0.03 | 0.00 | 0.02 |
| Time watching television | 0.10 | 0.03 | **0.27** | 0.08 | 0.11 |
| Time on social media | 0.07 | 0.04 | **0.55** | 0.02 | 0.11 |
| Time on video games | 0.10 | 0.09 | **0.33** | 0.11 | 0.08 |
| Online interactions with peers^a^ | 0.00 | -0.01 | **0.77** | 0.00 | -0.02 |
| Online interactions with adults^a^ | -0.11 | -0.04 | **0.54** | -0.01 | -0.10 |
| Exercise | 0.09 | -0.04 | 0.03 | **0.53** | 0.03 |
| Time outdoors | -0.01 | 0.01 | 0.02 | **0.84** | 0.00 |
| Difficulties falling asleep | 0.05 | 0.04 | -0.01 | -0.07 | **0.74** |
| Night waking | -0.11 | -0.12 | 0.00 | 0.09 | **0.64** |

^a^Item missed for three participants (n=2 UAth, n=1 POND-CMH).

Legend: The Grey and italicized text indicates items excluded in subsequent confirmatory factor analysis (CFA); items were excluded if their factor loadings resulting from EFA were below 0.03 (n=3 items) or if they resulted as a single item factor (n=2) or based on theoretical plausibility (n=1). See Table S6 for CFA goodness-of-fit indices. Abbreviations: EFA, Exploratory Factor Analysis; F=Factor.

**Table S6. Goodness of Fit Indices for EFA/CFAs.**

| Domain | Analysis Type: Result | χ^2^ | RMSEA | TLI | CFI |
| --- | --- | --- | --- | --- | --- |
| Adaptive Living Skills | EFA: 1 factor | <0.01 | 0.03 | 0.97 |  |
|  | CFA-P: 1 factor | 0.20 | 0.03 | 1.00 | 1.00 |
|  | CFA-S: 1 factor | 0.13 | 0.03 | 1.00 | 1.00 |
| RRB | EFA: 2 factors, 6 items | <0.66 | 0.05 | 1.01 |  |
|  | CFA-P: 2 factors, 5 items | 0.03 | 0.05 | 1.00 | 1.00 |
|  | CFA-S: 2 factors, 5 items | <0.01 | 0.07 | 0.99 | 1.00 |
| Co-Occurring Problem Behaviors | EFA: 3 factors, 11 items | <0.00 | 0.06 | 0.95 |  |
|  | CFA-P: 3 factors, 10 items | <0.01 | 0.06 | 0.99 | 1.00 |
|  | CFA-S: 3 factors, 10 items | <0.01 | 0.07 | 0.99 | 0.99 |
| Daily Behaviors | EFA: 5 factors, 13 items | <0.00 | 0.09 | 0.83 |  |
|  | CFA-P: 5 factors, 13 items | <0.00 | 0.07 | 0.99 | 0.99 |
|  | CFA-S: 5 factors, 13 items | <0.01 | 0.11 | 0.99 | 1.00 |
| COVID-19 Worries^a^ | EFA: 2 factors | <0.35 | 0.00 | 1.00 |  |
|  | CFA-P: 1 factor, 4 items | 0.58 | 0.00 | 1.00 | 1.00 |
| Life Changes^a^ | EFA: 3 factors | <0.97 | 0.03 | 0.98 |  |
|  | CFA-P: 2 factors, 5 items | 0.47 | 0.00 | 1.00 | 1.00 |

^a^These domains did not have Prior and Current time points; therefore, their EFA was made up of split-half 1 current scores (n=636) and CFA-P conducted on split-half 2 (n=637) current scores. Abbreviations: EFA, Primary EFA conducted on split-half sample 1 (n=636) using prior three month scores; CFA-P, Primary CFA conducted on split-half sample 2 (n=637) using the prior three months to COVID-19 pandemic scores; CFA-S, Secondary CFA was conducted on the whole sample using the current scores (n= 1275); χ2, Chi-square (non-significant values suggest good fit); RMSEA, root-mean-square error of approximation (cutoffs of .01, .05, and .08 indicate excellent, good, and acceptable fit, respectively3); TLI=Tucker-Lewis index4 (≥.95 indicates good fitting models5); CFI, Bentler’s comparative fit index6 (≥.96 indicates goodness of fit)

**Table S7. Group means of symptom changes and number of services lost and modified in the aggregate dataset and by COVID19 impact subgroups.**

| Characteristic | **Aggregate** | **S1** | **S2** | **S3** | **S4** |  | **ANOVA Subgroup comparisons** | | |
| --- | --- | --- | --- | --- | --- | --- | --- | --- | --- |
|  | N=1275 | (n=251,20) | (n=653,51) | (n=293, 23%) | (n=78, 6%) | *F (df1-df2)* | *P-value adj.* | $\eta_{p}^{2}$ | Post Hoc comparisons |
| **Symptom Domain Change (Current-Prior), M (SD)^a^** |  |  |  |  |  |  |  |  |  |
| Adaptive Living Skills | 0.3 (1.6) | 1.7 (2) | -0.1 (1.3) | 0 (1.3) | -0.3 (1.2) | 102.9 (3-1271) | P<0.001 | 0.20 | S1 > S3 = S2 = S4 |
| RRB-LO | ﻿-0.1 (2.1) | 1.4 (2.5) | -0.5 (1.7) | -0.5 (1.8) | -0.5 (2.1) | 67.4 (3-1271) | P<0.001 | 0.14 | S1 > S3 = S2 = S4 |
| RRB-HO | ﻿-0.1 (1.6) | 0.8 (1.7) | -0.2 (1.6) | -0.2 (1.2) | -0.7 (1.5) | 33.5 (3-1271) | P<0.001 | 0.07 | S1 > S3 = S2 = S4 |
| Activity/Inattention | 0.1 (1.8) | 1.4 (2) | -0.3 (1.6) | -0.1 (1.4) | -0.3 (1.6) | 75.1 (3-1271) | P<0.001 | 0.15 | S1 > S3 = S2 = S4 |
| Oppositional | 0.3 (3) | 2.9 (3.7) | -0.5 (2.6) | 0.2 (1.7) | -0.8 (2.5) | 104.6 (3-1271) | P<0.001 | 0.20 | S1 > S3 > S2 = S4 |
| Anxiety | 0.3 (2.8) | 2.9 (3.6) | -0.6 (2.1) | 0.2 (1.8) | -0.8 (2.5) | 130.5 (3-1271) | P<0.001 | 0.24 | S1 > S3 > S2 = S4 |
| Sleep Problems | 0.2 (1.5) | 1.7 (1.8) | -0.2 (1.2) | -0.1 (1.1) | 0.2 (1.4) | 122.4 (3-1271) | P<0.001 | 0.22 | S1 > S3 = S2 = S4 |
| **Total Raw Number of Services, M (SD)** |  |  |  |  |  |  |  |  |  |
| Lost School Services | 1.2 (1.7) | 1.3 (1.7) | 1.0 (1.3) | 0.5 (0.8) | 5.3 (1.5) | 279.5 (3-1271) | P<0.001 | 0.40 | S4 > S1 = S2 > S3 |
| Lost Out. School Services | 1.3 (2) | 1.4 (1.9) | 1.0 (1.5) | 0.5 (1) | 6.1 (1.7) | 311.1 (3-1271) | P<0.001 | 0.42 | S4 > S1 = S2 > S3 |
| Continued School Services | 1.1 (1.4) | 0.7 (1.1) | 0.5 (0.8) | 3 (1.7) | 0.3 (0.6) | 306.9 (3-1271) | P<0.001 | 0.42 | S3 > S1 = S2 = S4 |
| Continued Out. School Services | 0.8 (1.3) | 0.6 (0.8) | 0.4 (0.7) | 2.1 (1.7) | 0.3 (0.5) | 193.8 (3-1271) | P<0.001 | 0.31 | S3 > S1 = S2 = S4 |
| ^a^Aggregate and subgroup scores are raw difference scores from each domain, shown as M (SD).Abbreviations: S1, Broad symptom worsening only subgroup; S2, Average symptom/service changes subgroup; S3, Primarily modified services subgroup; S4, Primarily lost services subgroup; RRB LO, Restricted and Repetitive Behaviors - Lower Order; RRB-HO, Restricted and Repetitive Behaviors - Higher Order; Out., Outside; df1-df2,numerator and denominator degrees of freedom, P-value adj., P-values adjusted for FDR-correction (⍺=.05); $\eta_{p}^{2}$ , eta squared effect size.  **Table S8. Group means of demographic and clinical characteristics in the aggregate dataset and by subgroups.**   \| Characteristics \| **Aggregate** \| **S1** \| **S2** \| **S3** \| **S4** \| **ANOVA Subtype comparisons** \| \| \| \| \| --- \| --- \| --- \| --- \| --- \| --- \| --- \| --- \| --- \| --- \| \|  \| N=1244 \| (n=249, 20%) \| (n=637, 51%) \| (n=283, 23%) \| (n=75, 6%) \| *F, X_2_*  *(df1-df2)* \| *P-value adj.* \| $\eta_{p}^{2}$ */ Cohen W* \| Post Hoc  comparisons \| \| \| **Child age**, years Mean (SD) \| 11 (3.6)  [5-21] \| 10.8 (3.6) [5-18] \| 11.6 (3.5) [5-21] \| 10.2 (3.4) [5-21] \| 9.7 (3.1) [5-16] \| 15.84  (3,1240) \| P<0.001 \| 0.037 \| S2 > (S1 > S4 = S3) \| \| \| **Child sex**, N (%), males \|  \| 190 (76%) \| 502 (79%) \| 221 (78%) \| 59 (79%) \| 0.68  (3, 1240) \| 0.881 \| 0.023 \| - \| \| \| Intellectual abilities categories, N (%)^a^ \|  \|  \|  \|  \|  \| 46.66  (3, 18) \| P<0.001 \| 0.225 \|  \| \| \| *Above average* \| 136 (11) \| 27 (11) \| 74 (12) \| 32 (11) \| 3 (4) \|  \|  \|  \| S4 < (S1 = S3 = S2) \| \| \| *Average* \| 481 (39) \| 87 (35) \| 258 (41) \| 120 (42) \| 16 (21) \|  \|  \|  \| S4 < (S1 = S3 = S2) \| \| \| *Borderline* \| 138 (11) \| 28 (11) \| 66 (10) \| 36 (13) \| 8 (11) \|  \|  \|  \| - \| \| \| *Mild* \| 87 (7) \| 14 (6) \| 39 (6) \| 27 (10) \| 7 (9) \|  \|  \|  \| - \| \| \| *Moderate* \| 53 (4) \| 10 (4) \| 24 (4) \| 10 (4) \| 9 (12) \|  \|  \|  \| S4 > (S1 = S3 = S2) \| \| \| *Severe* \| 18 (1) \| 2 (1) \| 8 (1) \| 3 (1) \| 5 (7) \|  \|  \|  \| S4 > (S1 = S3 = S2) \| \| \| *Profound* \| 7 (1) \| 0 (0) \| 4 (1) \| 2 (1) \| 1 (1) \|  \|  \|  \| - \| \| \| Full-scale IQ, M (SD)^b^ \| 93 (23) \| 93 (24) \| 95 (22) \| 93 (23) \| 76 (27) \| 9.08  (3, 871) \| P<0.001 \| 0.030 \| S4 < (S1 = S3 = S2) \| \|   a n=324 (26%) children were missing qualitative intelligence estimates. For more details on how these intellectual functioning categories were derived see Supplementary methods. b Out of n=1244 total children, n=369 (30%) were missing standardized full-scale IQ scores. Abbreviations: S1, Broad symptom worsening subgroup; S2, Average symptom/service changes subgroup; S3, Mostly modified services subgroup; S4, Mostly lost services subgroup; df1-df2,numerator and denominator degrees of freedom, P-value adj., P-values adjusted for FDR-correction (⍺=.05); 𝜂_𝑝^2, eta squared effect size; Cohen W, Cohen’s effect size for categorical variables. | | | | | | | | | |
|  |  |  |  |  |  |  |  |  |  |
|  |  |  |  |  |  |  |  |  |  |
|  |  |  |  |  |  |  |  |  |  |

**4. Supplementary Figures**


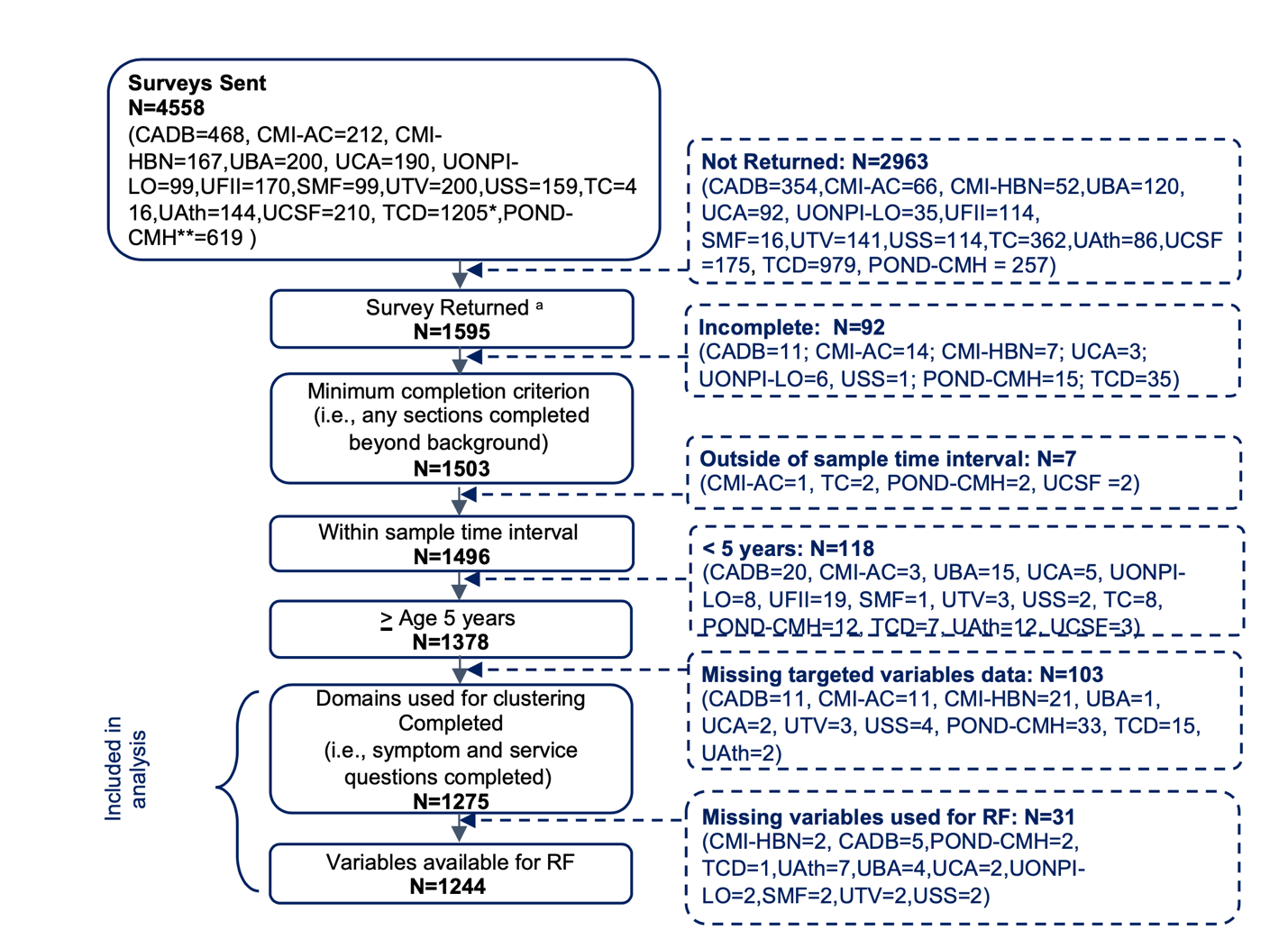


**Fig S1. Data collection and selection flow.**

Across 14 institutions (15 samples) 4558 families are known to be contacted to complete the survey. Of them, a total of N=1595 (36%) CRISIS AFAR baseline parent-based surveys were returned with return rate varying by sample (CADB=114, 24%; CMI-AC=146, 69%; CMI-HBN=115, 69%: UBA=80, 40%; UCA=98, 52%; UONPI-LO=64, 65%;UFII=56 ,33%; SMF=83, 84%,UTV=59 ,30%;USS=45,28%;TC=53,13%; UAth=58,40%; UCSF=35,17%; TCD*=226,19%; POND-CMH**=362,58% . Of those returned, N=1503 of which were completed for at least two sections including background. For data analyses we selected those completed survey within 2 weeks from the completion time interval of 90% of a given sample (N=1496), of children aged 5 years-old and above (N=1378) who responded to AFAR behavioral and service domains examined with hierarchical clustering. This yielded a final aggregate dataset of N=1275 across the 15 contributing samples. See Figure 1 for site name abbreviated labels. *For TCD sample, the total number of surveys sent for one of the sources is unknown. **For POND-CMH sample the original contact count includes a larger group of children than those with NDD targeted here. Abbreviations: see Table S1 for site name abbreviated labels.

**
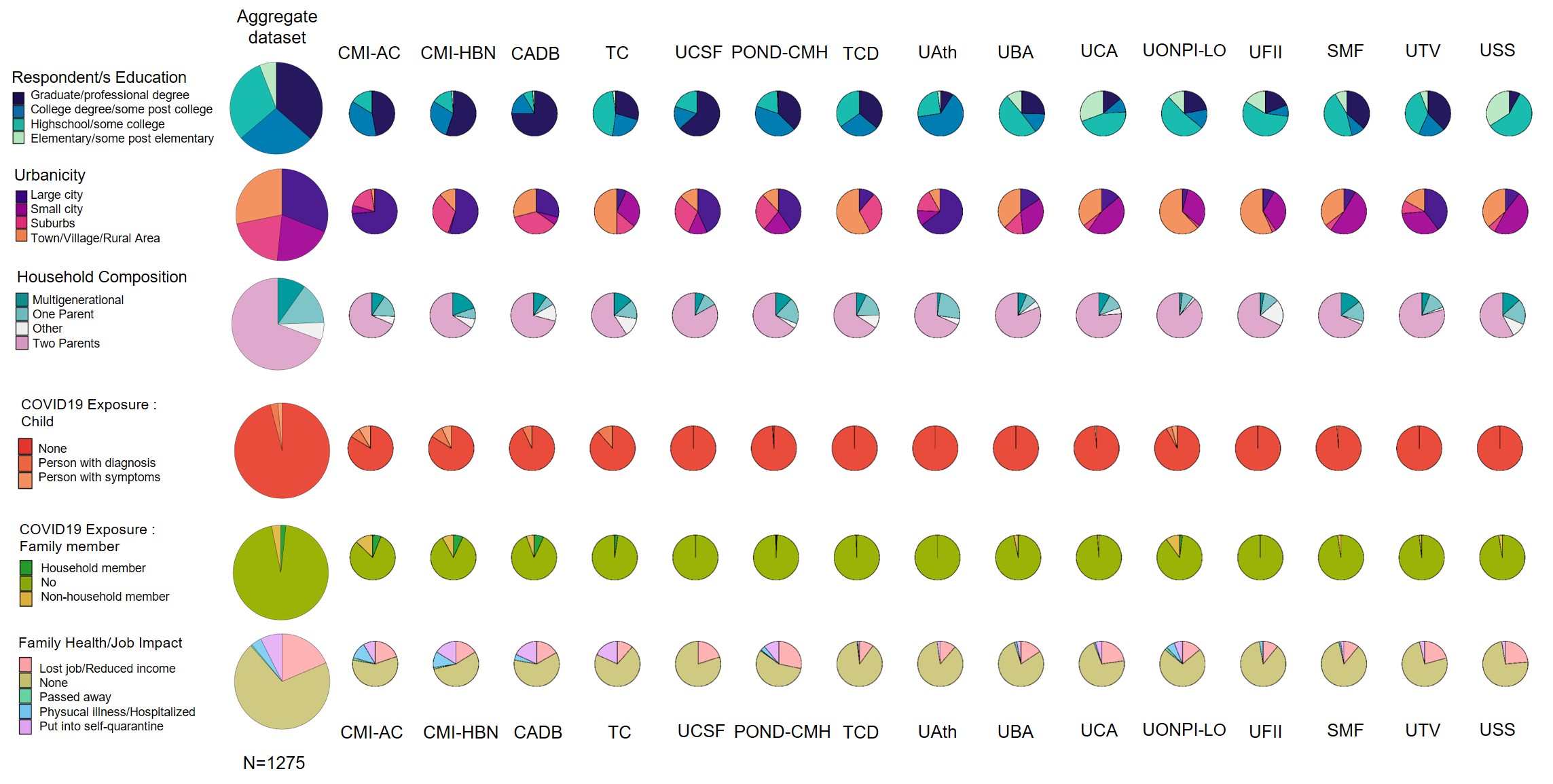
Fig S2. CRISIS AFAR Survey background and COVID-related information for the aggregate and each contributing sample.**

Larger pies show the aggregate dataset information (N=1275), and smaller pies each of the contributing samples. Of them, the first 4 samples from the right include those collected in USA institutions (CMI-AC, CMI-HBN, CADB, TC and UCSF), followed by the one collected in Canada (POND-CMH), Ireland (TCD), and Greece (UAth), and the f7 samples from Italian institutions (UBA, UCA, UONPI-LO, UFII, SMF, UTV, USS). See Figure 1 for each sample label. The first 3 rows refer to the background survey questions: highest level of respondent education (first or second caregiver), urbanicity and household composition; the 3 bottom rows illustrate COVID-19 related factors: child and family member exposure as well as family impact on health and job. Abbreviations: see Table S1 for site name abbreviated labels.

**
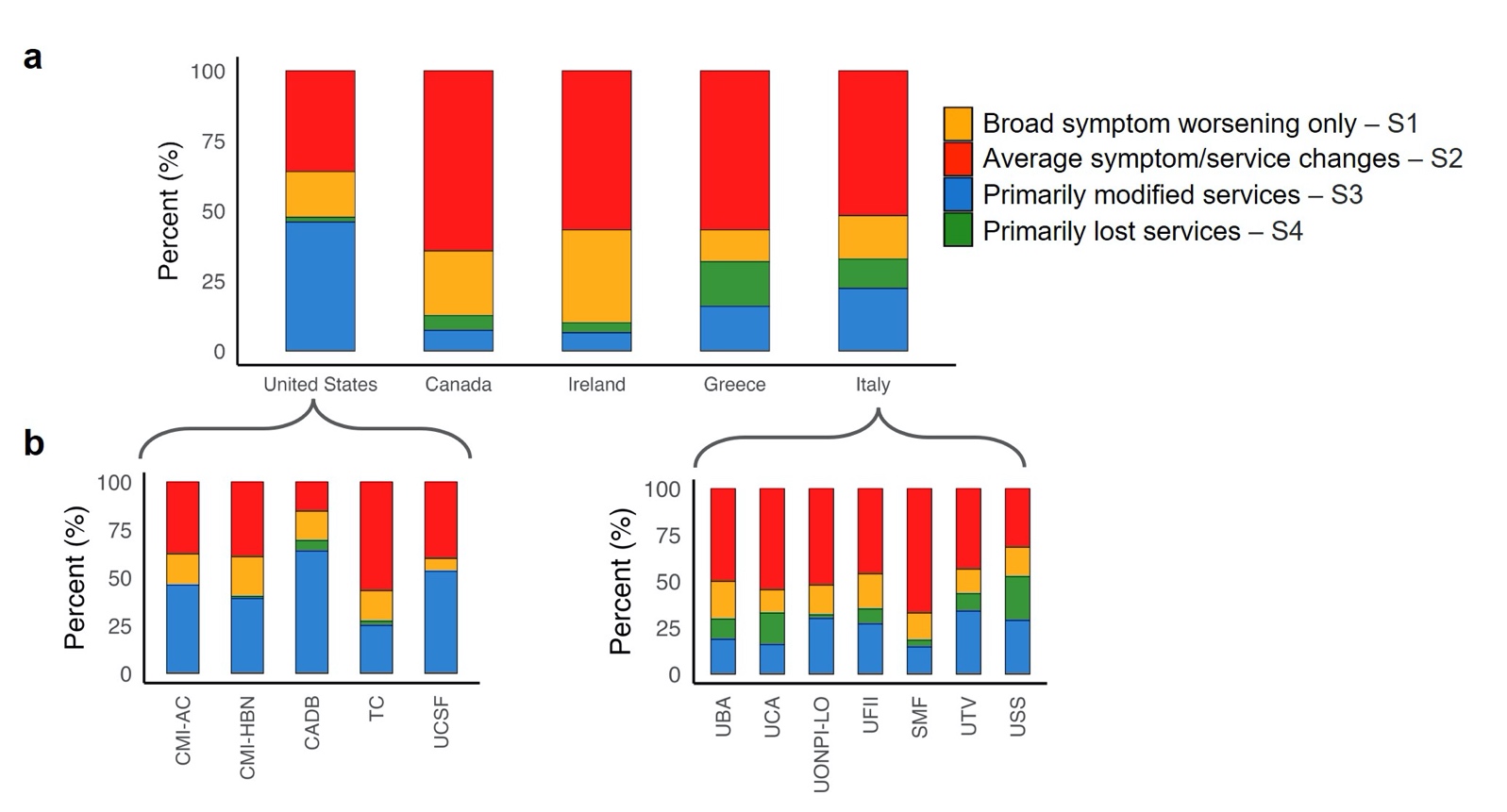
**

**Fig S3. Subgroup distribution by contributing sample.**

The percentages of children for each subgroup based on the total aggregate dataset (N=1275) by Nation are shown (n=5: United States, Canada, Ireland, Greece, and Italy (A) and contributing sample in the United States (left, n=5) and Italy (right, n=7) (B). Abbreviations: see Table S1 for site name abbreviated labels.

**
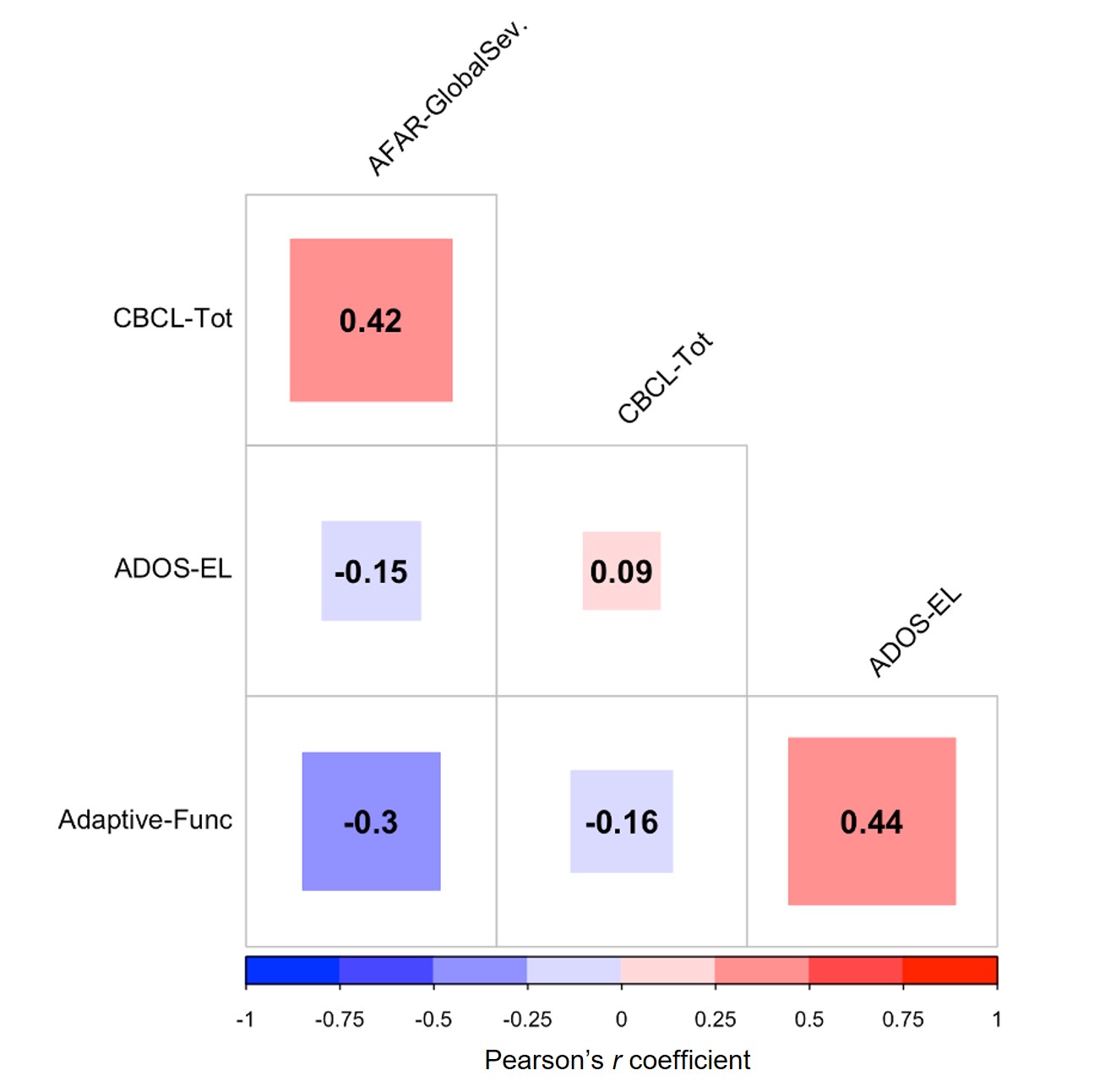
**

**Fig S4. Correlations between pre-pandemic standardized severity measures and AFAR baseline global severity score.**

Correlations were calculated across N=453 children with complete observations from 9 contributing samples. The size of the squares within the matrix is proportional to the Pearson’s r coefficient values (i.e., larger squares indicate stronger correlations). The color scale indicates whether the correlation is positive (in red) or negative (in blue). Of note, all correlations with AFAR global severity scores (“AFAR-GlobalSev.”) are statistically significant at p<0.0001 after FDR-correction. Abbreviations: AFAR-GlobalSev., global severity prior to the COVID-19 pandemic (see Methods in Supplementary for details on its calculation); CBCL-Tot, CBCL T total scores; ADOS-EL, ADOS-2 Expressive language scores; Adaptive-Func, composite scores of adaptive functioning.

**References**

1. Nikolaidis A, Paksarian D, Alexander L, et al. The Coronavirus Health and Impact Survey (CRISIS) reveals reproducible correlates of pandemic-related mood states across the Atlantic. *Sci Rep*. 2021;11(1):8139.

2. Ressel M, Thompson B, Poulin MH, et al. Systematic review of risk and protective factors associated with substance use and abuse in individuals with autism spectrum disorders. *Autism*. 2020;24(4):899-918.

3. Piehler TF, Lee SK, Stockness A, Winters KC. The correspondence of parent-reported measures of adolescent alcohol and cannabis use with adolescent-reported measures: A systematic review. *Subst Abus*. 2020;41(4):437-450.

4. Valenti M, Ciprietti T, Egidio CD, et al. Adaptive response of children and adolescents with autism to the 2009 earthquake in L’Aquila, Italy. *J Autism Dev Disord*. 2012;42(6):954-960.

5. Lai MC, Kassee C, Besney R, et al. Prevalence of Co-Occurring Mental Health Diagnoses in the Autism Population: A Systematic Review and Meta-Analysis. *SSRN Electronic Journal*. doi:10.2139/ssrn.3310628

6. Grzadzinski R, Huerta M, Lord C. DSM-5 and autism spectrum disorders (ASDs): an opportunity for identifying ASD subtypes. *Mol Autism*. 2013;4(1):12.

7. Simonoff E, Pickles A, Charman T, Chandler S, Loucas T, Baird G. Psychiatric disorders in children with autism spectrum disorders: prevalence, comorbidity, and associated factors in a population-derived sample. *J Am Acad Child Adolesc Psychiatry*. 2008;47(8):921-929.

8. Peters-Scheffer N, Didden R, Sigafoos J, Green VA, Korzilius H. Behavioral flexibility in children with autism spectrum disorder and intellectual disability. *Research in Autism Spectrum Disorders*. 2013;7(6):699-709. doi:10.1016/j.rasd.2013.02.016

9. Lai MC, Lombardo MV, Baron-Cohen S. Autism. *The Lancet*. 2014;383(9920):896-910. doi:10.1016/s0140-6736(13)61539-1

10. Leekam SR, Prior MR, Uljarevic M. Restricted and repetitive behaviors in autism spectrum disorders: a review of research in the last decade. *Psychol Bull*. 2011;137(4):562-593.

11. Bishop SL, Hus V, Duncan A, et al. Subcategories of restricted and repetitive behaviors in children with autism spectrum disorders. *J Autism Dev Disord*. 2013;43(6):1287-1297.

12. American Psychiatric Association. *Diagnostic and Statistical Manual of Mental Disorders*. American Psychiatric Association; 2013.

13. Guttentag S, Bishop S, Doggett R, et al. The Utility of Parent-Report Screening Tools in Differentiating Autism vs. ADHD in School-age Children. doi:10.31234/osf.io/9pu7t

14. Kamara D, Beauchaine TP. A Review of Sleep Disturbances among Infants and Children with Neurodevelopmental Disorders. *Rev J Autism Dev Disord*. 2020;7(3):278-294.

15. Jeste S, Hyde C, Distefano C, et al. Changes in access to educational and healthcare services for individuals with intellectual and developmental disabilities during COVID-19 restrictions. *J Intellect Disabil Res*. Published online September 17, 2020. doi:10.1111/jir.12776

16. Baribeau DA, Dupuis A, Paton TA, et al. Structural neuroimaging correlates of social deficits are similar in autism spectrum disorder and attention-deficit/hyperactivity disorder: analysis from the POND Network. *Transl Psychiatry*. 2019;9(1):72.

17. Cost KT, Crosbie J, Anagnostou E, et al. Mostly worse, occasionally better: impact of COVID-19 pandemic on the mental health of Canadian children and adolescents. *Eur Child Adolesc Psychiatry*. Published online February 26, 2021. doi:10.1007/s00787-021-01744-3

18. Kushki A, Anagnostou E, Hammill C, et al. Examining overlap and homogeneity in ASD, ADHD, and OCD: a data-driven, diagnosis-agnostic approach. *Transl Psychiatry*. 2019;9(1):1-11.

19. Rosseel Y. Lavaan: An R package for structural equation modeling and more. Version 0.5--12 (BETA). *J Stat Softw*. 2012;48(2):1-36.

20. Team R. Core (2020). *R: A language and environment for statistical*. Published online 2020. https://scholar.google.ca/scholar?cluster=11495894719434114347&hl=en&as_sdt=0,5&sciodt=0,5

21. Gatignon H. Confirmatory Factor Analysis. In: Gatignon H, ed. *Statistical Analysis of Management Data*. Springer New York; 2010:59-122.

22. MacCallum RC, Browne MW, Sugawara HM. Power analysis and determination of sample size for covariance structure modeling. *Psychol Methods*. 1996;1(2):130-149.

23. Tucker LR, Lewis C. A reliability coefficient for maximum likelihood factor analysis. *Psychometrika*. 1973;38(1):1-10.

24. Hu L, Bentler PM. Cutoff criteria for fit indexes in covariance structure analysis: Conventional criteria versus new alternatives. *Struct Equ Modeling*. 1999;6(1):1-55.

25. Bentler PM. Comparative fit indexes in structural models. *Psychol Bull*. 1990;107(2):238-246.

26. Association AP, Others. DSM-4-TR. Published online 2004.

27. American Psychiatric Association DS, Association AP, Others. *Diagnostic and Statistical Manual of Mental Disorders: DSM-5*. Vol 5. American psychiatric association Washington, DC; 2013.

28. World Health Organization. *The International Statistical Classification of Diseases and Health Related Problems ICD-10: Tenth Revision. Volume 2: Instruction Manual*. World Health Organization; 2004.

29. Rutter M, Le Couteur A, Lord C, Others. Autism diagnostic interview-revised. *Los Angeles, CA: Western Psychological Services*. 2003;29(2003):30.

30. Lord C, Risi S, Lambrecht L, et al. The Autism Diagnostic Observation Schedule—Generic: A Standard Measure of Social and Communication Deficits Associated with the Spectrum of Autism. *J Autism Dev Disord*. 2000;30(3):205-223.

31. Lord C, Rutter M, DiLavore P, et al. Autism diagnostic observation schedule--2nd edition (ADOS-2). *Los Angeles, CA: Western Psychological Corporation*. 2012;284.

32. Kaufman J, Birmaher B, Brent D, et al. Schedule for Affective Disorders and Schizophrenia for School-Age Children-Present and Lifetime Version (K-SADS-PL): initial reliability and validity data. *J Am Acad Child Adolesc Psychiatry*. 1997;36(7):980-988.

33. Sparrow SS, Cicchetti D, Saulnier C. Vineland adaptive behavior scales--third edition (Vineland-3). *Circle Pines, MN: American Guidance Service*. Published online 2016.

34. Harrison PL, Oakland T. *ABAS-3*. Western Psychological Services Torrance; 2015.

35. Mazurek MO, Baker-Ericzén M, Kanne SM. Brief Report: Calculation and Convergent and Divergent Validity of a New ADOS-2 Expressive Language Score. *Am J Intellect Dev Disabil*. 2019;124(5):438-449.

36. Achenbach TM. The Child Behavior Checklist and related instruments. *The use of psychological testing for treatment planning and outcomes assessment, 2nd ed*. 1507;2(1999):429-466.

37. Mullen EM. Mullen scales of early learning. Published online 1995. http://www.v-psyche.com/doc/special-cases/Mullen%20Scales%20of%20Early%20Learning.docx

38. Alwinesh MTJ, Joseph RBJ, Daniel A, et al. Psychometrics and utility of Psycho-Educational Profile-Revised as a developmental quotient measure among children with the dual disability of intellectual disability and autism. *J Intellect Disabil*. 2012;16(3):193-203.

39. Gotham K, Pickles A, Lord C. Standardizing ADOS scores for a measure of severity in autism spectrum disorders. *J Autism Dev Disord*. 2009;39(5):693-705.

40. Sparrow SS, Balla DA, Cicchetti DV, Harrison PL. Vineland adaptive behavior scales. Published online 1984. http://www.disableddaughter.com/Pearlsky_vineland.pdf

41. Maechler M, Rousseeuw P, Struyf A, Hubert M, Hornik K. Cluster: Cluster Analysis Basics and Extensions.(2021). R package version 2.1. 2—For new features, see the ‘Changelog’file (in the package source).

42. Murtagh F, Legendre P. Ward’s hierarchical agglomerative clustering method: Which algorithms implement ward’s criterion? *J Classification*. 2014;31(3):274-295.

43. Charrad M, Ghazzali N, Boiteau V, Niknafs A. NbClust: An R Package for Determining the Relevant Number of Clusters in a Data Set. *Journal of Statistical Software, Articles*. 2014;61(6):1-36.

44. Pedregosa F, Varoquaux G, Gramfort A, et al. Scikit-learn: Machine learning in Python. *the Journal of machine Learning research*. 2011;12:2825-2830.

45. *CRISIS-AFAR-Analyses: This Repository Includes the Code Accompanying the CRISIS-AFAR Paper*. Github Accessed November 7, 2022. https://github.com/ChildMindInstitute/CRISIS-AFAR-analyses

46. Alvarez-Fernandez S, Brown HR, Zhao Y, et al. Perceived social support in adults with autism spectrum disorder and attention-deficit/hyperactivity disorder. *Autism Res*. 2017;10(5):866-877.

47. Breiman L. Random Forests. *Mach Learn*. 2001;45(1):5-32.

48. Simhal AK, Filho JOA, Segura P, et al. Predicting multimodal MRI outcomes in children with neurodevelopmental conditions following MRI simulator training. *bioRxiv*. Published online January 30, 2021:2021.01.28.428697. doi:10.1101/2021.01.28.428697

49. Hale T, Petherick A, Phillips T, Webster S. Variation in government responses to COVID-19. *Blavatnik school of government working paper*. 2020;31:2020-2011.

50. Roser M, Ritchie H, Ortiz-Ospina E, Hasell J. Coronavirus pandemic (COVID-19). *Our world in data*. Published online 2020. https://ourworldindata.org/coronavirus/country/sweden?country=~SWE&fbclid=IwAR3JDc2cGwgfSuYly7pBg9hdMqlhwN--Z5UR1qjVWV_NB44KcPwwMexY9qM

51. Naqvi A. COVID-19 European regional tracker. doi:10.1101/2021.02.15.21251788
